# Real‐time analysis of the cancer genome and fragmentome from plasma and urine cell‐free DNA using nanopore sequencing

**DOI:** 10.15252/emmm.202217282

**Published:** 2023-11-09

**Authors:** Ymke van der Pol, Normastuti Adhini Tantyo, Nils Evander, Anouk E Hentschel, Birgit MM Wever, Jip Ramaker, Sanne Bootsma, Marieke F Fransen, Kristiaan J Lenos, Louis Vermeulen, Famke L Schneiders, Idris Bahce, Jakko A Nieuwenhuijzen, Renske DM Steenbergen, D Michiel Pegtel, Norbert Moldovan, Florent Mouliere

**Affiliations:** ^1^ Pathology Amsterdam UMC Location Vrije Universiteit Amsterdam Amsterdam The Netherlands; ^2^ Cancer Center Amsterdam, Imaging and Biomarkers Amsterdam The Netherlands; ^3^ Urology Amsterdam UMC Location Vrije Universiteit Amsterdam Amsterdam The Netherlands; ^4^ Amsterdam UMC Location University of Amsterdam, Center for Experimental and Molecular Medicine, Laboratory for Experimental Oncology and Radiobiology Amsterdam The Netherlands; ^5^ Cancer Center Amsterdam, Gastroenterology Endocrinology Metabolism Amsterdam The Netherlands; ^6^ Oncode Institute Amsterdam The Netherlands; ^7^ Pulmonology Amsterdam UMC Location Vrije Universiteit Amsterdam Amsterdam The Netherlands; ^8^ Present address: Cancer Research UK Cancer Biomarker Centre University of Manchester Manchester UK

**Keywords:** cancer, cell‐free DNA, fragmentomics, liquid biopsy, nanopore sequencing, Cancer, Chromatin, Transcription & Genomics, Computational Biology

## Abstract

Cell‐free DNA (cfDNA) can be isolated and sequenced from blood and/or urine of cancer patients. Conventional short‐read sequencing lacks deployability and speed and can be biased for short cfDNA fragments. Here, we demonstrate that with Oxford Nanopore Technologies (ONT) sequencing we can achieve delivery of genomic and fragmentomic data from liquid biopsies. Copy number aberrations and cfDNA fragmentation patterns can be determined in less than 24 h from sample collection. The tumor‐derived cfDNA fraction calculated from plasma of lung cancer patients and urine of bladder cancer patients was highly correlated (*R* = 0.98) with the tumor fraction calculated from short‐read sequencing of the same samples. cfDNA size profile, fragmentation patterns, fragment‐end composition, and nucleosome profiling near transcription start sites in plasma and urine exhibited the typical cfDNA features. Additionally, a high proportion of long tumor‐derived cfDNA fragments (> 300 bp) are recovered in plasma and urine using ONT sequencing. ONT sequencing is a cost‐effective, fast, and deployable approach for obtaining genomic and fragmentomic results from liquid biopsies, allowing the analysis of previously understudied cfDNA populations.

## Graphical abstract

Cell‐free DNA (cfDNA), a rising biomarker in oncology, can be used as a readout from a liquid biopsy. Current analytical methods employ short‐read DNA sequencing technologies. We designed a long‐read nanopore sequencing technique to analyze liquid biopsy samples.

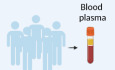

**The paper explained**

**Problem**

Current sequencing methods based on short‐read technologies to analyze cell‐free DNA (cfDNA) as a liquid biopsy in cancer patients have limitations. These include a bias toward shorter cfDNA populations and long waiting times for data. The Oxford Nanopore Technologies (ONT) platform offers a portable and fast alternative, capable of sequencing any length of cfDNA fragment. However, its potential for analyzing cfDNA from cancer patients remains underexplored.

**Results**

Plasma and urine samples from lung and bladder cancer patients, as well as healthy controls, were sequenced with a MinION ONT and compared to a short‐read sequencer. The ONT platform successfully retrieved cfDNA somatic copy number aberrations within 24 h of sampling, and with comparable sensitivity to short‐read technologies.

The study also demonstrated that ONT sequencing could recover long cfDNA fragments in both plasma and urine samples, contrary to the canonical belief that cfDNA is predominantly short and fragmented. The presence of tumor‐derived signal in these long fragments is confirmed in not only humans but also using a xenograft model. In addition to the size profile, other fragmentomic features could be retrieved from the cfDNA data.

**Impact**

In conclusion, the ONT sequencing provided a fast and accurate analysis of cfDNA from liquid biopsy samples. It allowed the detection of copy number aberrations, estimation of tumor fraction, and recovery of long cfDNA fragments. The ONT platform's deployability and short turnaround time make it a promising tool for liquid biopsy analysis.

## Introduction

Cell‐free DNA (cfDNA) is intensively investigated as a liquid biopsy in oncology (Heitzer *et al*, 2019). The genome and epigenome of cancer cells can be non‐invasively studied by recovering genetic or epigenetic alterations exhibited by cfDNA fragments (Wan *et al*, 2017; van der Pol & Mouliere, 2019). Current strategies based on either tumor‐naive or tumor‐guided sequencing are reaching high level of sensitivity for detecting tumor‐derived signal in plasma of cancer patients (Shen *et al*, 2018; Chabon *et al*, 2020; Wan *et al*, 2020; Zviran *et al*, 2020). They can also determine and leverage the biological properties of cfDNA (Mouliere *et al*, 2018; Lo *et al*, 2021; Hudecova *et al*, 2022).

Despite their successes, these approaches have limitations for a broad range of applications. They are based on short‐read sequencing technologies, inducing a bias toward shorter populations of cfDNA in the bloodstream. They require a complex and expensive sequencing platform, often shared by multiple research groups in genomic facilities, resulting in long waiting time before receiving cfDNA signal data. For a range of applications, for example, real‐time monitoring of patients under treatment or at‐home monitoring, more flexible, deployable, and fast liquid biopsy methods are needed.

Nanopore sequencing using the ONT platform is a highly portable and deployable technology that is capable of sequencing quickly any length of amplified or native DNA fragment or RNA molecules (Euskirchen *et al*, 2017; Katsman *et al*, 2022). Due to the higher rate of sequencing error in comparison to short‐read sequencers, the ONT platform has rarely been used for cfDNA mutation analysis (Cheng *et al*, 2015; Marcozzi *et al*, 2021). However, preliminary works show that broader genomic events, like copy number aberrations, can be recovered and analyzed with ONT nanopore sequencing of plasma cfDNA (Martignano *et al*, 2021).

Here, we show an ONT sequencing approach that can recover cfDNA somatic copy number aberrations (SCNA) and structural properties. Using ONT sequencing, we generated interpretable genomic copy number aberration with comparable sensitivity to short‐read technologies, nucleosome profile, and fragmentomic data in less than 24 h from blood collection. We applied the same framework, timeframe, and sequencing protocol to the analysis of urine cfDNA samples from bladder cancer patients. In addition to the genetic alterations, the ONT platform approach is improving the recovery of long cfDNA fragments, previously underestimated using short‐read technologies, opening a new window into cfDNA biology and structure.

## Results

### Copy number aberrations can be retrieved from the ONT platform data of plasma and urine samples within 24 h of sampling

**Figure 1:**
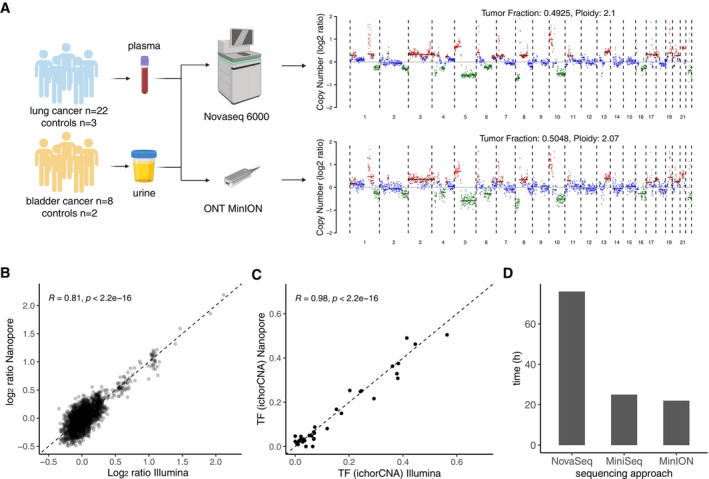
Analysis of genetic alterations from plasma and urine cfDNA with the ONT platform Schematic of the study workflow. Twenty‐five plasma samples from lung cancer patients (*n* = 22), healthy controls (*n* = 3), and ten urine samples from bladder cancer patients (*n* = 8); controls (*n* = 2) were processed according to an Illumina and nanopore protocol. Illumina reads were randomly downsampled to match the read count of the nanopore reads. Copy number aberration plots were determined using ichorCNA, and exemplified for one patient.Pearson correlation of the log_2_ ratio calculated using ichorCNA per 1 Mbp genomic bins between a matched sample processed by Illumina sequencing and nanopore sequencing.Pearson correlation of the cfDNA tumor fraction (TF) calculated using ichorCNA between matched samples processed by Illumina sequencing and nanopore sequencing.Turnaround time comparison in hours (h) among the NovaSeq, MiniSeq, and nanopore MinION protocols from sample collection to generation of interpretable data. The sample preparation phase includes collection, isolation, and DNA extraction. The computation phase includes base calling, demultiplexing, and data analysis. Source data are available online for this figure.

**Figure EV1:**
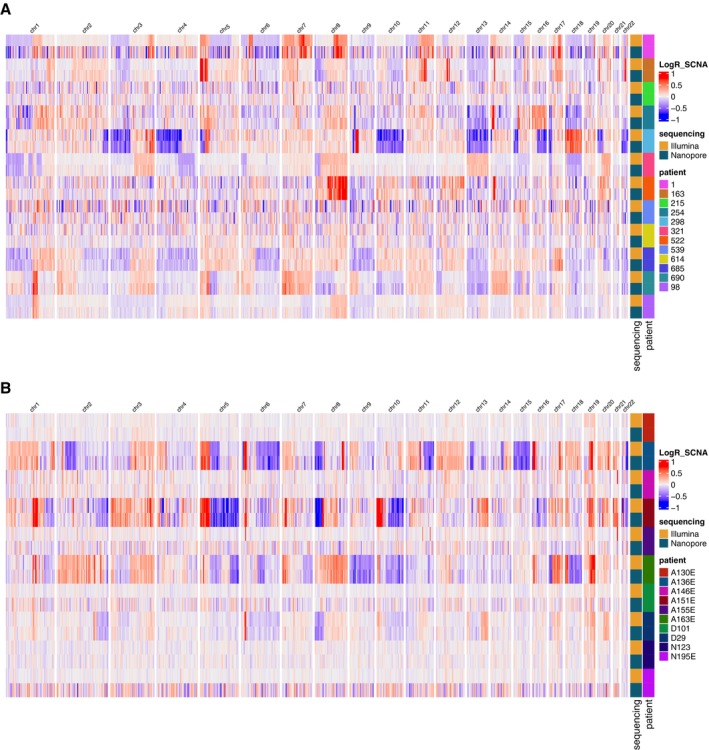
Copy number aberration heatmaps for the samples included in this study A, B(A) Late‐stage lung plasma samples and (B) urine samples. Illumina sequencing is downsampled randomly to the same number of reads as the nanopore data.

**Figure EV2:**
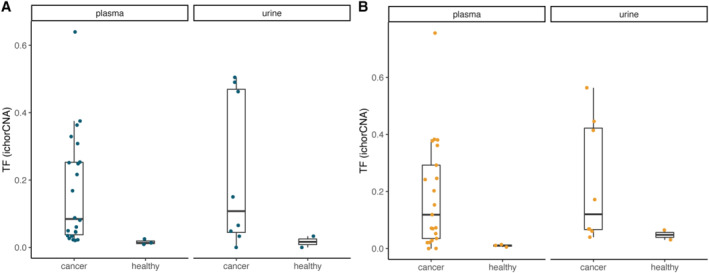
Tumor fraction by ichorCNA for the cancer and healthy samples included in this study A, B(A) From the nanopore data and (B) from the Illumina data.

To evaluate the potential of ONT sequencing to accurately recover SCNAs from cfDNA, we selected plasma from 22 patients with lung cancer and three controls, and urine samples from eight patients with bladder cancer and two non‐cancer controls (Fig 1A and Table EV1). An aliquot from the same samples was sequenced with a short‐read approach for comparison (see Methods). The ONT platform yielded a median of 800,183 passed mapping reads with an average coverage of ~0.1X, while NovaSeq sequencing yielded a median of 65,231,322 mapping read pairs with an average coverage of ~4.09X. After sequencing and read processing, SCNA plots were generated with the ichorCNA software (Adalsteinsson *et al*, 2017). We designed a new computational tool called ITSFASTR (InTegrated Sequence and Fragmentome AnalysiS Time Reduction) for the reproducible analysis of genomic and fragmentomic patterns (see Materials and Methods). Similar genomic events could be observed in both short‐ and long‐read data as illustrated for one patient with lung cancer (Fig 1A) and for the whole dataset (Fig EV1). The amplitude of copy number aberrations per genomic bins was correlated between nanopore and Illumina data, as illustrated in Fig 1B for one patient (Fig EV1 and Appendix Fig S1). The cfDNA tumor fraction, determined using ichorCNA, was highly correlated between the nanopore and Illumina data (Pearson *R* = 0.98, *P* < 0.001), irrespective of the type of biofluid used (plasma or urine) (Fig 1C). None of the controls tested had detectable tumor signal (> 3% tumor fraction (Adalsteinsson *et al*, 2017)) using ichorCNA (0/5) compared to the 74% of cancer cases (22/30) for both nanopore and Illumina data (Fig EV2). To test if the ichorCNA tumor fraction estimates were affected by coverage, we created an *in silico* admixture of 25 Illumina and 25 nanopore samples with a tumor fraction of ~15% and downsampled them iteratively from 1 M reads to 50,000 reads. We then computed the tumor fraction of each downsampled sample and found the lower limit of detection being between a depth of 500,000 and 100,000 mapped reads for both short and long reads (Appendix Fig S2).

The flexibility of the ONT platform allowed fast turnaround time using an integrated framework for liquid biopsy analysis. The time needed from blood collection to interpretable genomic data was ~24 h in total (including ~6 h of hands‐on experiments and ~18 h of sequencing and computation time on a laptop with average specifications (see Materials and Methods), the main bottleneck being the base calling step (Fig 1D)). This can be dramatically reduced to real‐time speeds by using a PC with advanced graphical processing unit (GPU) for base calling (Wick *et al*, 2019), improving the turnaround time to ~7 h. We calculated the theoretical time needed for generating data on the same samples with a short‐read NovaSeq pipeline to ~84 h, and with a MiniSeq to ~24 h, assuming immediate availability of all required machines and staff to operate them. In practice, the sequencing of an aliquot from the same samples using NovaSeq has taken an average of 34 days (range 31–38 days) from blood collection to interpretable sequencing data due to multiple waiting steps (e.g., before DNA sequencing, for data demultiplexing). The use of ONT sequencing can reduce the turnaround time to analyze liquid biopsy samples.

### Long cfDNA can be retrieved in the plasma and urine of cancer patients with nanopore sequencing and contains tumor signal

Canonically, cfDNA in blood plasma has been described as short, fragmented molecules centered around 167 bp (and multiple) due to enzymatic cleavage linked to cell death (Jahr *et al*, 2001; van der Pol & Mouliere, 2019; Han *et al*, 2020; Mouliere, 2022). In urine, cfDNA was described as even shorter with a median of around 82 bp and the lack of a nucleosome‐bound pattern (Markus *et al*, 2021; Mouliere *et al*, 2021). This structural organization, observed using short‐read sequencing technologies, has been recently challenged by using alternative single‐stranded DNA sequencing library preparation (Hudecova *et al*, 2022), or sequencing technique (Yu *et al*, 2021).

**Figure 2:**
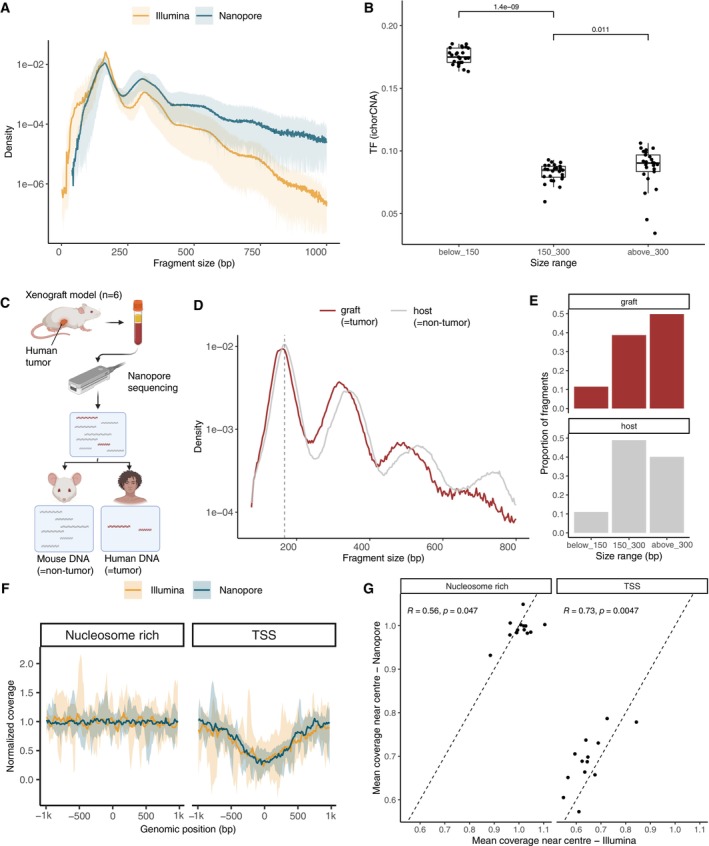
Plasma cfDNA fragmentation patterns using nanopore and short‐read sequencing in human and xenografts In blue are shown nanopore data and in orange Illumina data. Illumina data are downsampled to match ONT coverage.
Median density of cfDNA fragment size (bp) from plasma samples with lung cancer (*n* = 22).Tumor fraction (TF) of the *in silico* admixtures from plasma samples calculated with ichorCNA depending on specific cfDNA fragment size ranges. *P*‐values calculated using two‐sided Wilcoxon test. Horizontal line within the bars represents median of the underlying population. Boxplot whiskers show 1.5 interquartile range of highest and lowest quartiles.Schematic of xenograft model collection.Median density of cfDNA fragment size (bp) from plasma samples of xenograft models sequenced by nanopore.Proportion of graft (= tumor) and host (= non‐tumor) cfDNA fragments depending on selected size ranges.Normalized coverage of cfDNA from plasma samples of lung cancer patients near TSS regions (± 1 kbp), and near nucleosome‐rich regions (± 1 kbp). Lines showed the median coverage, and ribbon the minima and maxima values.Pearson correlation of the mean coverage near center of nucleosome‐rich and TSS regions by Illumina and nanopore sequencing.
Source data are available online for this figure.

Using ONT sequencing on plasma from lung cancer patients, the typical nucleosome‐bound cfDNA pattern can be observed with a mode of distribution centered around 167 bp and multiple thereof (Fig 2A and Appendix Fig S3). The recovery of long cfDNA (> 300 bp, supposedly linked to di‐ and trinucleosomes) in the plasma of lung cancer patients was increased, as 54.1% of the fragments were longer than 300 bp with the ONT platform compared to 5.3% with short‐read sequencing (Fig 2A). The diversity of the trinucleotides at the end of these long cfDNA fragments, determined using Gini index, was not different from the one in shorter (150–300 bp) size ranges (Appendix Fig S3), suggesting that the sequencing was not affecting the entropy of the recovered fragment end sequences. Moreover, long cfDNA fragments (> 300 bp) contained ctDNA tumor signal at increased tumor fraction compared to shorter cfDNA sizes (150–300 bp), but not compared to ultra‐short fragments (< 150 bp), as demonstrated following *in silico* admixture creation and copy number aberration analysis of long cfDNA (see Materials and Methods, Fig 2B). We observed a decreased fragment‐end diversity for shorter fragments (< 100 bp) (Appendix Fig S4), probably due to low yields at this size range. To bypass this limitation, we tested a modification of the protocol (see Materials and Methods) which improved the recovery of high‐diversity cfDNA fragment ends in the short size range (Appendix Fig S4), and mimicked the size profile proportions observed using short‐read technologies. However, the cfDNA fragment‐end sequence composition was altered compared to short‐read sequencing. For ONT sequencing, the proportion of fragments starting with a T was the highest with a mean of 0.34 compared to 0.32 in the paired short‐read dataset, followed by C with a mean of 0.26, compared to 0.27 (Appendix Fig S5). The ONT protocol used also affected the fragment‐end sequence composition (Appendix Fig S5).

It is challenging to specifically analyze the tumor signal in human samples. High‐depth sequencing and careful filtering of known mutations are required to distinguish cfDNA molecules originating from cancer cells. Using xenograft models (*n* = 3), we determined with maximal specificity the size profile of plasma tumor DNA (= human DNA) and non‐tumor DNA (= mouse DNA) (Fig 2C). We confirmed the presence of tumor‐derived signal in long fragments of cfDNA (> 300 bp) up to 8,055 bp (Fig 2D). On average, nearly ~50% of the graft human cfDNA (= tumor) was > 300 bp, in comparison to ~40% of the host mouse cfDNA (=non‐tumor) (Fig 2E). The 20 bp shift toward shorter cfDNA sizes of tumor DNA, previously observed on the mononucleosome level, progressively increased to > 100 bp for the dinucleosome, the trinucleosome sizes, and for tetranucleosome sizes (Fig 2D).

Beyond cfDNA fragmentation, we evaluated the potential of ONT sequencing to recover other features, in particular the nucleosome profile. We calculated the coverage around a set of known transcription start sites (TSS, *n* = 6,280 sites) and nucleosome‐covered quiescent sites, or nucleosome “rich” regions (NRR, *n* = 8,001) as negative controls using a modified version of Griffin (preprint: Doebley *et al*, 2021). The central coverage in the ± 500 bp region around TSSs was decreased, but as expected not around the NRR regions (Fig 2F and Appendix Figs S6 and S7). This profile was consistent for the *in silico* downsampled short‐read and long‐read plasma data sets. The mean coverage in the ± 1,000 bp vicinity of the TSS sites of interest shows a moderate correlation between short‐ and long‐read samples (*R* = 0.73, *P* = 0.0047) (Fig 2G).

**Figure 3:**
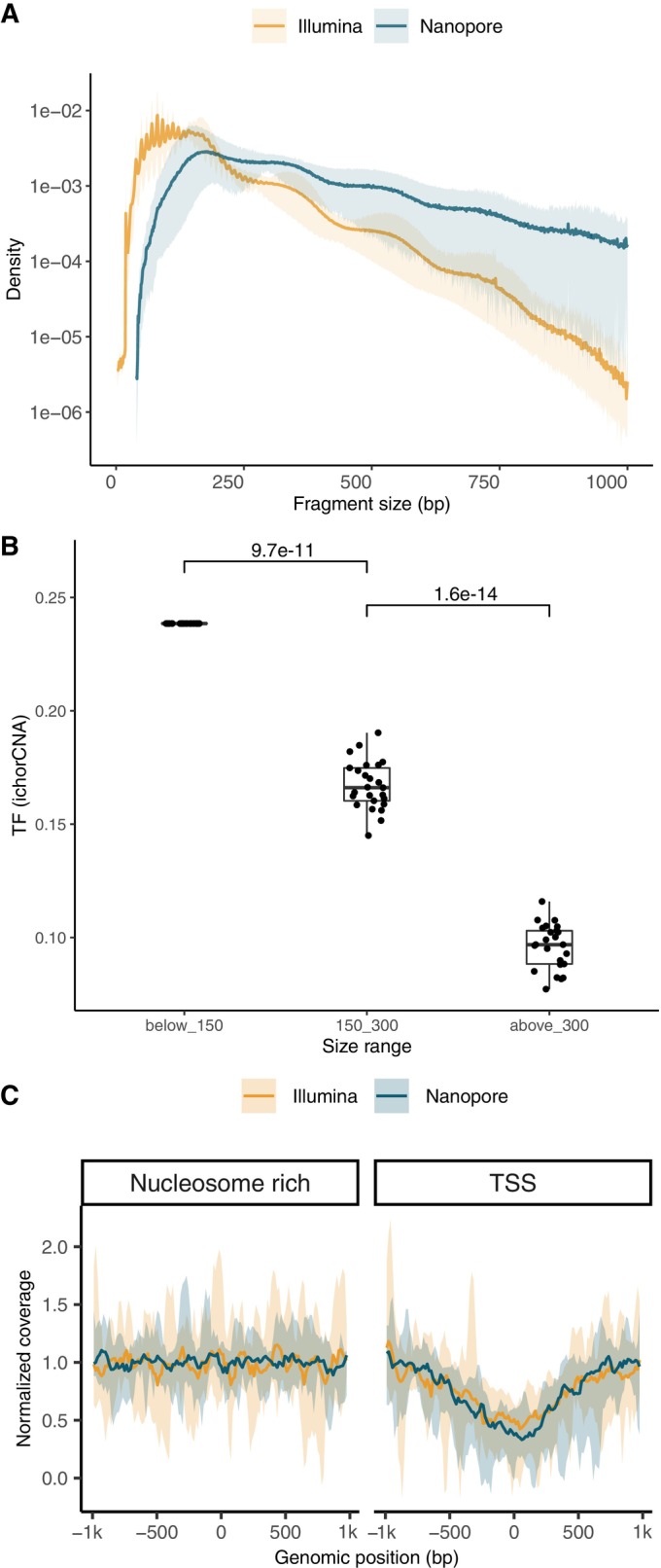
Urine cfDNA fragmentation patterns using nanopore and short‐read sequencing In blue are shown nanopore data and in orange Illumina data. Illumina data are downsampled to match ONT coverage.
Median density of cfDNA fragment size (bp) from urine samples with bladder cancer (*n* = 8).Tumor fraction (TF) of the urine samples calculated with ichorCNA depending on specific cfDNA fragment size ranges. *P*‐values were calculated using two‐sided Wilcoxon test. Horizontal line within the bars represents median of the underlying population. Boxplot whiskers show 1.5 interquartile range of highest and lowest quartiles.Normalized coverage of cfDNA from urine samples of bladder cancer patients near TSS regions (± 1 kbp), and near nucleosome‐rich regions (± 1 kbp). Lines showed the median coverage, ribbon the minima and maxima values.
Source data are available online for this figure.

Applied to urine samples, ONT sequencing recovered cfDNA fragments exhibiting the typical non‐nucleosomal patterns previously observed (Markus *et al*, 2021) using short‐read methods for this biofluid (Fig 3A and Appendix Fig S8). However, the median proportion of long cfDNA fragments (> 300 bp) was higher using ONT sequencing in comparison to short‐read data (62.4% and 14.2%, respectively). Fragments up to 4.28 kbp were detected in the urine of a patient. Similarly, to plasma, > 300 bp cfDNA contained the same fraction of ctDNA tumor signal compared to shorter cfDNA, previously missed by short‐read ligation‐based sequencing (see Materials and Methods, Fig 3B). The coverage profiles near TSS and NRR were also consistent between long‐read and short‐read data from the urine samples (Fig 3C and Appendix Figs S9 and S10). This demonstrates that ONT sequencing can preserve the nucleosome profile information from liquid biopsy samples and that the tumor signal is not exclusive to short cfDNA fragments.

## Discussion

We introduced an integrated computational framework based on an ONT sequencing platform that allows analysis of the cancer genome and fragmentome using plasma and urine cfDNA from liquid biopsy in less than 24 h from sample collection. This framework generates copy number aberration plots and an estimation of the ctDNA tumor fraction that is highly correlated with what could be achieved using a conventional short‐read sequencing framework for liquid biopsy analysis. The capacity of ONT native sequencing to extract a broad range of genomic, epigenomic, and transcriptomic features from the same sequencing run is an important advantage to more specialized methods (Euskirchen *et al*, 2017; Katsman *et al*, 2022).

The ONT sequencing platform MinION is highly deployable and has a much lower starting investment cost (currently ~1 k $ against ~50 k $ for a MiniSeq, ~250 k $ for a NextSeq, and ~900 k $ for a NovaSeq). This has to be mitigated by the relatively higher cost/Gbp of the MinION data (~13.1 $/Gbp, compared to ~4.8 $/Gbp for Novaseq S4). Nevertheless, this could be of interest to low‐ to middle‐income countries for improving the implementation of cfDNA sequencing. Moreover, one of the potential advantages of ONT sequencing is the short turnaround time to deliver interpretable genomic results. In practice, on real‐world samples, we were able to recover copy number aberrations and fragmentomic data from blood or urine samples in less than 24 h, including ~18 h of computation. This overall turnaround time could be drastically reduced to ~7 h when base calling using a computer with higher‐end GPU, producing results on the same day (Wick *et al*, 2019). The capacity to stop the run and re‐use the flow cell once enough reads are secured is also advantageous and was used in our study. Recent reports have demonstrated a potential to characterize brain malignancies using tissue biopsy in less than 2 h from sample collection (Djirackor *et al*, 2021), and characterize leukemia subtypes with 5 min of sequencing (preprint: Sagniez *et al*, 2022). This is an important improvement in comparison to other platforms which in real‐world practice are slower to deliver interpretable results.

Beyond this gain in time to deliver results, the ONT sequencing platform (and ITSFASTR by extension) can explore long cfDNA molecules which were previously inefficient using short‐read sequencing technologies. A report using an alternative long‐read technology demonstrated the presence of long cfDNA molecules in plasma (Yu *et al*, 2021; Choy *et al*, 2022), but other reports using nanopore on cfDNA did not report longer fragments (Martignano *et al*, 2021; Katsman *et al*, 2022). Here, we confirm such long fragments (> 300 bp) in the plasma and identify them in the urine of patients with cancer. The sequencing resolution required to recover more detailed fragmentomic signal (e.g., nucleosome occupancy, transcriptomic features on gene level) from such long fragments could be improved by increasing the sequencing time and thus coverage at the expense of speed, costs, and flexibility. Moreover, we observed that these long fragments contain tumor‐derived ctDNA signal, which could be critical to recovery in clinical conditions when such tumor signal is scarce (e.g., minimal residual disease detection). Contrary to traditional short‐read sequencing approaches, the full length of the molecule is sequenced for every fragment size with the ONT sequencing platform, which can provide useful information about the allelic occurrence of mutations, or phasing (Suzuki *et al*, 2017). Using short‐read techniques, phased variants have shown potential to improve the sensitivity to detect minute amounts of tumor DNA in plasma (Kurtz *et al*, 2021), but the potential of long‐read techniques on phased variants analysis remains untapped.

Fragmentation‐based nucleosome profiling from plasma samples is a promising alternative to more conventional and labor‐intensive methods for the deconvolution of chromatin states in the genome (Snyder *et al*, 2016; Lo *et al*, 2021). We demonstrated that the nucleosome position profile is preserved and can be retrieved from plasma and urine data produced by ONT sequencing. Such information can be recovered from various biofluids, including plasma and urine samples. Nucleosome profiling enabled tissue‐of‐origin deconvolution from plasma samples of high‐depth WGS data (Snyder *et al*, 2016; Zhu *et al*, 2021). This could complement other approaches based on methylation analysis for determining the cell of origin of cfDNA in plasma (Lehmann‐Werman *et al*, 2016; Katsman *et al*, 2022). Thus, ONT sequencing could potentially power ultra‐fast and deployable nucleosome profile‐based tissue‐of‐origin deconvolution.

The sample size of this study is relatively small and will require further confirmation with larger cohorts of biofluids for validation in clinical situations. In particular, an application to clinical situation where high‐intensity sampling (e.g., multiple samples per week for follow‐up post‐surgery, or analysis of diurnal modifications) could help to demonstrate the potential of the approach. Per nature, ONT sequencing was used in a low‐coverage whole genome sequencing (lc‐WGS) mode which is affected by the same limitation in terms of sensitivity as lc‐WGS from short‐read technologies (Heitzer *et al*, 2013; Adalsteinsson *et al*, 2017). Although not affecting the detection of large genomic events or fragmentomic analysis, one drawback of ONT sequencing is the single read base accuracy, which is currently inferior to that of conventional short‐read approaches, and can affect SNV calling. Alternative approaches are under development to improve the recovery of SNV from plasma cfDNA using ONT sequencing (Marcozzi *et al*, 2021), and deeper sequencing coverage can be achieved using other ONT platforms which might be critical for tracking minimal residual disease. Moreover, the pre‐analytical conditions that are affecting cfDNA short‐read sequencing and fragmentomic analysis could alter longer cfDNA fragments differently (van der Pol *et al*, 2022).

In conclusion, same‐day delivery of genomic and fragmentomic signatures from plasma and urine cfDNA is possible via an integrated nanopore liquid biopsy framework. Using ONT sequencing, we identify high proportions of long fragments of cfDNA not only in the plasma but also in the urine of patients with cancer. We determined these long fragments contained tumor‐derived ctDNA molecules that could be important to recover for future liquid biopsy applications.

## Materials and Methods

### Study design

A total of 25 plasma and 10 urine samples from 35 individuals were retrieved across two cancer types and healthy individuals (Table EV1). Lung cancer patients were recruited following informed consent via the Liquid Biopsy Center at the Amsterdam UMC, location VUmc and location AMC (study approved by the Amsterdam UMC ethics board, METC U2019_035). Bladder cancer patients were recruited following informed consent at the Amsterdam UMC, location VUmc (study approved by the Amsterdam UMC Ethics Board, METC 2018.355). The study was not blinded and was carried out in accordance with the WMA Declaration of Helsinki and the Department of Health and Human Services Belmont Report.

### Sample preparation

Blood samples were collected before treatment in EDTA K2‐coated tubes and processed using a double‐centrifugation protocol (900 *g* for 7 min; 2,500 *g* for 10 min at room temperature). Supernatant plasma was aliquoted in 0.5 ml Nunc tubes before being stored at −80°C. Urine samples were collected before cystoscopy or transurethral resection of the bladder tumor and were processed within 24–72 h. DNA quality was preserved by the addition of 0.6 M EDTA to the collection tube in a final concentration of 40 mM. Urine samples were pelleted by centrifugation of 15 ml urine at 800 *g* for 10 min. The urine supernatant was collected and thereafter stored at −80°C. cfDNA from lung cancer plasma and bladder cancer urine samples were extracted using the automated system QiaSymphony Circulating DNA Kit (Qiagen) with 2–3.2 ml of sample input (PBS was added when required to reach 3.2 ml). Six matched plasma samples were extracted using MagMAX Cell‐Free DNA Isolation Kit (Thermo Fisher) according to the manufacturer's instructions with 1 ml plasma input. cfDNA concentration was measured using Cell‐free DNA ScreenTape Analysis of the Agilent 4200 TapeStation System (Agilent).

### Cell culture

Colorectal cancer cell line MDST8 was obtained from the Sanger Institute (Cambridge, UK) and cultured in Dulbecco's modified Eagle's medium/F‐12 medium with L‐glutamine, 15 mM HEPES (Thermo‐Fisher Scientific) supplemented with 10% v/v fetal bovine serum (Life Technologies), penicillin, and streptomycin. The cell line was authenticated by STR genotyping and regularly tested for mycoplasma infection.

### Xenograft model

Animal experiments were approved by the Animal Experimentation Committee at the Amsterdam UMC (location AMC) and conducted in accordance with the national guidelines. NOD.Cg‐Prkdc^scid^ Il2rg^tm1Wjl/Szj^ (NSG) mice were bred in‐house (*n* = 6). All animals were female, housed in a 12 h light/12 h dark cycle, with temperatures between 20°C and 24°C, 40–70% humidity, and 6–12 weeks old at time of the blood collection. Human CRC cells (50,000 cells/mice) in medium containing 50% matrigel (Corning) were injected intraperitoneally. Four weeks after tumor cell injection, blood collection via cardiac puncture under anesthesia was performed, immediately followed by euthanasia. Peritoneal tumor load was assessed using a scoring system equivalent to the peritoneal cancer index (PCI) that is used in humans. Plasma was isolated following the same protocol as for human samples. Two plasma samples were pooled together to increase starting volume during DNA isolation.

### Sequencing preparation

For short‐reads sequencing, ThruPLEX Plasma‐seq Kit (Takara Bio) was used for library preparation and sequenced after equimolar pooling using 150‐bp paired‐end mode on NovaSeq 6000 (Illumina) with S4 flow cells. Long‐read sequencing was performed using R9.4.1 flow cells on a MinION device (Oxford Nanopore Technologies). Two plasma sample libraries were prepared using the PCR Barcoding Kit (SQK‐PBK004, Oxford Nanopore Technologies) according to the manufacturer's instructions (Protocol 1). This protocol was adapted to short cfDNA (Protocol 2) by increasing the ethanol concentration to 80% on all washing steps and the amount of PCR cycles was set to 18 cycles to account for the low amount of cfDNA input. The beads‐to‐sample ratio was also altered to 1.8× according to a previously published study (Martignano *et al*, 2021). A second optimization was done by reverting the beads‐to‐sample ratio to the original SQK‐PBK004 protocol while keeping the amount of PCR cycles at 18 and ethanol concentration at 80% (Protocol 3). The quality of libraries was checked using the D1000 ScreenTape Analysis Assay of the Agilent 4200 TapeStation System (Agilent) prior to pooling. Libraries were pooled in equimolar amounts with 50–100 fmoles as the total input for sequencing.

### Base calling and demultiplexing

Base calling of the short‐read sequencing was performed using the NovaSeq control software (v.1.7.5). Demultiplexing was performed running bcl2fastq2 (v.2.20). Nanopore reads were base called and demultiplexed on a laptop with Intel i7‐7500U/BGA quad‐core processor, 8 GB DDR4 RAM, running Ubuntu LTS 20.04, and MinKNOW (v. 22.03.6), with Guppy (v. 6.0.7) running in CPU fast mode.

### The ITSFASTR tool

ITSFASTR (InTegrated Sequence and Fragmentome AnalysiS Time Reduction) was developed by our group (https://github.com/mouliere‐lab/ITSFASTR) and used in this study for integrating in a single fast‐turnaround time pipeline the read preprocessing, alignment, copy number analysis, tumor fraction estimation, and fragmentomic and nucleosome positioning analysis. ITSFASTR is compatible with short‐ and long‐read sequencing technologies.

### Read preprocessing

Short reads were trimmed using bbduk (bbmap v. 38.79 commit h516909a_0) with the ktrim = r, *k* = 23, mink = 11, hdist = 1 parameters, and default adapter sequences provided with the tool. Mapping to the human reference genome (GRCh38) was performed using BWA MEM (v. 0.7.17 commit hed695b0_7) with default parameters. Long reads were trimmed with Porechop (v. 0.2.4) with default parameters and adaptor sets, except extra_end_trimming, which was set to 0. Trimmed long reads from the patient samples were mapped to the human reference genome (GRCh38) using Minimap2 (v. 2.24) with the ax map‐ont parameter. Trimmed xenograft reads were mapped to the murine reference genome (GRCm38) or the human reference genome (GRCh38) using Minimap2 (v. 2.24) with the ax map‐ont parameter and sorted using Samtools (v. 1.12). Reads not mapping to either genome were removed and mapped reads with a mapping quality < 5 were excluded using Samtools (v. 1.12). A list of read names of alignments for each genome was created with the shell script ‘cut ‐f 1,5 | sort ‐k1,1 | uniq ‐f 1’. Then lists of read names unique to each genome were computed using the shell script ‘awk ‘BEGIN {OFS = “\t”} NR==FNR {a[$1] = $2; next} {if ($1 in a) {if ($2 > a[$1]) {delete a[$1]}}} END {for (i in a) print I}”. Finally, files containing alignments unique to the murine or the human genome were created using Samtools (v. 1.12) view, where the ‐N parameter took as input the previously generated list of unique read names. Duplicate reads from both short and long alignments were marked using Sambamba (v. 0.8.1) with default parameters. Unmapped reads, secondary or supplementary alignments, PCR duplicates, and alignments with a quality < 5 were removed using Samtools (v. 1.12). Short‐read datasets were downsampled to the same coverage as the long‐read data using Samtools (v. 1.12) view ‐bs i.p., where *i* = 95, a randomly generated number, while p was the proportion of long reads compared to the number of short reads.

### *In silico* admixture and downsampling

To generate a read count gradient, 12 long‐read samples and their short‐read pairs were used to create 25 *in silico* long‐ and corresponding short‐read admixtures with 1 M reads or read pairs using seqtk sample (v.1.3) with random seeds between 17 and 41 and the shell command cat. The resulting files were randomly downsampled using seqtk sample (v.1.3) with random seeds between 75 and 51 to 1,000,000, 500,000, 100,000, and 50,000, reads and read pairs, resulting in 25 samples each. To generate admixtures with fragments of a specific length and sufficient read count for tumor fraction estimation using ichorCNA, we merged, trimmed, and mapped plasma or urine cancer samples using the cat command. Subsequently, the merged reads were sorted based on their length into three categories: reads shorter than 150 bp, reads between 150 and 300 bp, and reads longer than 300 bp. Next, the size‐sorted and merged files were downsampled into 25 separate files using the seqtk sample tool (v.1.3) with random seeds ranging from 45 to 69. Each of the 25 files contained 500,000 reads, except for the urine samples with reads shorter than 150 bp, which contained only 300,000 reads, the total number available after merging.

### Copy number analysis and tumor fraction estimation

The ichorCNA software (v. 0.3.2.0) was used to perform the copy number analysis and estimate the ctDNA tumor fraction for both short‐ and long‐read data mapped to the human genome (Adalsteinsson *et al*, 2017). Exceptions to the software's default settings are as follows: (1) an in‐house panel of normals from shallow whole‐genome sequencing was created; (2) non‐tumor fraction parameter restart values were increased to c(0.95, 0.99, 0.995, 0.999); (3) ichorCNA ploidy parameter restart value was set to 2; (4) no states were used for subclonal copy number; and (5) the maximum copy number to use was lowered to 3. The tumor fraction with the highest log likelihood was retrieved and reported.

### Fragmentomic analysis

cfDNA fragment length was retrieved from the mapped short reads using Picard (v. 2.22.2) CollectInsertSizeMetrics with default parameters. Fragment lengths for the mapped long reads were computed using NanoPlot (v. 1.40.0) with the *alength*, *raw*, and *huge* parameters. Fragment‐end sequence proportions of the aligned reads were retrieved and their Gini index was calculated using our Fragment End Integrated Analysis (FrEIA) tool (commit 8ecb58b) [https://github.com/mouliere‐lab/FrEIA], with the *mode* parameter set to either *Illumina* for short reads or *ONT* for long reads, and FragmSizeMax set to 5,000 for the long‐read data.

Fragmentation‐based nucleosome positioning analysis was performed using a modified version of Griffin (commit 73c605a) [https://github.com/adoebley/Griffin] called Griffin‐LRS (preprint: Doebley *et al*, 2021). We modified the original code by adding the possibility of working with single‐end nanopore sequencing. We run Griffin‐LRS with the original parameters, except setting *map_quality* to 5 for both *griffin_GC_and_mappability_correcction* and *griffin_nucleosome_profiling*. GRCh38 was used as the reference genome. For the target sites, we retrieved the TSS positions for known genes (TssA regions) and nucleosome‐rich regions (NRR regions) from the UCSC website (https://hgdownload.soe.ucsc.edu/downloads.html#human) and filtered sites with low mappability, using *griffin_filter_sites*.

### Statistical analysis

The individual values of all experiments are also shown in the corresponding figures. The investigator was not blinded to the experimental conditions, samples were not randomized, and no sample was excluded from analysis. Statistical significance was determined using an unpaired two‐sided Wilcoxon test unless otherwise specified in the figure legends. The exact values of *n* and its representation for all experiments are indicated in Figure Legends. The levels of significance were as follows: *P < 0.05, **P < 0.01, and ***P < 0.001.

## Author contributions

**Ymke van der Pol:** Conceptualization; formal analysis; investigation; methodology; writing – original draft; writing – review and editing. **Normastuti Adhini Tantyo:** Formal analysis; investigation; methodology; writing – original draft; writing – review and editing. **Nils Evander:** Investigation; methodology; writing – review and editing. **Anouk E Hentschel:** Resources; writing – review and editing. **Birgit MM Wever:** Investigation; writing – review and editing. **Jip Ramaker:** Investigation; writing – review and editing. **Sanne Bootsma:** Resources; writing – review and editing. **Marieke F Fransen:** Resources; writing – review and editing. **Kristiaan J Lenos:** Resources; writing – review and editing. **Louis Vermeulen:** Resources; writing – review and editing. **Famke L Schneiders:** Resources; writing – review and editing. **Idris Bahce:** Resources; writing – review and editing. **Jakko A Nieuwenhuijzen:** Resources; writing – review and editing. **Renske DM Steenbergen:** Resources; writing – review and editing. **D Michiel Pegtel:** Funding acquisition; writing – review and editing. **Norbert Moldovan:** Conceptualization; data curation; software; formal analysis; validation; visualization; methodology; writing – original draft; writing – review and editing. **Florent Mouliere:** Conceptualization; resources; data curation; formal analysis; supervision; funding acquisition; validation; visualization; writing – original draft; project administration; writing – review and editing.

## Disclosure and competing interests statement

FM is co‐inventor on patents related to cfDNA analysis. Other co‐authors have no relevant conflict of interest.

## For more information

- https://www.amsterdamumc.org/en/research/institutes/cancer‐center‐amsterdam.htm
- https://moulierelab.org/

## Supporting information



Appendix

Expanded View Figures PDF

Table EV1

PDF+

Source Data for Figure 1

Source Data for Figure 2

Source Data for Figure 3

## Data Availability

Sequencing data are deposited at the EGA, accession number: EGAD00001009392. ITSFASTR (InTegrated Sequence and Fragmentome AnalysiS Time Reduction) is available at https://github.com/mouliere‐lab/ITSFASTR.
