# Supplementary material for: Real‐time analysis of the cancer genome and fragmentome from plasma and urine cell‐free DNA using nanopore sequencing
Source: EMBO Mol Med. 2023 Nov 9;15(12):e17282. doi: 10.15252/emmm.202217282 (PMC10701599; doi:10.15252/emmm.202217282)
Supplement: Supplementary file 1 — Appendix [file EMMM-15-e17282-s003.pdf]

Appendix files for:

Real-time analysis of the cancer genome and fragmentome from plasma and urine cell-free DNA using nanopore sequencing

Ymke van der Pol[1,2,*], Normastuti Adhini Tantyo[1,2,*], Nils Evander[1,2], Anouk E. Hentschel[1,3], Birgit M.M. Wever[1,2], Jip Ramaker[1,2], Sanne Bootsma[4,5,6], Marieke F. Fransen[2,7], Kristiaan J. Lenos[4,5,6], Louis Vermeulen[4,5,6], Famke L. Schneiders[2,7], Idris Bahce[2,7], Jakko A. Nieuwenhuijzen[2,3], Renske D.M. Steenbergen[1,2], D. Michiel Pegtel[1,2], Norbert Moldovan[1,2,#], Florent Mouliere[1,2,#]

*: co-first author
#: co-last author

1. Amsterdam UMC location Vrije Universiteit Amsterdam, Pathology, Amsterdam, the Netherlands.
2. Cancer Center Amsterdam, Imaging and Biomarkers, Amsterdam, the Netherlands.
3. Amsterdam UMC location Vrije Universiteit Amsterdam, Urology, Amsterdam, the Netherlands.
4. Amsterdam UMC location University of Amsterdam, Center for Experimental and Molecular Medicine, Laboratory for Experimental Oncology and Radiobiology, Amsterdam, the Netherlands.
5. Cancer Center Amsterdam, Gastroenterology Endocrinology Metabolism, Amsterdam, The Netherlands.
6. Oncode Institute, Amsterdam, The Netherlands.
7. Amsterdam UMC location Vrije Universiteit Amsterdam, Pulmonology, Amsterdam, the Netherlands.

Tables of contents

Page 3: Appendix Figure S1. Copy number aberration profiles for the samples included in this study.

Page 16: Appendix Figure S2. Accuracy of the tumor fraction (TF) calculation depending on the coverage.

Page 17: Appendix Figure S3. cfDNA fragment size distribution for the plasma samples included in this study.

Page 18: Appendix Figure S4. cfDNA fragment-end motif diversity calculated for each protocol tested in the study.

Page 18: Appendix Figure S5. cfDNA fragment-end mononucleotide proportions calculated for each protocol tested in the study.

Page 19: Appendix Figure S6. sequencing coverage near TSS regions of plasma samples.

Page 20: Appendix Figure S7. sequencing coverage near nucleosome-rich regions of plasma samples.

Page 21: Appendix Figure S8. cfDNA fragment size distribution for the urine samples included in this study.

Page 22: Appendix Figure S9. sequencing coverage near TSS regions of urine samples.

Page 23: Appendix Figure S10. sequencing coverage near nucleosome-rich regions of urine samples.

Appendix Figure S1

Patient 1 (LUAD_IV_009)

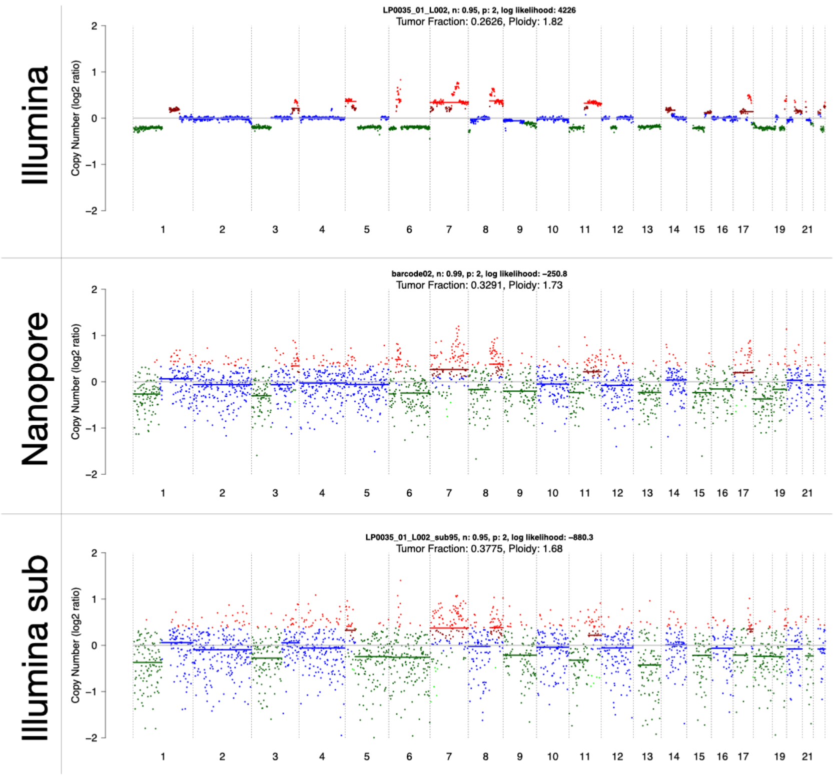

Patient 98 (LUAD_IV_007)

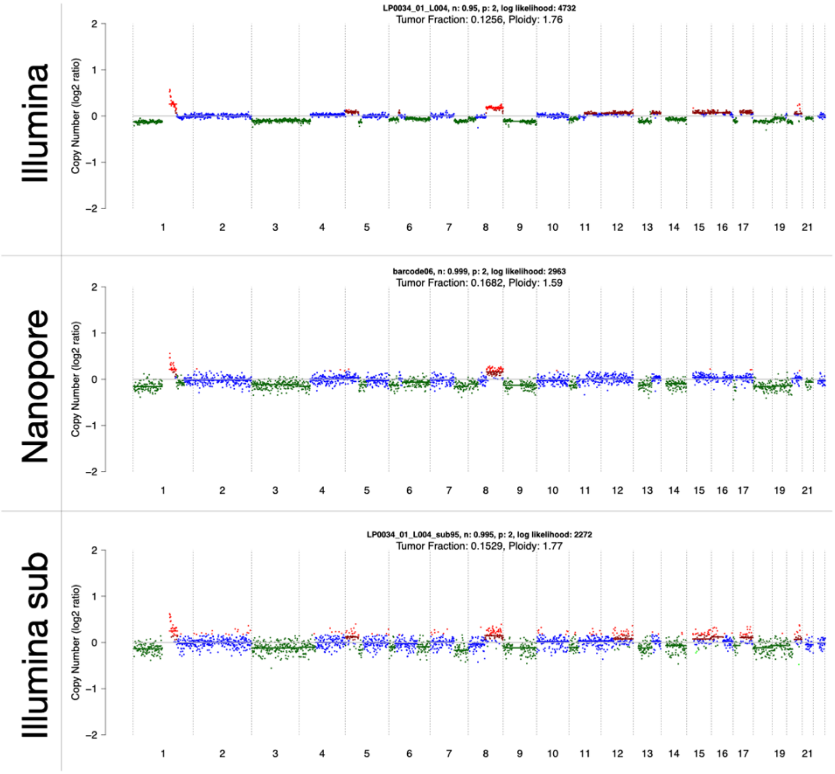

Patient 163 (LUAD_IV_008)

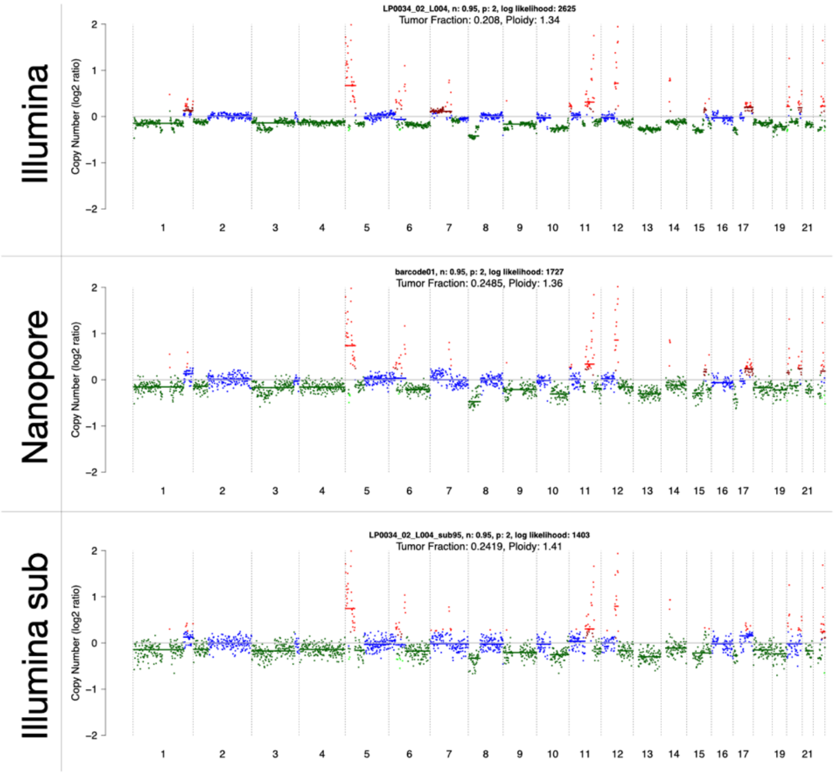

Patient 215 (LUAD_IV_001)

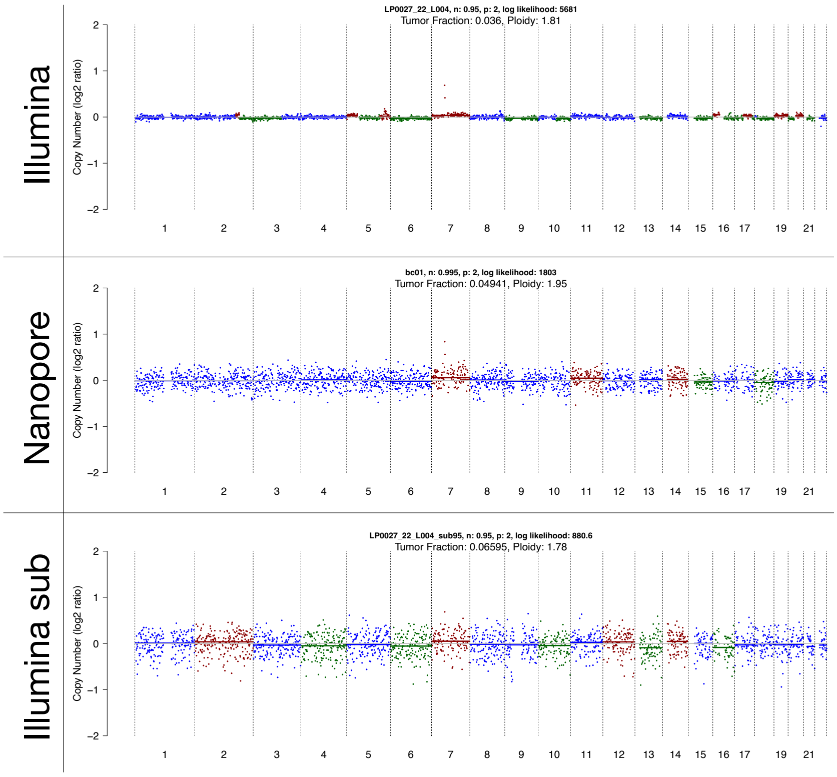

Patient 254 (LUAD_IV_004)

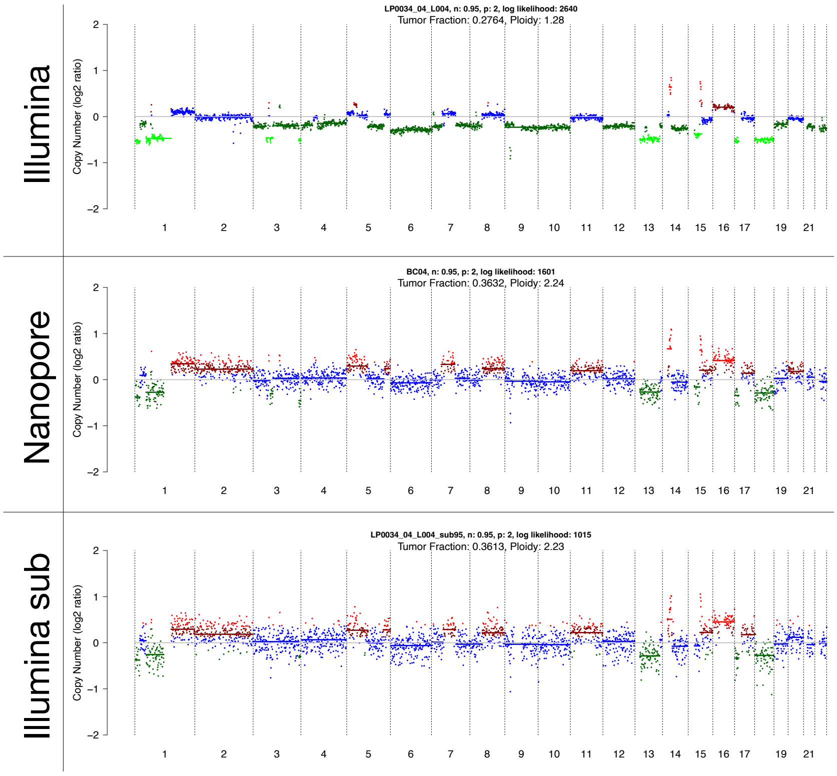

Patient 298 (LUAD_IV_003)

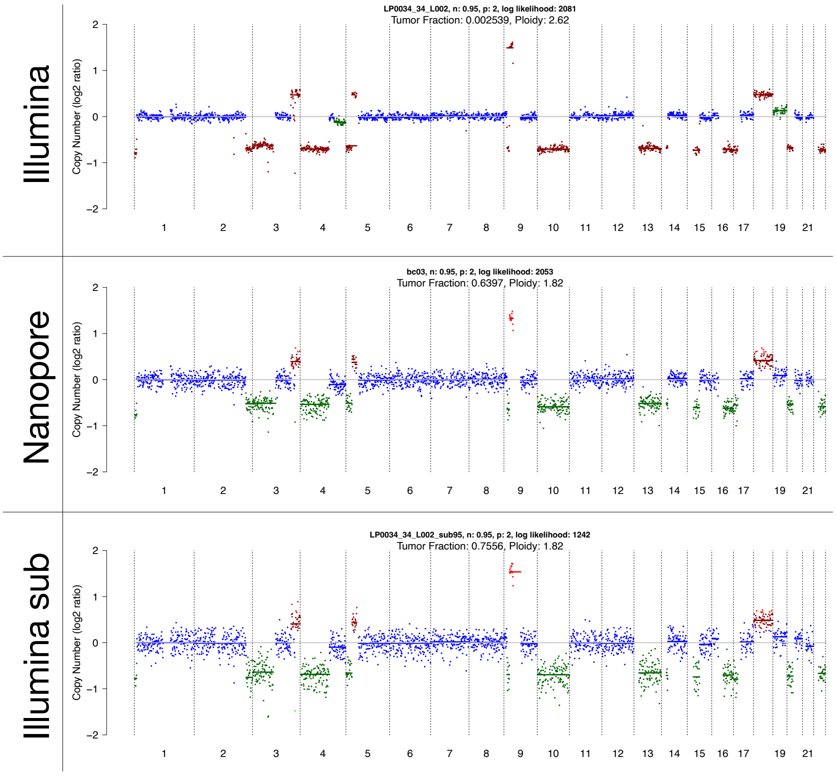

Patient 321 (LUAD_IV_011)

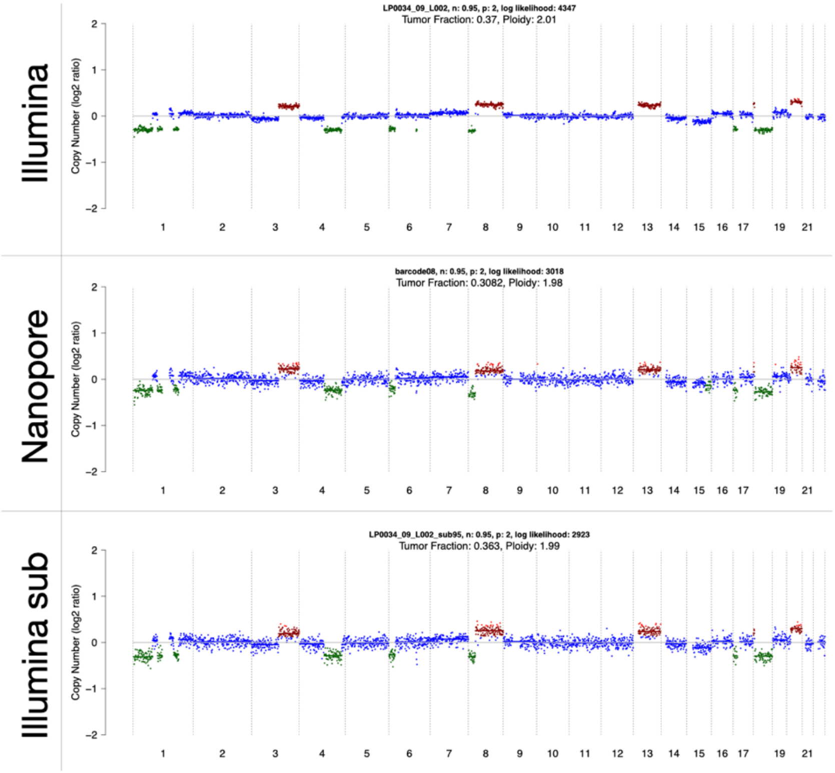

Patient 522 (LUAD_IV_005)

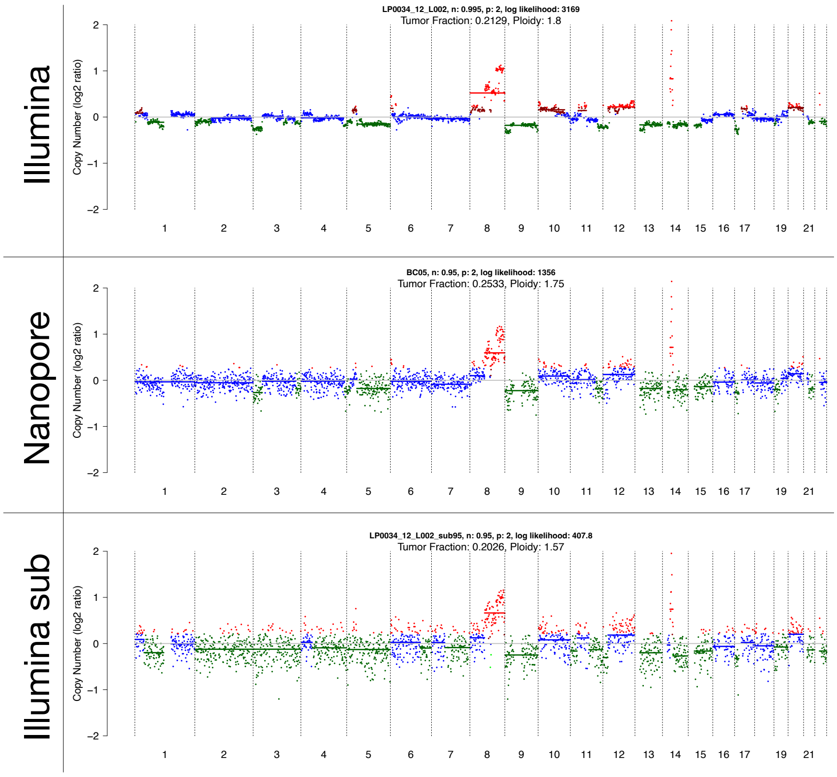

Patient 539 (LUAD_IV_006)

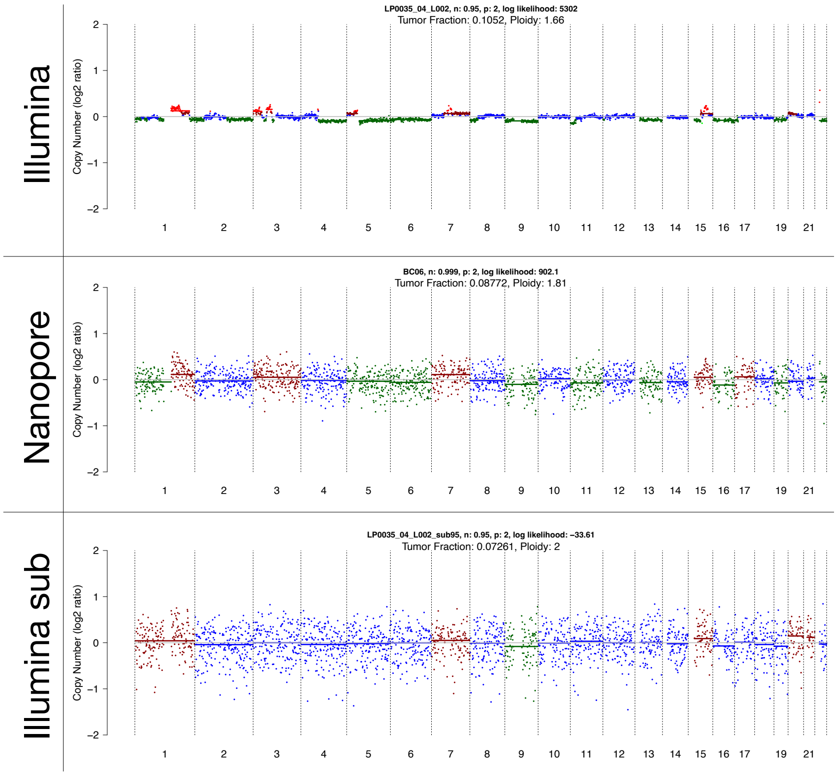

Figure 614 (LUAD_III_002)

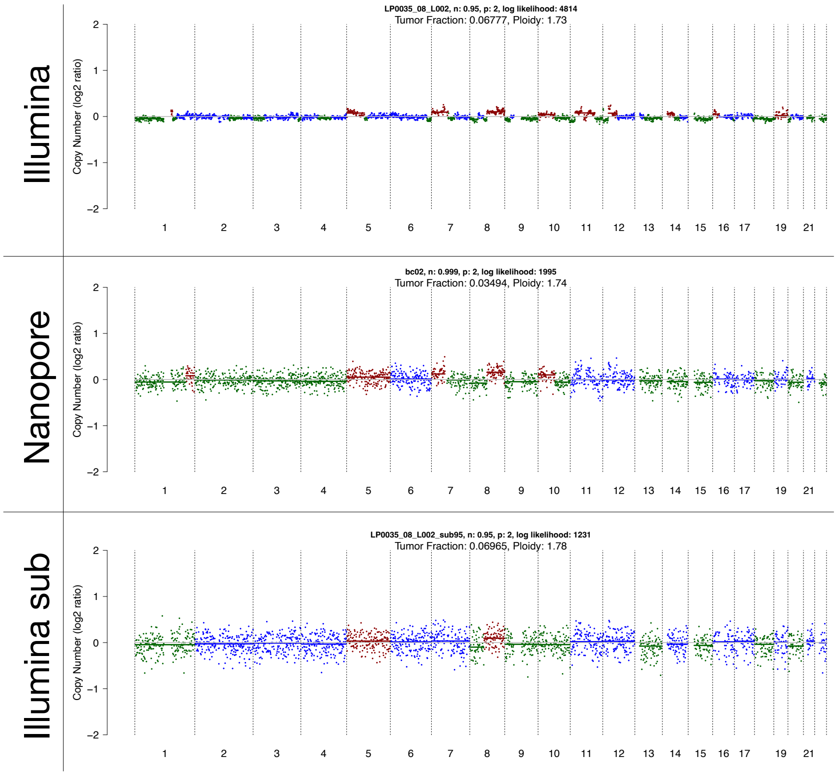

Patient 685 (LUAD_IV_012)

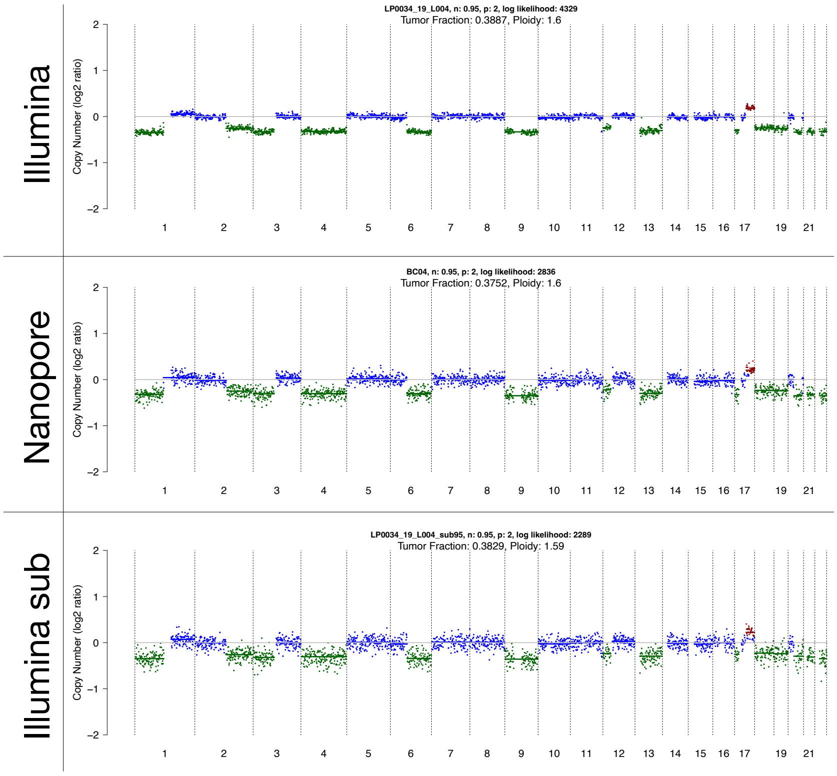

Patient 690 (LUAD_IV_010)

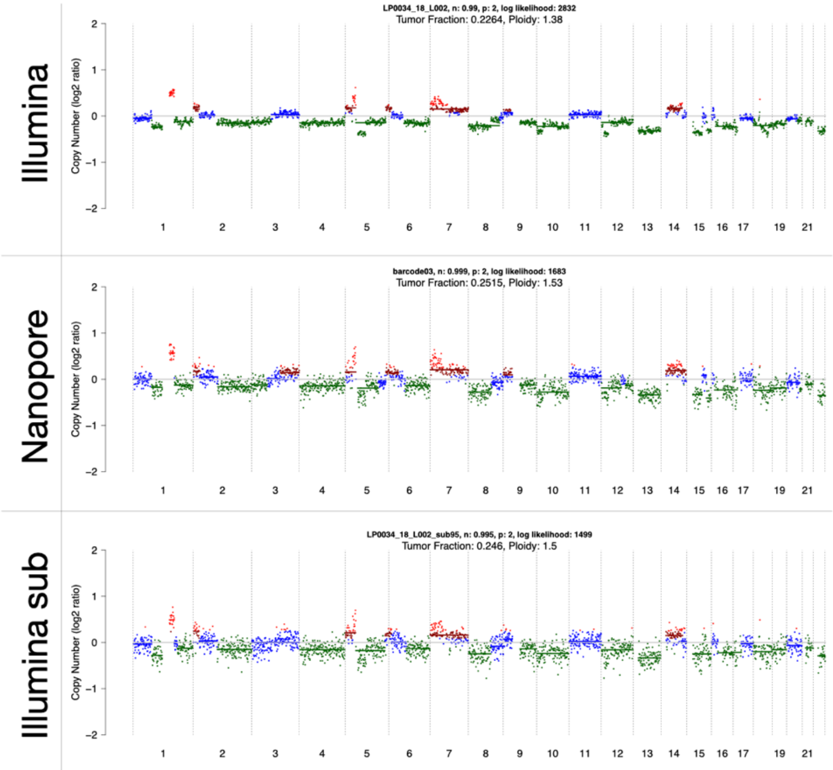

Patient A130E (BLCA_IV_003)

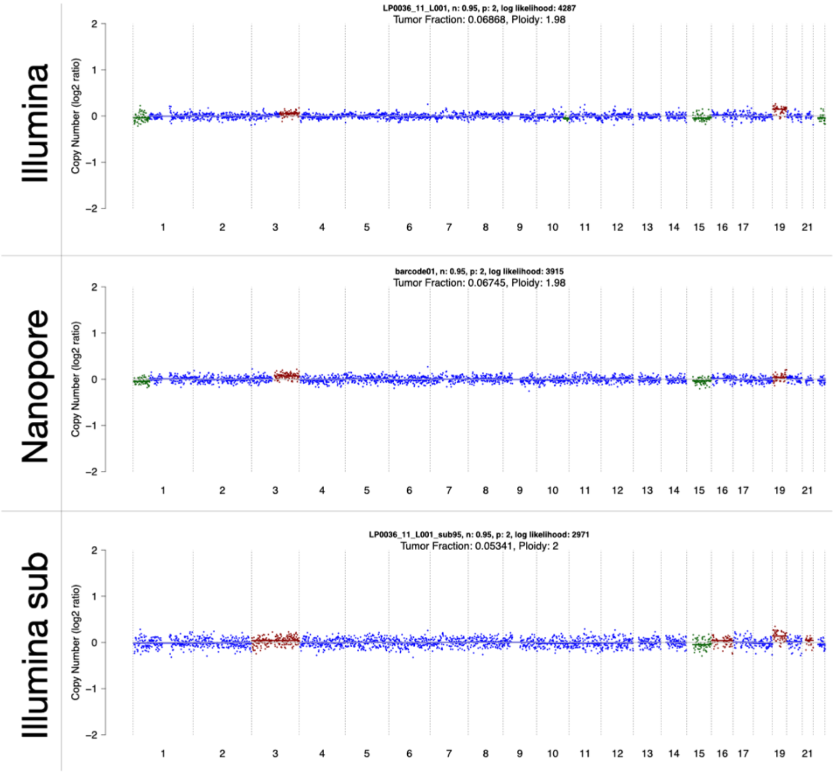

Patient A136E (BLCA_IV_004)

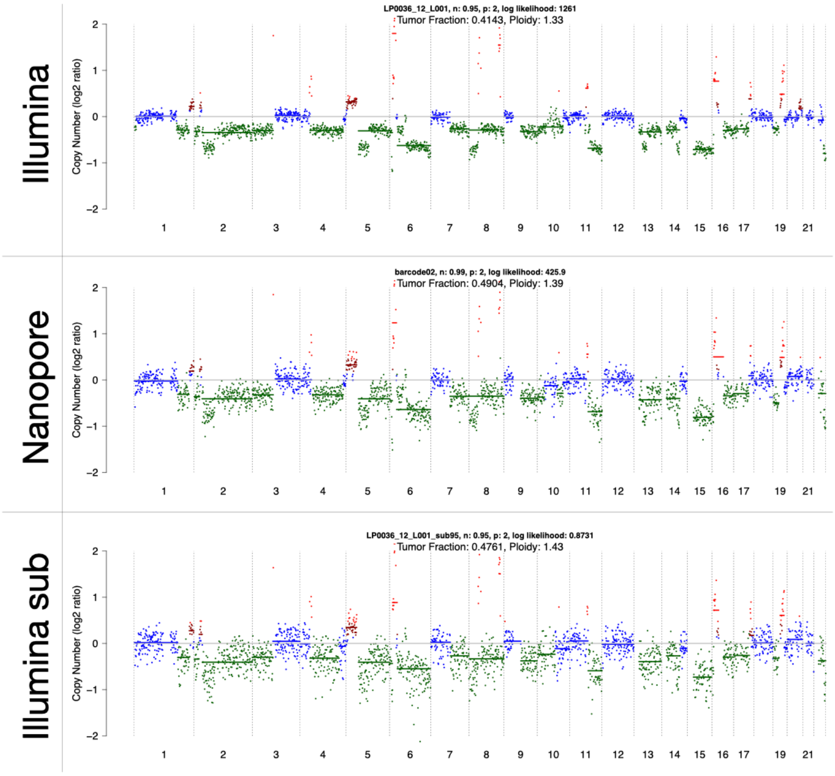

Patient A146E (BLCA_IV_006)

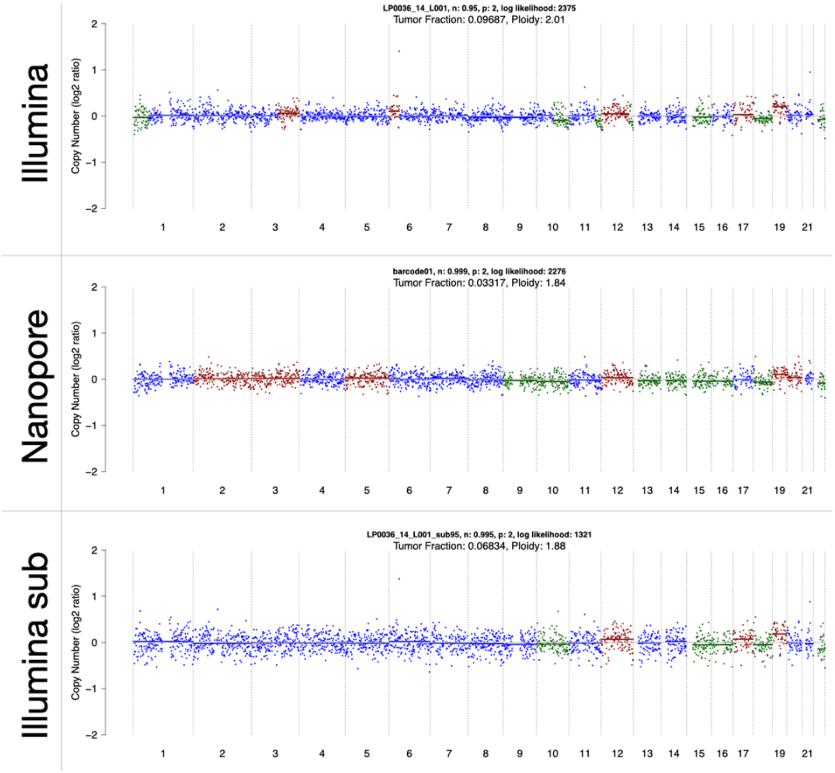

Patient A151E (BLCA_IV_001)

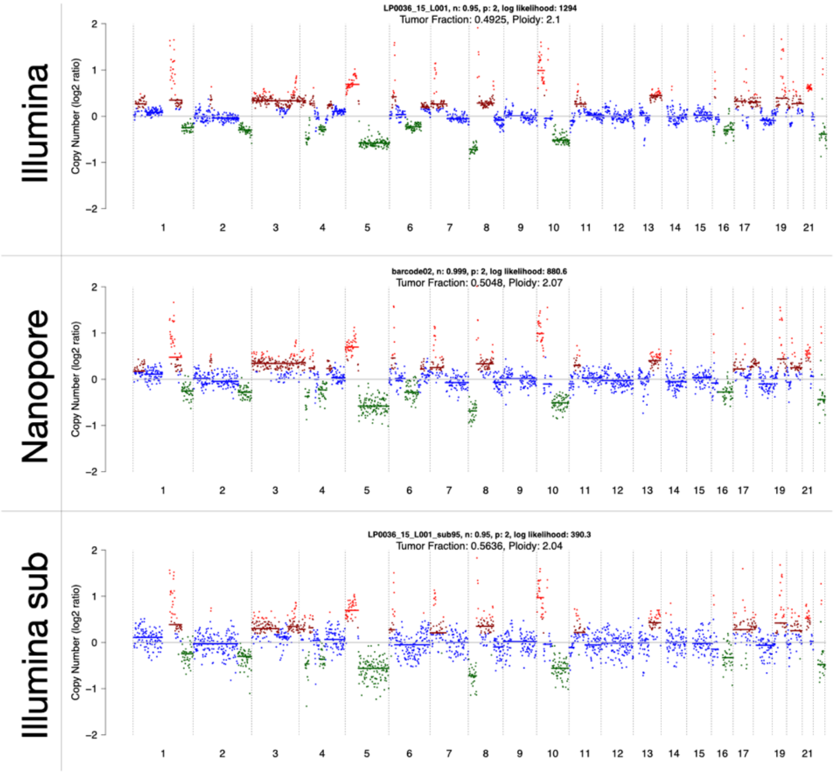

Patient A155E (BLCA_IV_007)

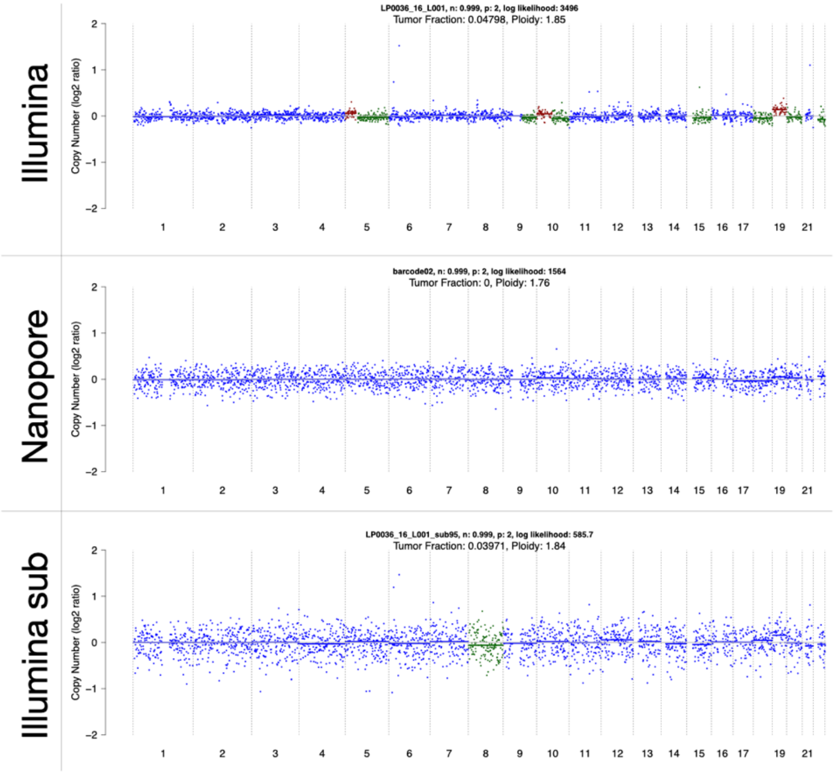

Patient A163E (BLCA_IV_008)

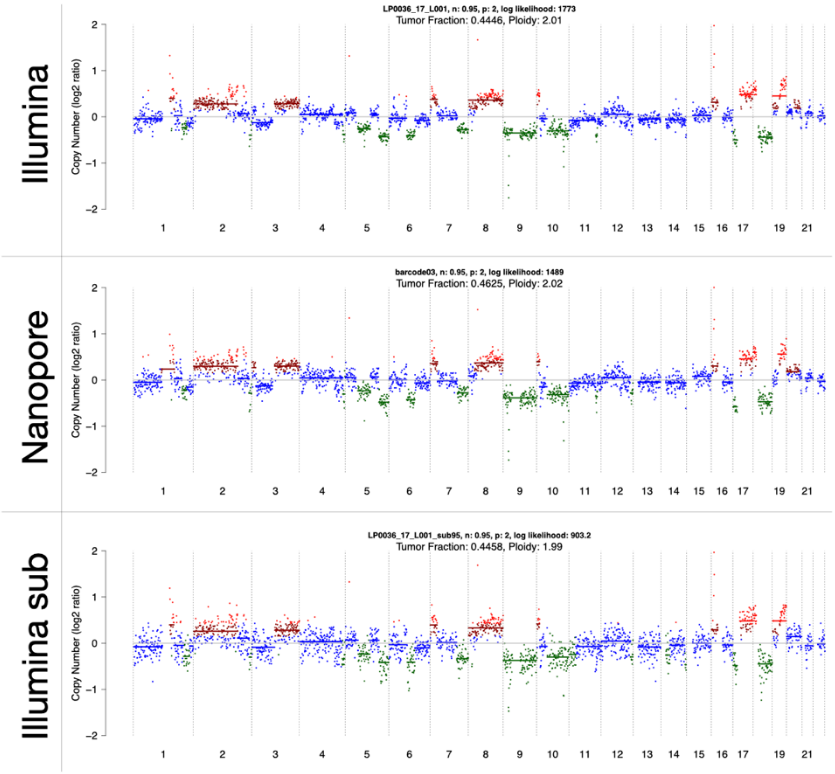

Patient D29 (BLCA_IV_002)

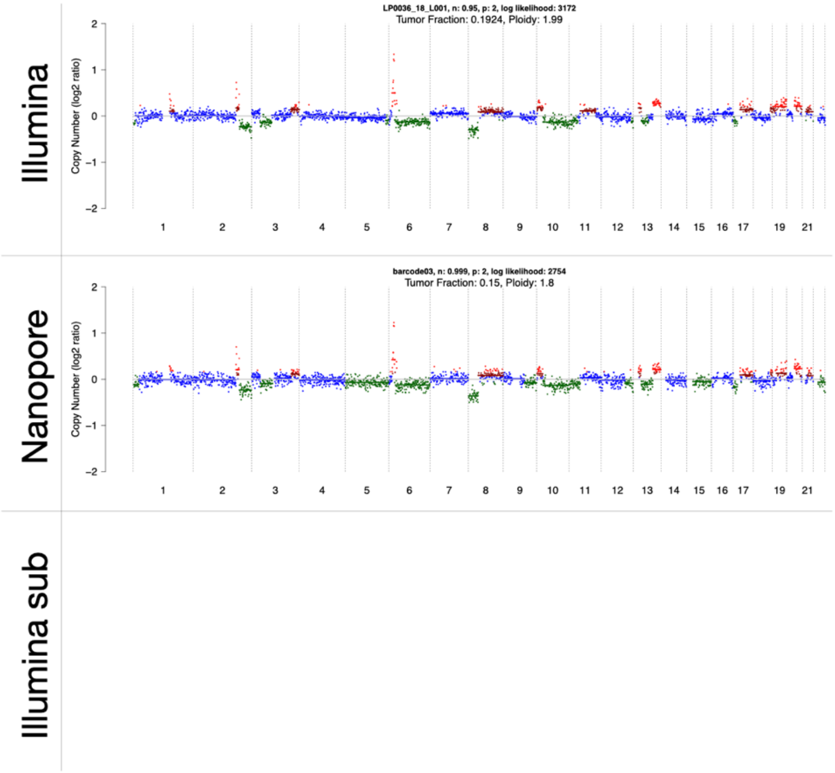

Patient D101 (BLCA_IV_005)

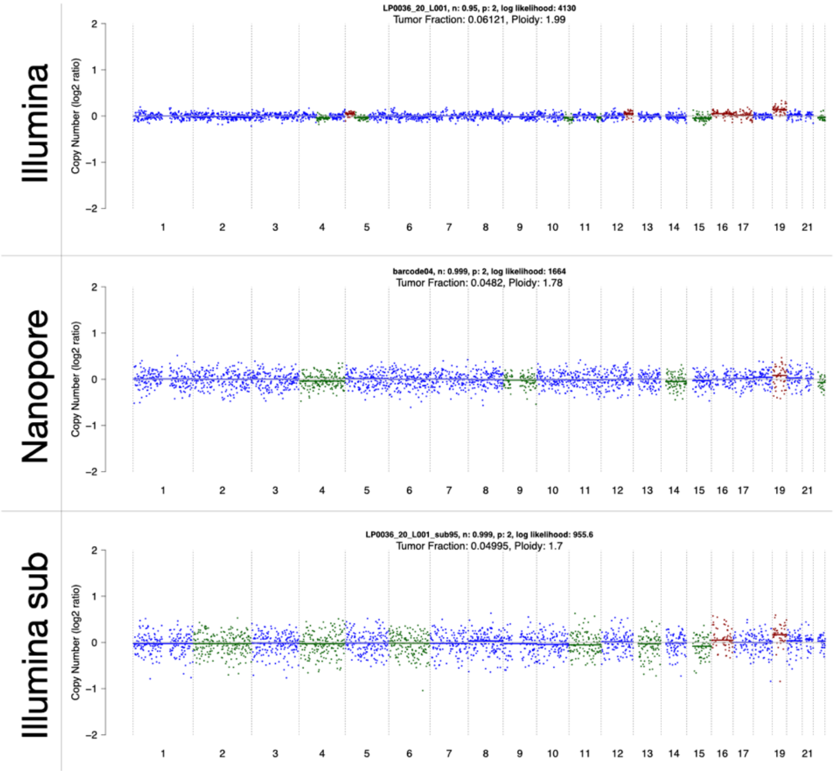

Patient N123 (CNTL_001)

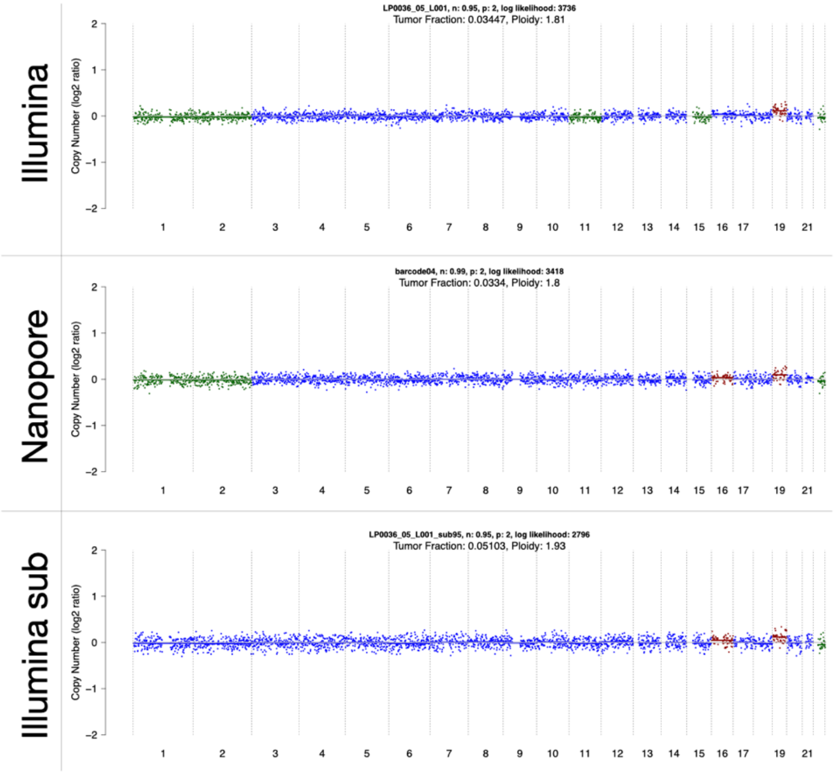

Patient N195E (CNTL_002)

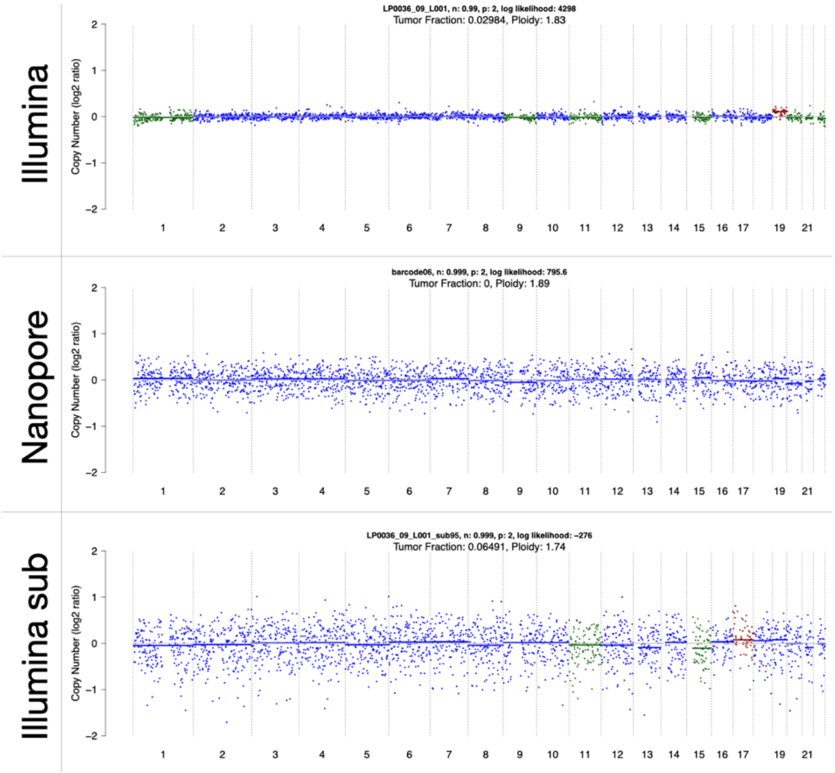

Appendix Figure S1. Copy number aberration profiles for the samples included in this study. For each sample the upper panel correspond to the data from the Illumina short-read sequencing, the middle panel to the ONT sequencing and the bottom panel to the Illumina sequencing down-sampled randomly to the same number of reads as the ONT data.

Appendix Figure S2.

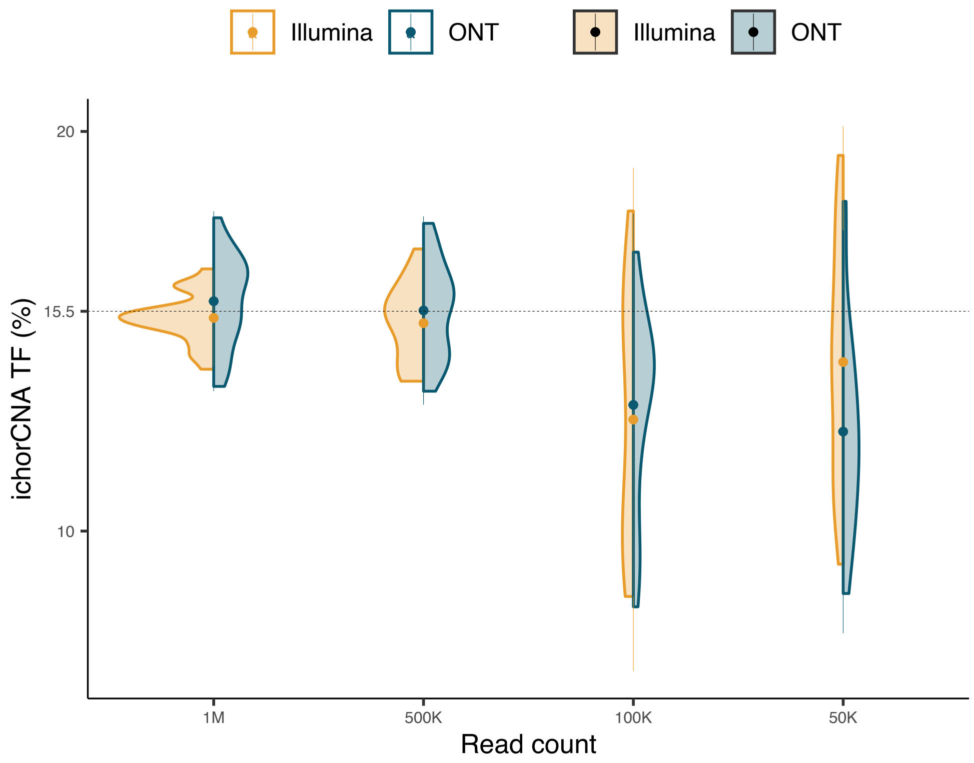

Appendix Figure S2. Accuracy of the tumor fraction (TF) calculation depending on the coverage. ichorCNA TF was calculated from in silico data admixture of know TF (see Methods) simulating 25 samples. Illumina data are shown in orange and Nanopore (ONT) data in blue.

Appendix Figure S3

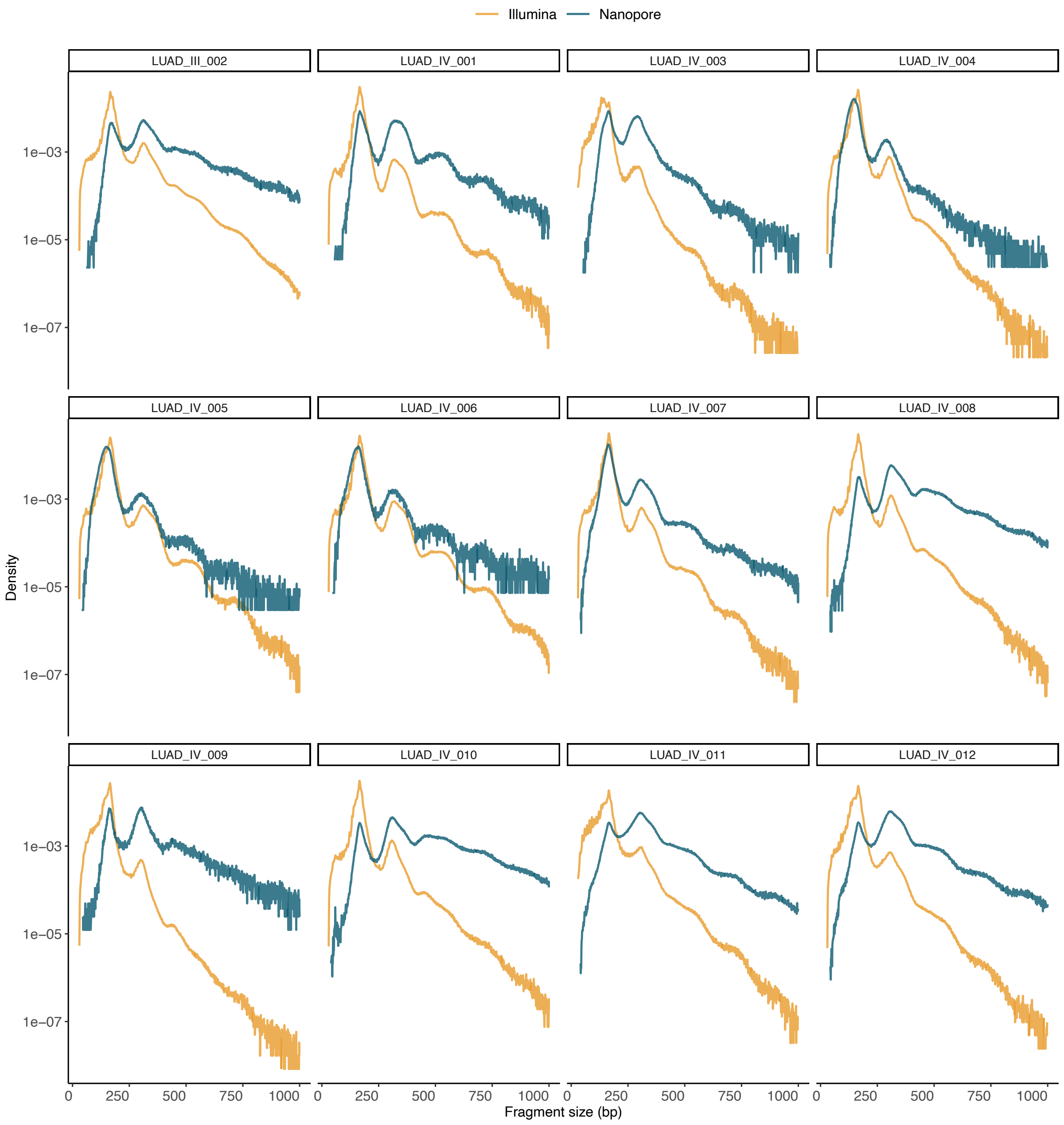

Appendix Figure S3. cfDNA fragment size distribution for the plasma samples included in this study. Color indicates the sequencing type (blue: Nanopore and orange: Illumina). Header numbers represent patient IDs.

Appendix Figure S4

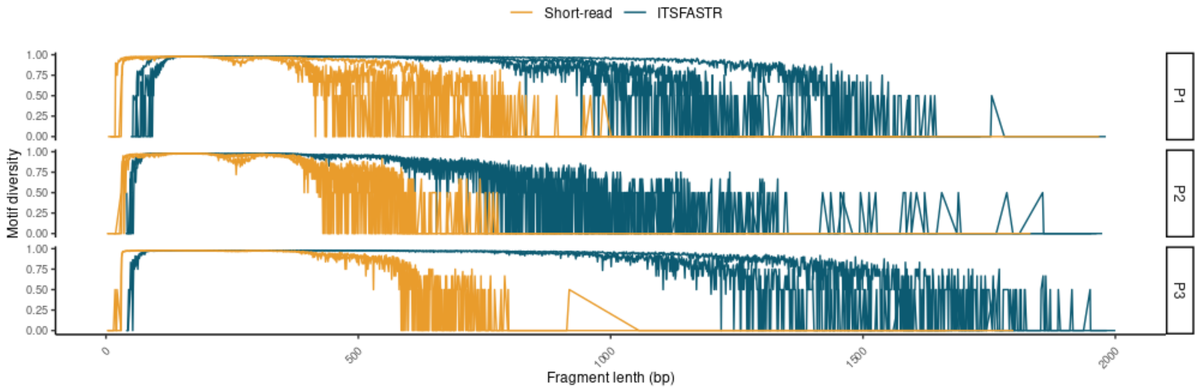

Appendix Figure S4. cfDNA fragment-end motif diversity calculated for each protocol tested in the study. Color indicates the sequencing type (blue: Nanopore and orange: Illumina).

Appendix Figure S5

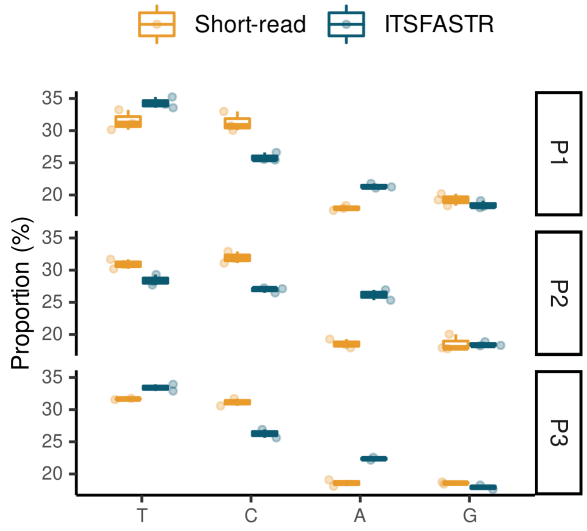

Appendix Figure S5. cfDNA fragment-end mononucleotide proportions calculated for each protocol tested in the study.

Appendix Figure S6.

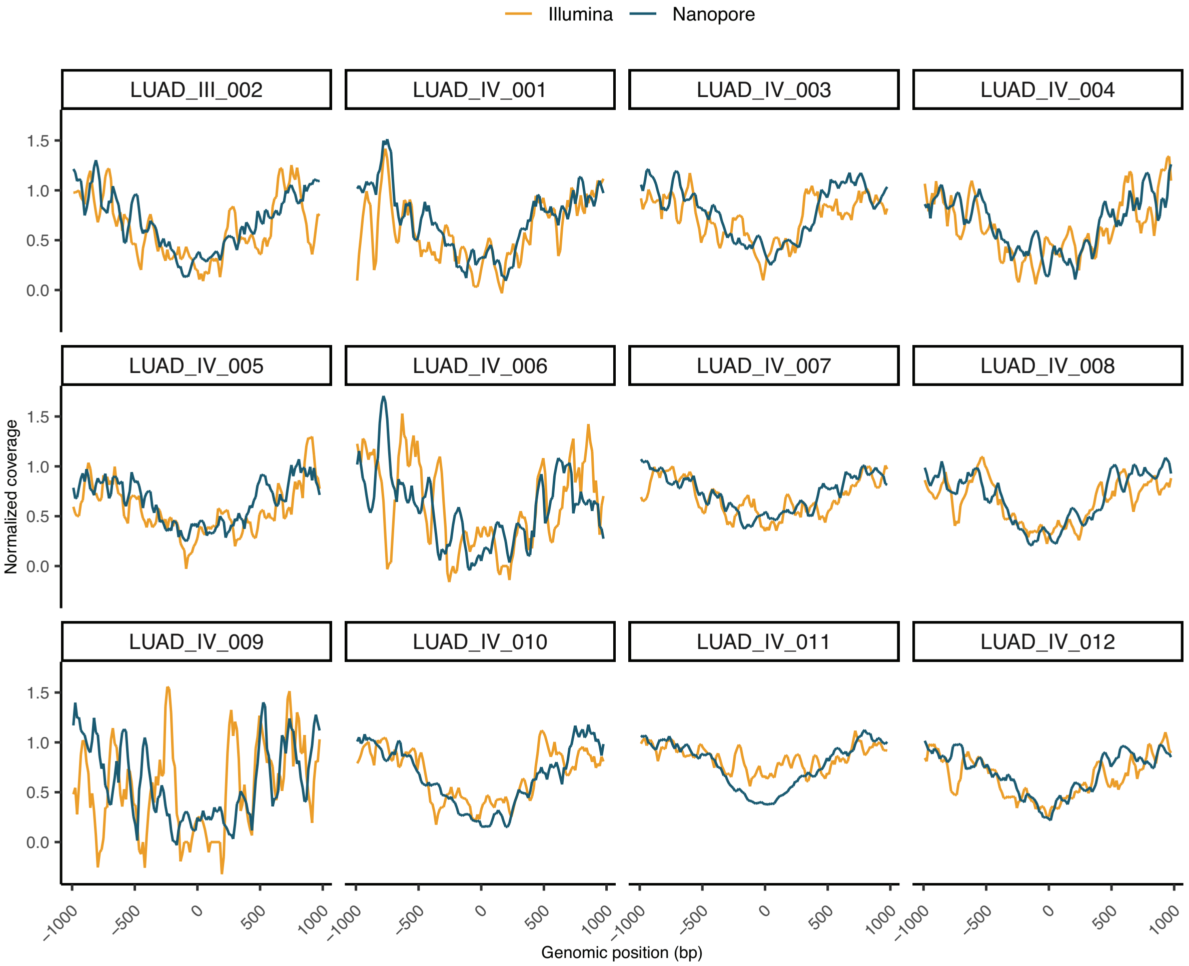

Appendix Figure S6. sequencing coverage near TSS regions of plasma samples.

Appendix Figure S7.

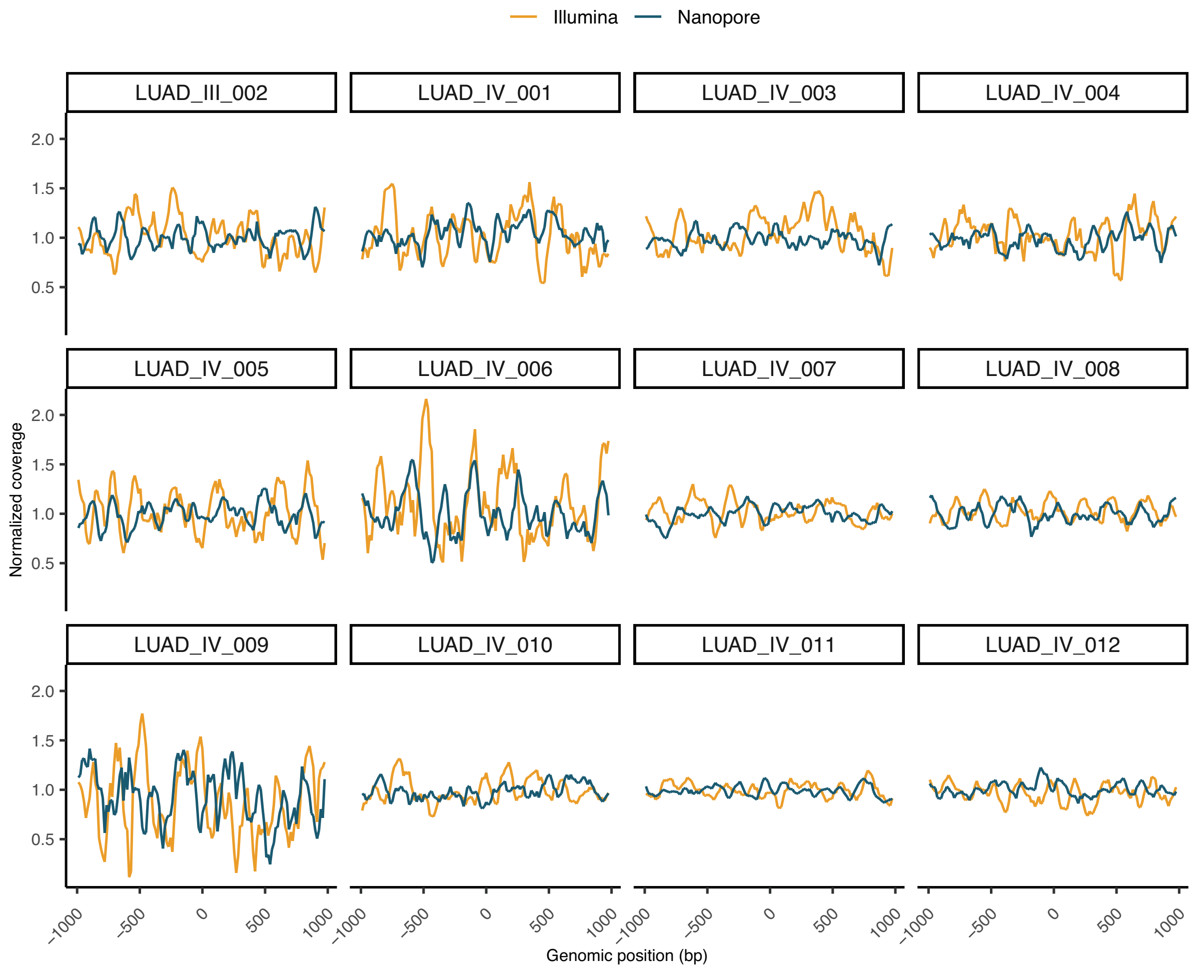

Appendix Figure S7. sequencing coverage near nucleosome-rich regions of plasma samples.

Appendix Figure S8.

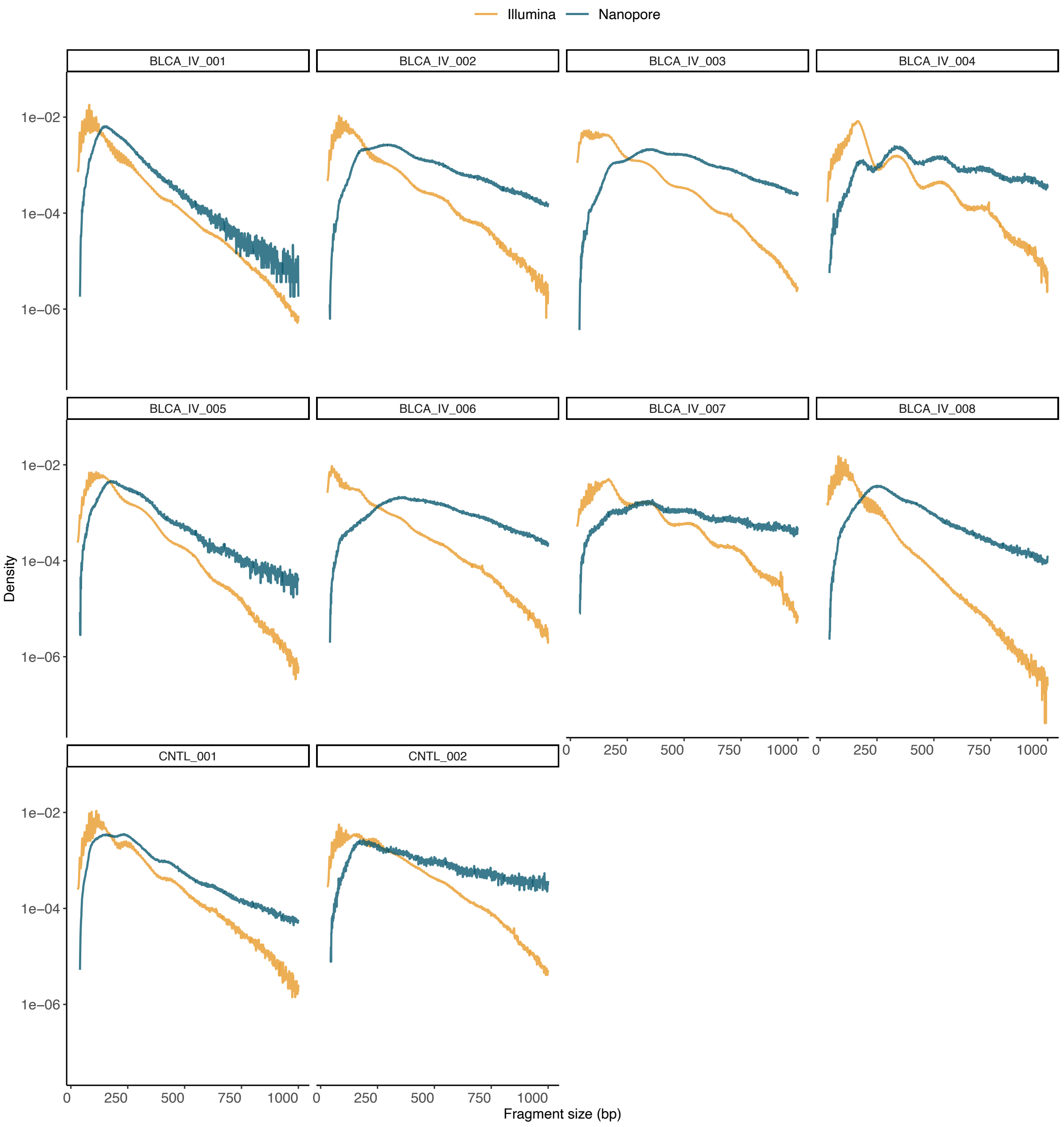

Appendix Figure S8. cfDNA fragment size distribution for the urine samples included in this study. Color indicates the sequencing type (blue: Nanopore and orange: Illumina). Header numbers represent patient IDs.

Appendix Figure S9.

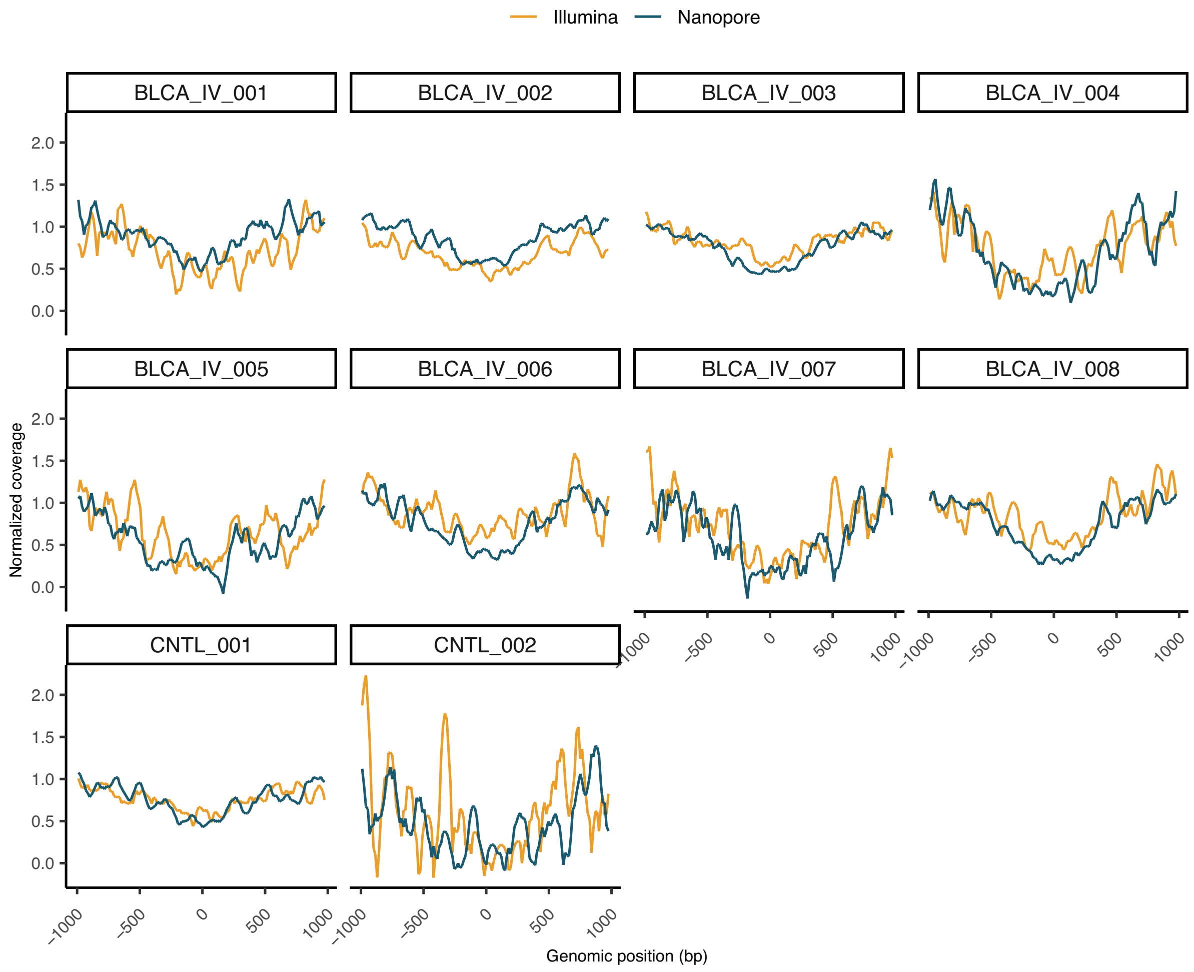

Appendix Figure S9. sequencing coverage near TSS regions of urine samples.

Appendix Figure S10.

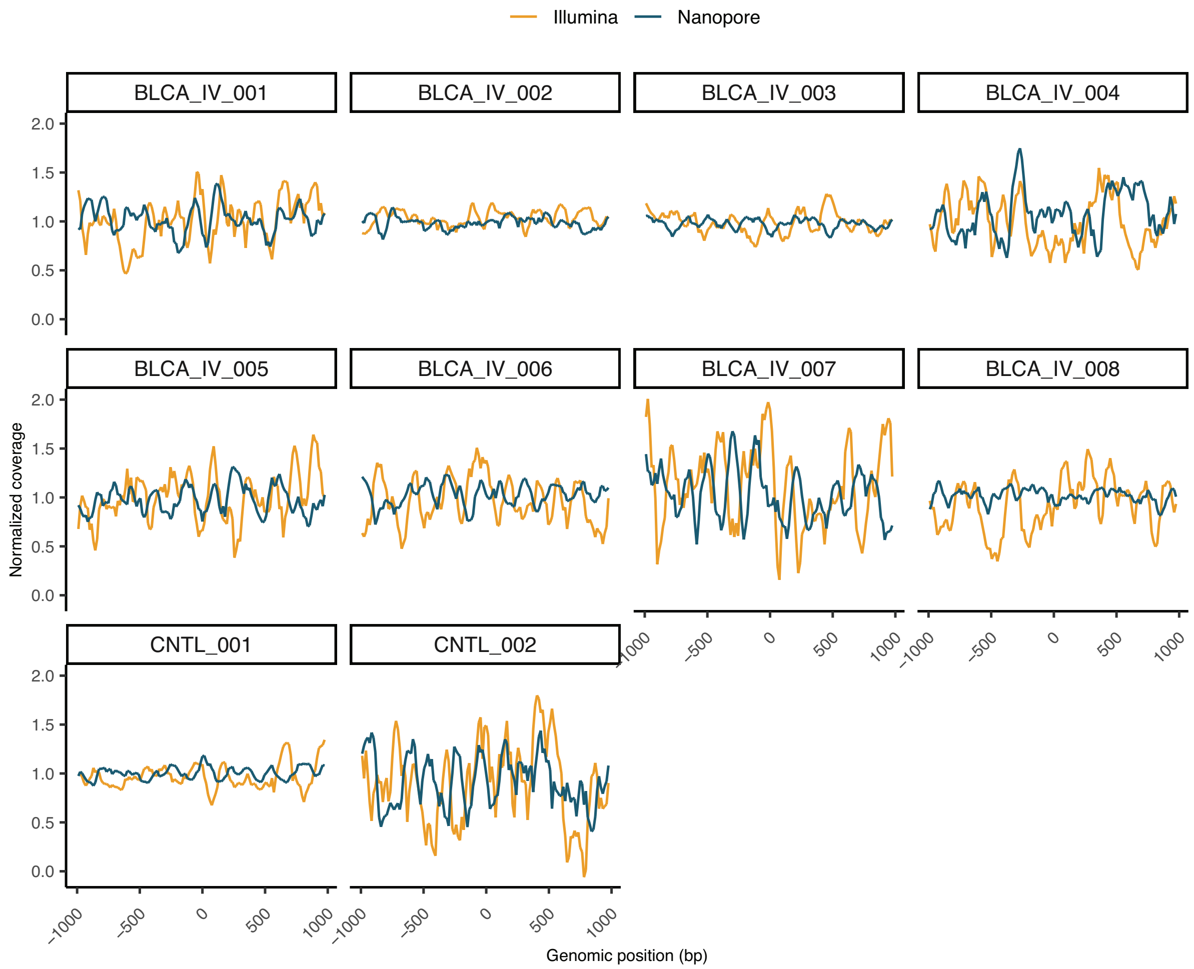

Appendix Figure S10. sequencing coverage near nucleosome-rich regions of urine samples.
