# Supplementary material for: Real‐time analysis of the cancer genome and fragmentome from plasma and urine cell‐free DNA using nanopore sequencing
Source: EMBO Mol Med. 2023 Nov 9;15(12):e17282. doi: 10.15252/emmm.202217282 (PMC10701599; doi:10.15252/emmm.202217282)
Supplement: Supplementary file 2 — Expanded View Figures PDF [file EMMM-15-e17282-s007.pdf]

Expanded View Figures

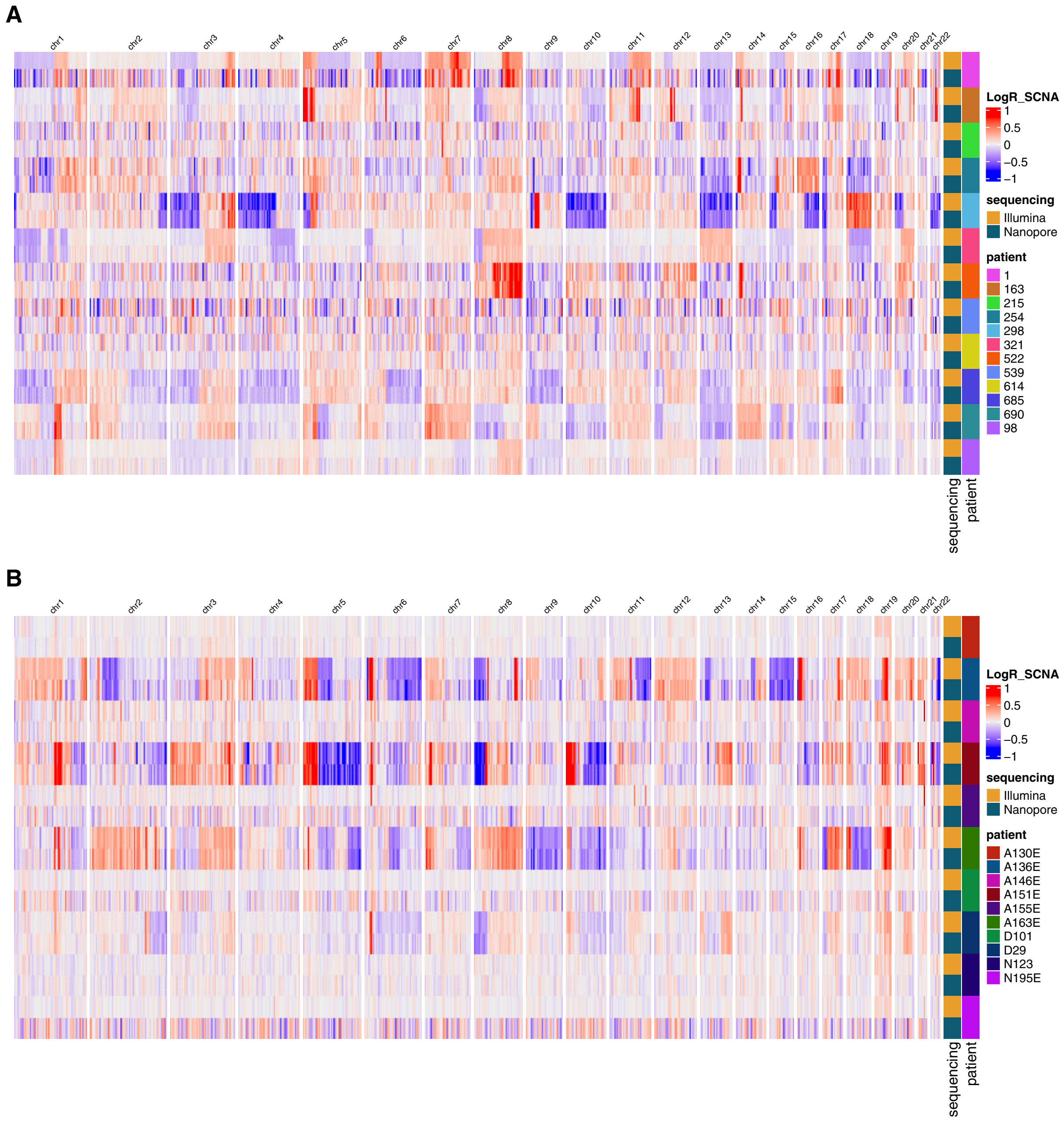

Figure EV1. Copy number aberration heatmaps for the samples included in this study.

A, B (A) Late-stage lung plasma samples and (B) urine samples. Illumina sequencing is downsampled randomly to the same number of reads as the nanopore data.

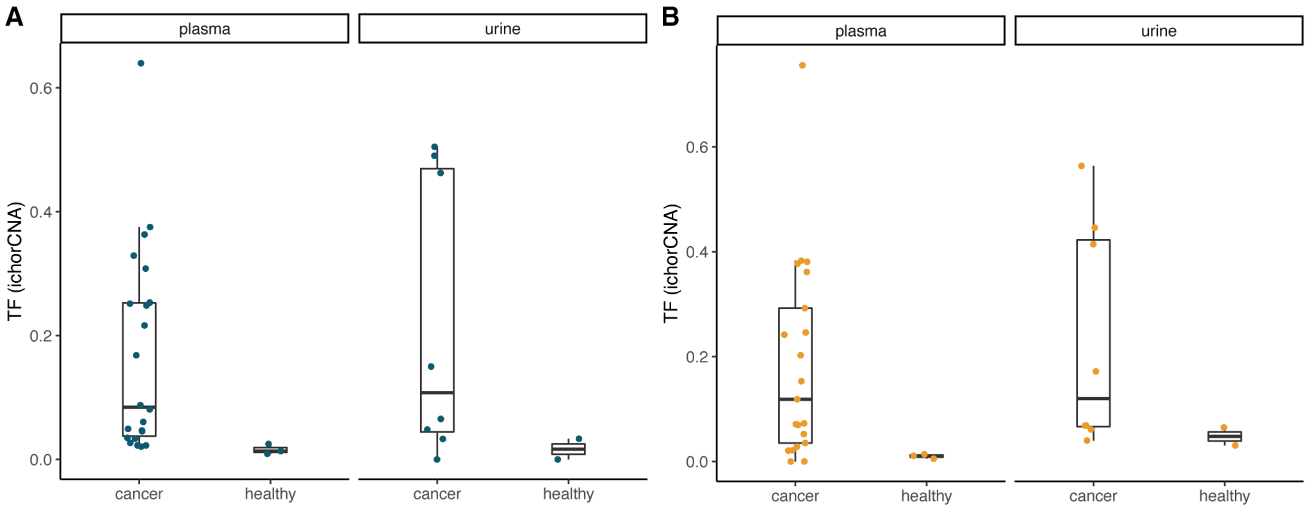

Figure EV2. Tumor fraction by ichorCNA for the cancer and healthy samples included in this study.

A, B (A) From the nanopore data and (B) from the Illumina data.

© 2023 The Authors
