# Supplementary material for: Real‐time analysis of the cancer genome and fragmentome from plasma and urine cell‐free DNA using nanopore sequencing
Source: EMBO Mol Med. 2023 Nov 9;15(12):e17282. doi: 10.15252/emmm.202217282 (PMC10701599; doi:10.15252/emmm.202217282)
Supplement: Supplementary file 8 [file EMMM-15-e17282-s004.pdf]

EMBO Molecular Medicine

Real-time analysis of the cancer genome and fragmentome from plasma and urine cell-free DNA using Nanopore sequencing.

Ymke van der Pol, Normastuti Adhini Tantyo, Nils Evander, Anouk Hentschel, Birgit Wever, Jip Ramaker, Sanne Bootsma, Marieke Fransen, Kristiaan Lenos, Louis Vermeulen, Famke Schneiders, Idris Bahce, Jakko Nieuwenhuijzen, Renske Steenbergen, D Michiel Pegtel, Norbert Moldovan, and Florent Mouliere
DOI: 10.15252/emmm.202217282

Corresponding author(s): Florent Mouliere (f.mouliere@amsterdamumc.nl) , Norbert Moldovan (n.moldovan@amsterdamumc.nl)

Review Timeline:

Submission Date:	11th Dec 22
Editorial Decision:	12th Jan 23
Revision Received:	7th Jun 23
Editorial Decision:	29th Jun 23
Authors' Correspondence:	3rd Jul 23
Editor's Correspondence:	18th Jul 23
Revision Received:	23rd Sep 23
Editorial Decision:	10th Oct 23
Revision Received:	13th Oct 23
Accepted:	17th Oct 23

Editor: Lise Roth

Transaction Report:

(Note: With the exception of the correction of typographical or spelling errors that could be a source of ambiguity, letters and reports are not edited. Depending on transfer agreements, referee reports obtained elsewhere may or may not be included in this compilation. Referee reports are anonymous unless the Referee chooses to sign their reports.)

12th Jan 2023

Dear Dr. Mouliere,

Thank you for the submission of your manuscript to EMBO Molecular Medicine. We have now received feedback from the three reviewers who agreed to evaluate your manuscript. As you will see from the reports below, the referees acknowledge the interest of the study and are overall supporting publication of your work pending appropriate revisions.

Addressing the reviewers' concerns in full will be necessary for further considering the manuscript in our journal, and acceptance of the manuscript will entail a second round of review. EMBO Molecular Medicine encourages a single round of revision only and therefore, acceptance or rejection of the manuscript will depend on the completeness of your responses included in the next, final version of the manuscript. For this reason, and to save you from any frustrations in the end, I would strongly advise against returning an incomplete revision.

If you would like to discuss further the points raised by the referees, I am available to do so via email or video. Let me know if you are interested in this option.

Revised manuscripts should be submitted within three months of a request for revision; they will otherwise be treated as new submissions, except under exceptional circumstances in which a short extension is obtained from the editor.

When submitting your revised manuscript, please carefully review the instructions that follow below. We perform an initial quality control of all revised manuscripts before re-review; failure to include requested items will delay the evaluation of your revision.

We require:

1) A .docx formatted version of the manuscript text (including legends for main figures, EV figures and tables). Please make sure that the changes are highlighted to be clearly visible.

2) Individual production quality figure files as .eps, .tif, .jpg (one file per figure). For guidance, download the 'Figure Guide PDF' (https://www.embopress.org/page/journal/17574684/authorguide#figureformat).

3) At EMBO Press we ask authors to provide source data for the main figures. Our source data coordinator will contact you to discuss which figure panels we would need source data for and will also provide you with helpful tips on how to upload and organize the files.

4) A .docx formatted letter INCLUDING the reviewers' reports and your detailed point-by-point responses to their comments. As part of the EMBO Press transparent editorial process, the point-by-point response is part of the Review Process File (RPF), which will be published alongside your paper.

5) A complete author checklist, which you can download from our author guidelines (https://www.embopress.org/page/journal/17574684/authorguide#submissionofrevisions). Please insert information in the checklist that is also reflected in the manuscript. The completed author checklist will also be part of the RPF.

6) Please note that all corresponding authors are required to supply an ORCID ID for their name upon submission of a revised manuscript. An ORCID identifier is currently missing for Norbert Moldovan.

7) It is mandatory to include a 'Data Availability' section after the Materials and Methods. Before submitting your revision, primary datasets produced in this study need to be deposited in an appropriate public database, and the accession numbers and database listed under 'Data Availability'. Please remember to provide a reviewer password if the datasets are not yet public (see https://www.embopress.org/page/journal/17574684/authorguide#dataavailability).

In case you have no data that requires deposition in a public database, please state so in this section. Note that the Data Availability Section is restricted to new primary data that are part of this study.

8) For data quantification: please specify the name of the statistical test used to generate error bars and P values, the number (n) of independent experiments (specify technical or biological replicates) underlying each data point and the test used to calculate p-values in each figure legend. The figure legends should contain a basic description of n, P and the test applied. Graphs must include a description of the bars and the error bars (s.d., s.e.m.). Please provide exact p values.

9) Our journal encourages inclusion of *data citations in the reference list* to directly cite datasets that were re-used and obtained from public databases. Data citations in the article text are distinct from normal bibliographical citations and should

directly link to the database records from which the data can be accessed. In the main text, data citations are formatted as follows: "Data ref: Smith et al, 2001" or "Data ref: NCBI Sequence Read Archive PRJNA342805, 2017". In the Reference list, data citations must be labeled with "[DATASET]". A data reference must provide the database name, accession number/identifiers and a resolvable link to the landing page from which the data can be accessed at the end of the reference. Further instructions are available at .

10) We replaced Supplementary Information with Expanded View (EV) Figures and Tables that are collapsible/expandable online. A maximum of 5 EV Figures can be typeset. EV Figures should be cited as 'Figure EV1, Figure EV2" etc... in the text and their respective legends should be included in the main text after the legends of regular figures.
- For the figures that you do NOT wish to display as Expanded View figures, they should be bundled together with their legends in a single PDF file called *Appendix*, which should start with a short Table of Content. Appendix figures should be referred to in the main text as: "Appendix Figure S1, Appendix Figure S2" etc.
- Additional Tables/Datasets should be labeled and referred to as Table EV1, Dataset EV1, etc. Legends have to be provided in a separate tab in case of .xls files. Alternatively, the legend can be supplied as a separate text file (README) and zipped together with the Table/Dataset file.
See detailed instructions here:

11) The paper explained: EMBO Molecular Medicine articles are accompanied by a summary of the articles to emphasize the major findings in the paper and their medical implications for the non-specialist reader. Please provide a draft summary of your article highlighting
- the medical issue you are addressing,
- the results obtained and
- their clinical impact.
This may be edited to ensure that readers understand the significance and context of the research. Please refer to any of our published articles for an example.

12) For more information: There is space at the end of each article to list relevant web links for further consultation by our readers. Could you identify some relevant ones and provide such information as well? Some examples are patient associations, relevant databases, OMIM/proteins/genes links, author's websites, etc...

13) Author contributions: CRediT has replaced the traditional author contributions section because it offers a systematic machine readable author contributions format that allows for more effective research assessment. Please remove the Authors Contributions from the manuscript and use the free text boxes beneath each contributing author's name in our system to add specific details on the author's contribution. More information is available in our guide to authors.
14) Disclosure statement and competing interests: We updated our journal's competing interests policy in January 2022 and request authors to consider both actual and perceived competing interests. Please review the policy https://www.embopress.org/competing-interests and update your competing interests if necessary.

15) Every published paper now includes a 'Synopsis' to further enhance discoverability. Synopses are displayed on the journal webpage and are freely accessible to all readers. They include a short stand first (maximum of 300 characters, including space) as well as 2-5 one-sentences bullet points that summarizes the paper. Please write the bullet points to summarize the key NEW findings. They should be designed to be complementary to the abstract - i.e. not repeat the same text. We encourage inclusion of key acronyms and quantitative information (maximum of 30 words / bullet point). Please use the passive voice. Please attach these in a separate file or send them by email, we will incorporate them accordingly.

Please also suggest a striking image or visual abstract to illustrate your article as a PNG file 550 px wide x 300-600 px high.

16) As part of the EMBO Publications transparent editorial process initiative (see our Editorial at http://embomolmed.embopress.org/content/2/9/329), EMBO Molecular Medicine will publish online a Review Process File (RPF) to accompany accepted manuscripts.
In the event of acceptance, this file will be published in conjunction with your paper and will include the anonymous referee reports, your point-by-point response and all pertinent correspondence relating to the manuscript. Let us know whether you agree with the publication of the RPF and as here, if you want to remove or not any figures from it prior to publication.
Please note that the Authors checklist will be published at the end of the RPF.

EMBO Molecular Medicine has a "scooping protection" policy, whereby similar findings that are published by others during review or revision are not a criterion for rejection. Should you decide to submit a revised version, I do ask that you get in touch after three months if you have not completed it, to update us on the status.

I look forward to receiving your revised manuscript.

Yours sincerely,

Lise Roth

Lise Roth, PhD
Senior Editor
EMBO Molecular Medicine

Please note: When submitting your revision you will be prompted to enter your funding and payment information. This will allow Wiley to send you a quote for the article processing charge (APC) in case of acceptance. This quote takes into account any reduction or fee waivers that you may be eligible for. Authors do not need to pay any fees before their manuscript is accepted and transferred to the publisher.

EMBO Press participates in many Publish and Read agreements that allow authors to publish Open Access with reduced/no publication charges. Check your eligibility: https://authorservices.wiley.com/author-resources/Journal-Authors/open-access/affiliation-policies-payments/index.html

***** Reviewer's comments *****

Referee #2 (Comments on Novelty/Model System for Author):

Technical quality could not fully be assessed because the github code archive was not available. The rest seemed fine.

Novelty: See major issue #1 below.

Impact: Overall, the approach seems promising, but clear data for tumor detection accuracy are missing.

Referee #2 (Remarks for Author):

SUMMARY:

The authors show that Nanopore sequencing can be used for the analysis of copy number abberations and coverage-based fragmentomic analyses of cell-free DNA. They demonstrate that with an optimized laboratory and computational workflow, Nanopore sequencing can provide useful genomic and fragmentomic data within 7 hours of sample collection.

Based on the tumor content inferred from copy number aberrations, the authors argue that Nanopore sequencing leads to similar results as short read sequencing, when comparing the two technologies at the same sequencing depth. They also show that the two technologies generally agree well in terms of which alterations are detected. The coverage at transcription start sites and quiescent sites dereived from Nanopore reads behaves similar to the coverage obtained from short-read sequencing, indicating that Nanopore data could be amendable for fragmentomics analysis, e.g. for tumor detection or tissue deconvolution, in the future.

Comparisons of tumor detection accuracy (sensitivity, specificity, ROC-AUC etc) with Nanopore vs conventional short-read sequencing are not provided, but would be useful in order to assess how reliable the proposed method is. However, reliable accuracy comparisons would be hard to obtain given the limited sample size of this study.

The authors show that Nanopore sequencing can recover more long fragments (>300 bp) than conventional methods, and they show that these long fragments are also partially derived from tumor cells. They argue that these long fragments could be useful for tumor detection, but do not provide any further details. It would be nice to see how these longer fragments can be

Overall, the study demonstrates the potential of Nanopore sequencing of cfDNA, especially due to the fast turnaround time, and shows that this is a promising research direction, but it doesn't give very concrete performance measures of this technology.

MAJOR POINTS:

1. Novelty
########

Similar, published studies include:

Martignano et al.: "Nanopore sequencing from liquid biopsy: analysis of copy number variations from cell-free DNA of lung cancer patients" https://molecular-cancer.biomedcentral.com/articles/10.1186/s12943-021-01327-5
Here, the authors already show that Illumina and Nanopore results for CNV analysis is highly concordant (for a cohort of 10 lung cancer patients). They also mention the fast turnaround time of the technology ("According to our sequencing statistics, 2 M reads are produced in less than 3 h. This means that the entire workflow -from blood withdrawal to bioinformatic analyses- can be performed in less than a working day").

Katsman et al.: "Detecting cell-of-origin and cancer-specific methylation features of cell-free DNA from Nanopore sequencing" https://genomebiology.biomedcentral.com/articles/10.1186/s13059-022-02710-1
The autors report results for ONT sequencing of plasma from 7 healthy controls and 6 lung cancer patients. They show correlations between Illumina and ONT-based tumor fractions estimated by ichorCNA, similar as in Figure 1C of this study (however only based on 4 samples)
Furthermore, Katsman et al. show that nucleosome positioning is preserved in ONT-derived data, using CTCF binding motifs. They also compared fragment length profiles and end motifs between Illumina and ONT-derived data. Interestingly, Katsman et al. don't report longer fragment lenghts in ONT data compared to Illumina data.
The Katsman et al. study is cited, but not properly discussed in this manuscript.

Overall, given these two studies, the novelty of this manuscript is limited (and not properly discussed). Some of the most interesting aspects are the application to urine samples and, potentially, the ITSFASTR method (which is, however, not described in detail, and the github repository is not accessible).

2. Sample size / Tumor detection accuracy
#############################

As mentioned above, while the high correlation values to Illumina-derived data is promising, comparisons of tumor detection accuracy (sensitivity, specificity, ROC-AUC etc) with Nanopore vs conventional short-read sequencing are not provided. Those would be useful to assess how reliable the proposed method is. However, reliable comparisons would be hard to obtain given the limited sample size of this study.

3. ITSFASTR
#########
The ITSFASTR method could be interesting, but is not described in detail, and the github repository is not accessible. I therefore could not assess the quality and utility of this tool.

MINOR POINTS:
Results, page 2:
• Rather report median read numbers per sample plus average coverage, instead of the total number of reads across all samples.
• The down-sampling of Illumina reads should be mentioned also here in the main text, as this is an important aspect and has strong implications. Also, how many reads can Illumina vs Nanopore yield for comparable costs? One could down-sample also to a ratio of reads that represents similar costs.
• The 2 samples for which the Nanopore-derived tumor fraction is zero, but the Illumina derived tumor fraction is above zero should be discussed. Also, there seems to be an amplification artifact that is common between the two control samples.
• The limitations of CNV calls from lower read depths compared to full-read-depth Illumina data should be discussed.

Results, page 3:
• The processing time of ~ 7h with a GPU could be indicated in the figure. A similar comparison (laptop / more advanced hardware) could then also be shown for the Illumina approach (probably less impact there?)
• Cite also the original study for this sentence: "Canonically, cfDNA in blood plasma has been described as short, fragmented molecules centered around 167 bp (and multiple) due to enzymatic cleavage linked to cell-death"
• A version of the fragment size plots with a linear scale on the y axis would be nice. Otherwise, the total fraction of reads within certain proportions is hard to assess intuitively.
• "The diversity of the trinucleotides at the end of these long cfDNA fragments, determined using Gini index, was not different to the one in shorter (< 300bp) size ranges": Later on, the authors state that fragments with lengths <100 bp have decreased diversity, so "(<300bp)" must be inaccurate. Maybe 100-300bp is more accurate?
• Figure 2B: The power for this analysis is low, as the number of sample is low and also no significant change between fragments of sizes <150 bp and 150-300 bp could be observed. Higher tumor fractions are expected in the <150 bp compared to

150-300 bp based on previous work, at least for Illumina data (Mouliere, Science Translational Medicine 2018). Can such an effect be observed for the collected Illumina data? In the aforementioned study, the authors used the t-MAD score to assess enrichment for tumor-derived fragments among short fragments. Would this be appropriate here as well?

Results, page 4:
• Suppl. Fig. 6-7: The sample names shown in these panels are different then for the CNA plots. Also, it would be easier to compare profiles between ONT and Illumina if the profiles were shown in a single plot per patient (ONT in blue, Illumina in yellow).

Discussion, page 4:
• "This has to be mitigated by the relative higher cost / Gb of the MinION data." This should be discussed in more detail. What are the costs / Gb? This comes back to my comments about downsampling.

Discussion, page 5:
• "Moreover, we observed that these long fragments contain tumor-derived ctDNA signal, which could be critical to recover in clinical conditions when such tumor signal is scarce (e.g. minimal residual disease detection)."Arguably sequencing deeper could be more important for MRD detection. Could the authors comment on that?
• Typo in: "minutes amount"

Methods, page 7:
• "CollectInsertSizeMetrics with default parameters": This could be problematic, as CollectInsertSizeMetrics automatically truncates the histogram tail. Please ensure that fragments with sizes below 1000bp or 2000bp are not truncated. While this likely will not lead to a major change in the conclusions, if distributions were truncated, update the fragment size comparisons to ONT accordingly.

Figure 1:
• A: CNA plots in panel A should be a separate panel. Also, please mark which plot comes from which technology more clearly.
• B: Spell out "ratio" in the axis labels
• C: "TF" is not explained in the figure legend.
• D: This could be shown in a stacked bar plot to provide information of the duration of sample preparation phases and computation phases separately.

Figure 2:
• C: Is this for the downsampled or full-scale Illumina data?

Referee #3 (Comments on Novelty/Model System for Author):

Novelty: This work represents an emerging field of ONT-based cell-free DNA assays. While prior work in the field has been done before, this work directly compares Illumina to Nanopore and represents an important advance in the field.
Medical impact: There is interest in this type of assay to be applied in personalized medicine. Importantly, ONT MinION machines present an opportunity for rapid analysis of patient samples with fast turnaround time and (potentially) affordable cost. However, the authors here only present theoretical turnaround times of the ITSFASTR assay, and do not process patient samples as they are collected.

Referee #3 (Remarks for Author):

In this manuscript, van der Pol, Tantyo et al have developed a technical workflow to analyze copy number alterations from cell-free DNA sequenced via the Oxford Nanopore MinON machine.

The manuscript is well written, has strong merit and is an important contribution to the field. While certain aspects of the manuscript require revision and development, I highly recommend this manuscript for eventual publication at EMM. There is a clinical need for fast, affordable cancer screening that might not be addressable with current ultra-high-throughput methods. While sequencing using high throughput (ex NovaSeq) sequencers is eventually more cost effective, a certain number of samples need to be sequenced at the same time, thus bottlenecking the diagnostic process. ONT sequencing and the ITSFASTR pipeline offer a solution to this bottleneck.

My comments:

1- Sample labels are uninformative. Cancer type and stage should be readily readable for each subplot.
2- Data for cancer-free controls is not used in any analysis, and should be used to at least compare cell-free DNA features (Tumor fraction, NDR, fragment lengths, and all other features looked at).
3- What portion of the Illumina workflow is more technically challenging, compared to ONT? The Illumina MiniSeq machine involves library preparation, dilution of libraries, and pipetting that diluted library in the Illumina cartridge. Is the ONT machine

significantly simpler? Why would the Illumina MiniSeq machine require "assuming immediate availability of all required machines and staff to operate them". How would an ONT MinON be used?
4- Since the authors did not use ITSFASTR in an actual point-of-care setting this should not be mentioned.
5- Mean and standard deviation (per sample) should be included in all sequencing metrics.
6- "Similar small and large genomic events" is qualitative. What is short? What is large?
7- The normalized coverage plots are interesting but do not provide a quantitative comparison between Short-read tech and nanopore. I would recommend some correlation metrics between the two sequencing modalities.
8- Can the authors expand on the relevance of sequencing longer cfDNA fragments via ONT if the tumor fractions do not change depending on the length (F2B).

Referee #4 (Comments on Novelty/Model System for Author):

In this study, the authors perform and interesting comparison between important technologies in an adequate manner. However, it is already known that the ONT technologies are capable of obtaining information from long-size DNA. Nonetheless, is interesting to see its application in DNA from plasma and urine. The inmediate clinical impact is not high since no mutations are being identify from liquid biopsy and the clinical impact of fragmentomics data is still unknown.

Referee #4 (Remarks for Author):

In this study, the authors include a series of plasma and urine samples from cancer patients to demonstrate that the ONT platforms are able to provide similar genomic profiles than conventional high-throughput platforms. The data shown herein is interesting for the liquid biopsy field applied to cancer and demonstrate that these easy-to-use and cheap platforms can provide important information from the tumor in a cost-efficient way.
I miss line numbering in the document. Please, see below my comments:

Minor comments:

- Abstract: The authors refer to lung patients in singular.

Major comments:

1. The authors should include a table with the patients´ clinicopatological characteristics. If they were included, the Supplementary Tables are not available to download.
2. Overall, the authors should include information and graphs from adequate controls:
- Germline DNA: The authors should sequence the germline DNA from the patients, at least with the ONT platform. It is important to visualize patterns from potential contaminant germline DNA into plasma and urine samples.
- Non-cancer controls: I also miss representations from the analysis of the sequencing data from non-cancer controls. They should be in supplementary materials. On top of that, the authors should sequence, at least with the ONT instrument, a number of plasma samples from unrelated healthy individuals.
3. It would be interesting to sequence the corresponding tumors and compare the genomic profiles with the ones from liquid biopsy samples. Is it possible to identify tumor structural variations, single nucleotide variants or insertions and deletions using liquid biopsy?

EMM-2022-17282

Response to Reviewer Comments

We thank both reviewers for their insightful comments regarding our manuscript (EMM-2022-17282). We have addressed all concerns raised by the reviewers and made additions to the manuscript for their suggested improvements and we provide a point-by-point description to these changes below. We believe that the reviewers' comments and the new additions have greatly strengthened this work and that this is clearly reflected in the revised manuscript.

******* Reviewer's comments *******

Referee #2 (Comments on Novelty/Model System for Author):

Technical quality could not fully be assessed because the github code archive was not available. The rest seemed fine.

Novelty: See major issue #1 below.

Impact: Overall, the approach seems promising, but clear data for tumor detection accuracy are missing.

Referee #2 (Remarks for Author):

SUMMARY:

The authors show that Nanopore sequencing can be used for the analysis of copy number abberations and coverage-based fragmentomic analyses of cell-free DNA. They demonstrate that with an optimized laboratory and computational workflow, Nanopore sequencing can provide useful genomic and fragmentomic data within 7 hours of sample collection.

Based on the tumor content inferred from copy number aberrations, the authors argue that Nanopore sequencing leads to similar results as short read sequencing, when comparing the two technologies at the same sequencing depth. They also show that the two technologies generally agree well in terms of which alterations are detected. The coverage at transcription start sites and quiescent sites derived from Nanopore reads behaves similar to the coverage obtained from short-read sequencing, indicating that Nanopore data could be amendable for fragmentomics analysis, e.g. for tumor detection or tissue deconvolution, in the future.

Comparisons of tumor detection accuracy (sensitivity, specificity, ROC-AUC etc) with Nanopore vs conventional short-read sequencing are not provided, but would be useful in order to assess how reliable the proposed method is. However, reliable accuracy comparisons would be hard to obtain given the limited sample size of this study.

The authors show that Nanopore sequencing can recover more long fragments (>300 bp) than conventional methods, and they show that these long fragments are also partially derived from tumor cells. They argue that these long fragments could be useful for tumor detection, but do not provide any further details. It would be nice to see how these longer fragments can be

Overall, the study demonstrates the potential of Nanopore sequencing of cfDNA, especially due to the fast turnaround time, and shows that this is a promising research direction, but it doesn't give very concrete performance measures of this technology.

MAJOR POINTS:

1. Novelty
########

Similar, published studies include:

Martignano et al.: "Nanopore sequencing from liquid biopsy: analysis of copy number variations from cell-free DNA of lung cancer patients" https://molecular-cancer.biomedcentral.com/articles/10.1186/s12943-021-01327-5
Here, the authors already show that Illumina and Nanopore results for CNV analysis is highly concordant (for a cohort of 10 lung cancer patients). They also mention the fast turnaround time of the technology ("According to our sequencing statistics, 2 M reads are produced in less than 3 h. This means that the entire workflow -from blood withdrawal to bioinformatic analyses- can be performed in less than a working day").

Katsman et al.: "Detecting cell-of-origin and cancer-specific methylation features of cell-free DNA from Nanopore sequencing"
https://genomebiology.biomedcentral.com/articles/10.1186/s13059-022-02710-1
The autors report results for ONT sequencing of plasma from 7 healthy controls and 6 lung cancer patients. They show correlations between Illumina and ONT-based tumor fractions estimated by ichorCNA, similar as in Figure 1C of this study (however only based on 4 samples)
Furthermore, Katsman et al. show that nucleosome positioning is preserved in ONT-derived data, using CTCF binding motifs. They also compared fragment length profiles and end motifs between Illumina and ONT-derived data. Interestingly, Katsman et al. don't report longer fragment lenghts in ONT data compared to Illumina data.
The Katsman et al. study is cited, but not properly discussed in this manuscript.

Overall, given these two studies, the novelty of this manuscript is limited (and not properly discussed). Some of the most interesting aspects are the application to urine samples and, potentially, the ITSFASTR method (which is, however, not described in detail, and the github repository is not accessible).

The novelty of our work lies in:
1. the demonstration of the effective short-turnaround time,
2. the concordance of the tumor fraction between the short-read and long-read (not really described in detail in previous works),
3. the application to urine samples,
4. the presence of long tumor-derived DNA in both plasma and urine which were not described in prior studies.

In addition, we included new plasma samples and data from xenografted mice in our revised document, with a potential of informing with a very high specificity on the structural properties of tumor-derived cfDNA in these long size ranges.

As suggested, in our revised manuscript we are discussing further the 2 publications (that we previously cited in our document). However, we are partially in disagreement with the Reviewer’s analysis. The Martignano et al study is the proof of principle that Nanopore

sequencing could be used to retrieved copy number aberrations from 6 lung cancer plasma samples. Even if the short turn-around time is mentioned as one of the conceptual advantages of the work, it was not directly tested and validated in their study. The Katsman et al study shows indeed that nucleosome positioning could be retrieved from plasma samples but the observation of the cfDNA size profile does not reveal long fragments. This is in part due to the ONT kit and DNA preparation used by the authors and could be a limitation regarding the conclusions of the Katsman et al study regarding cfDNA structure. Thus, our study builds on these past works and provide new biological and translational data illustrating a potential use of the ONT in liquid biopsy.

2. Sample size / Tumor detection accuracy
##############################

As mentioned above, while the high correlation values to Illumina-derived data is promising, comparisons of tumor detection accuracy (sensitivity, specificity, ROC-AUC etc) with Nanopore vs conventional short-read sequencing are not provided. Those would be useful to assess how reliable the proposed method is. However, reliable comparisons would be hard to obtain given the limited sample size of this study.
We agree that estimating the clinical performance of the ONT approach for cancer detection would require a much larger number of samples and healthy controls. Recruiting, collecting and sequencing such large number of cancer and age-matched healthy samples is unfortunately not possible in the short timeframe offered for revising our manuscript. We listed the small cohort size as a clear limitation of our study, and further reinforce this point in the revised discussion. Nevertheless, we increased the number of cases sequenced in our manuscript (n= 6 cancer xenografts and n=3 healthy control) for comparison purposes.

3. ITSFASTR
#########
The ITSFASTR method could be interesting, but is not described in detail, and the github repository is not accessible. I therefore could not assess the quality and utility of this tool.
We apologize for this oversight. The ITSFASTR github repository is now publicly accessible. https://github.com/mouliere-lab/ITSFASTR We have also further detailed the description of the method in the method section.

MINOR POINTS:
Results, page 2:
• Rather report median read numbers per sample plus average coverage, instead of the total number of reads across all samples.
This is now corrected in the revised version of the manuscript. The modified sentence reads as follow: “The ONT platform yielded a median of 852,588.5 passed mapping reads with an average coverage of 0.108X, while NovaSeq sequencing a median of 69,998,356 mapping read-pairs with an average coverage of 4.562X (Table S1).”

• The down-sampling of Illumina reads should be mentioned also here in the main text, as this is an important aspect and has strong implications. Also, how many reads can Illumina vs Nanopore yield for comparable costs? One could down-sample also to a ratio of reads that represents similar costs.
The downsampling was intended to allow a fair comparison of the data between Illumina and ONT (otherwise ONT would be disadvantage by the higher coverage from the NovaSeq). In practice, the TF ichorCNA and size profile pre- and post-downsampling of the Illumina data were not exhibiting significant difference.

The estimated cost of MinION is 13.1$ / Gbp, with an entry cost of ~1k$. The estimated cost for NovaSeq S4 is 4.84$ / Gbp, with an entry cost of ~900k$. This is based on an estimation from our own cost, and based on the most update cost comparison for NGS from Albert Vilela blog.
The question is however more complex than the sole cost per Gb of data. The entry cost of the techniques is order of magnitude greater for Illumina machines, rendering their adoption in low to middle income labs without access to a sequencing facility difficult. Also, other Nanopore machines (e.g. Promethion) can produce a much larger number of reads, within the same cost range as NovaSeq machines (but obviously for an increased entry cost, and reduced deployability and flexibility).

• The 2 samples for which the Nanopore-derived tumor fraction is zero, but the Illumina derived tumor fraction is above zero should be discussed. Also, there seems to be an amplification artifact that is common between the two control samples.
These 2 samples have a lower coverage in comparison to other cases (less than 500k reads) that may cause the aberrant tumor fraction. To confirm this, we investigated the sensitivity of the ichorCNA tool depending on the coverage using in silico admixture of data at various read counts. The downsampled admixtures indicates that measurements below 500k reads start to become less accurate.

We added the following to the Methods section:
"*In silico admixture and down-sampling*
To generate a read count gradient, 12 long-read samples and their short-read pairs were used to create 25 in silico long- and corresponding short-read admixtures with 1M reads or read pairs using seqtk sample (v.1.3) with random seeds between 17-41 and the shell command cat. The resulting files were randomly down-sampled using seqtk sample (v.1.3) with random seeds between 75-51 to 1,000,000, 500,000, 100,000, 50,000, reads and read pairs, resulting in 25 samples each."

We also modified the results section to emphasize this point, and included the following sentence:
"To test if the ichorCNA tumor fraction estimates were affected by coverage, we created an *in-silico* admixture of 25 Illumina and 25 Nanopore samples with a tumor fraction of ~15% and downsampled them iteratively from 1M reads to 50,000 reads. We then computed the tumor fraction of each downsampled sample and found the lower limit of detection being between a depth 500,000 and 100,000 mapped reads for both short and long reads (Figure 1D)."

• The limitations of CNV calls from lower read depths compared to full-read-depth Illumina data should be discussed.
The previous comment addresses the issue of ichorCNA and lower coverage. Also, this is not a Nanopore vs Illumina issue per se, but more a problem of the sequencing read output per machines. Some Illumina machines have a limited output in reads (e.g. iSeq or MiniSeq), equivalent to Nanopore MinION. Some ONT machines have much higher read outputs (e.g. GridION and PromethION). We discussed further this point and included the revised discussion.

Results, page 3:
• The processing time of ~ 7h with a GPU could be indicated in the figure. A similar comparison (laptop / more advanced hardware) could then also be shown for the Illumina approach (probably less impact there?)

We initially detailed this graph more but decided to simplify it to a greater extend in order to facilitate understanding by a broader audience. We thus prefer to not detail this in the Figure and let the detail in the result section.

• Cite also the original study for this sentence: "Canonically, cfDNA in blood plasma has been described as short, fragmented molecules centered around 167 bp (and multiple) due to enzymatic cleavage linked to cell-death"
At our knowledge the first work evaluating this point is from Jahr and colleagues. We have included the corresponding citation (PMID: 11245480), in addition to the previous references. We also included a link to a review to allow readership to explore this point further if needed (PMID: 36380865).

• A version of the fragment size plots with a linear scale on the y axis would be nice. Otherwise, the total fraction of reads within certain proportions is hard to assess intuitively.
We have included below a plot of the median fragment size with a linear density scale on y axis. Source data are provided for reproducing the size analysis, and will allow modification of the scale if needed.

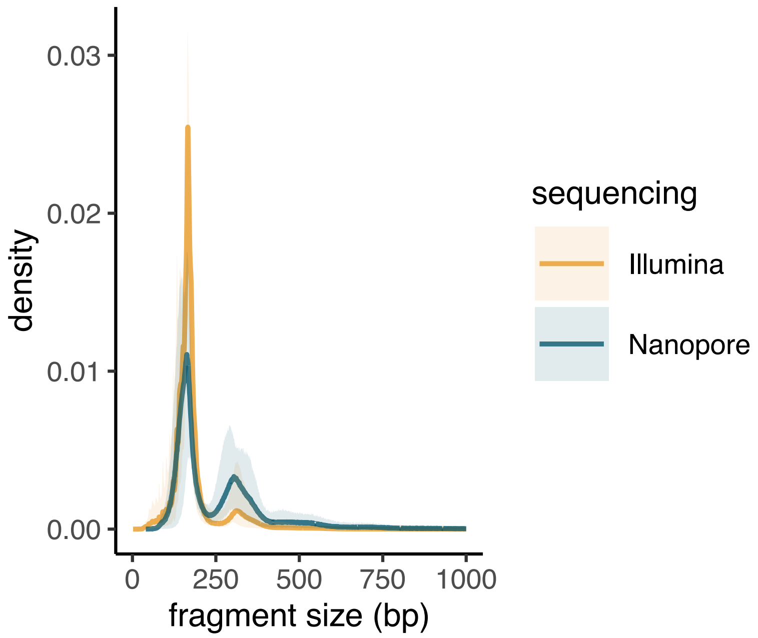

• "The diversity of the trinucleotides at the end of these long cfDNA fragments, determined using Gini index, was not different to the one in shorter (< 300bp) size ranges": Later on, the authors state that fragments with lengths <100 bp have decreased diversity, so "(<300bp)" must be inaccurate. Maybe 100-300bp is more accurate?
The sentence has been corrected as follow: “The diversity of the trinucleotides at the end of these long cfDNA fragments, determined using Gini index, was not different to the one in shorter (100-300bp) size ranges.” We thank the reviewer to point this typo.

• Figure 2B: The power for this analysis is low, as the number of sample is low and also no significant change between fragments of sizes <150 bp and 150-300 bp could be observed. Higher tumor fractions are expected in the <150 bp compared to 150-300 bp based on previous work, at least for Illumina data (Mouliere, Science Translational Medicine 2018). Can such an effect be observed for the collected Illumina data? In the aforementioned study, the authors used the t-MAD score to assess enrichment for tumor-derived fragments among short fragments. Would this be appropriate here as well?

We agree the cohort of samples included is small, and thus the power of analysis in Figure 2B was low. We decided to change our approach for this analysis. In short: we generated in silico admixtures from the different plasma or urine cancer samples to simulate more samples. These in silico admixtures were size-selected for the 3 size-range of interest (<150bp, 150-300bp and >300bp) and the tumor fraction calculated using the ichorCNA tool. The analysis confirms the presence of tumor signal in the 3 size ranges, with <150bp being significantly higher than the other range. We included the new figure (Fig 2B) in the manuscript and updated the result and methods section accordingly.
t-MAD could be used instead of ichorCNA as both approaches are providing a proxy for the tumor fraction using SCNA. Our internal testing does not demonstrate any sensitivity improvement in using t-MAD vs ichorCNA. We decided to use ichorCNA as this software is more broadly implemented in the liquid biopsy community than t-MAD.

Results, page 4:
• Suppl. Fig. 6-7: The sample names shown in these panels are different then for the CNA plots. Also, it would be easier to compare profiles between ONT and Illumina if the profiles were shown in a single plot per patient (ONT in blue, Illumina in yellow).
We have corrected the sample naming in the whole manuscript, and for this supplementary Figure in particular. Also, the corresponding plots have been modified as suggested to display a single plot per patient.

Discussion, page 4:
• "This has to be mitigated by the relative higher cost / Gb of the MinION data." This should be discussed in more detail. What are the costs / Gb? This comes back to my comments about downsampling.
The estimated cost of MinION is 13.1$ / Gbp, with an entry cost of ~1k$. The estimated cost for NovaSeq S4 is 4.84$ / Gbp, with an entry cost of ~900k$. This is based on an estimation from our own cost, and based on the most update cost comparison for NGS from Albert Vilela blog. We modified the sentence as follow: “The ONT sequencing platform MinION is highly deployable and has a much lower starting investment cost (currently ~1k $ against ~50k $ for a MiniSeq, ~250k $ for a NextSeq, and ~900k $ for a NovaSeq). This has to be mitigated by the relative higher cost / Gbp of the MinION data (~13.1 $/Gbp, compared to ~4.8 $/Gbp for Novaseq S4).”

Discussion, page 5:
• "Moreover, we observed that these long fragments contain tumor-derived ctDNA signal, which could be critical to recover in clinical conditions when such tumor signal is scarce (e.g. minimal residual disease detection)."Arguably sequencing deeper could be more important for MRD detection. Could the authors comment on that?
We agree that for MRD detection other higher depth ONT sequencing platform (e.g. Promethion) could be advantageous to generate higher coverage when the ctDNA TF is extremely low. Recent works also confirmed the potential of tumor-informed sequencing using an ONT platform. We included the following sentence in the discussion:
“Alternative approaches are under development to improve the recovery of SNV from plasma cfDNA using ONT sequencing (Marcozzi et al., 2021), and deeper sequencing coverage can be achieved using other ONT platforms which might be critical for tracking minimal residual disease.”.

• Typo in: "minutes amount"
We confirm this is corrected in the revised document.

Methods, page 7:

• "CollectInsertSizeMetrics with default parameters": This could be problematic, as CollectInsertSizeMetrics automatically truncates the histogram tail. Please ensure that fragments with sizes below 1000bp or 2000bp are not truncated. While this likely will not lead to a major change in the conclusions, if distributions were truncated, update the fragment size comparisons to ONT accordingly.
We re-run CollectInsertSizeMetrics with the HISTOGRAM_WIDTH=1000 setting, and corrected our Results and Methods sections accordingly. Indeed, this had no significant effect on our results.

Figure 1:
• A: CNA plots in panel A should be a separate panel. Also, please mark which plot comes from which technology more clearly.
We have not separated the SCNA plots but have indicated more clearly with an arrow from which technology they are coming from.

• B: Spell out "ratio" in the axis labels
This is modified in the revised Figure 1.

• C: "TF" is not explained in the figure legend.
TF is now defined in the revised figure legend.

• D: This could be shown in a stacked bar plot to provide information of the duration of sample preparation phases and computation phases separately.
As the sample preparation time will be more or less equivalent between the different technologies, the major difference coming from the sequencing and computation time, we feel that adding this information will not bring any significant new information to the overall turnaround time plot.

Figure 2:
• C: Is this for the downsampled or full-scale Illumina data?
The comparison is performed on downsampled Illumina data. We have now specified this in the figure legend of the revised manuscript.

Referee #3 (Comments on Novelty/Model System for Author):

Novelty: This work represents an emerging field of ONT-based cell-free DNA assays. While prior work in the field has been done before, this work directly compares Illumina to Nanopore and represents an important advance in the field.
Medical impact: There is interest in this type of assay to be applied in personalized medicine. Importantly, ONT MinION machines present an opportunity for rapid analysis of patient samples with fast turnaround time and (potentially) affordable cost. However, the authors here only present theoretical turnaround times of the ITSFASTR assay, and do not process patient samples as they are collected.

Referee #3 (Remarks for Author):

In this manuscript, van der Pol, Tantyo et al have developed a technical workflow to analyze copy number alterations from cell-free DNA sequenced via the Oxford Nanopore MinON machine.

The manuscript is well written, has strong merit and is an important contribution to the field. While certain aspects of the manuscript require revision and development, I highly recommend

this manuscript for eventual publication at EMM. There is a clinical need for fast, affordable cancer screening that might not be addressable with current ultra-high-throughput methods. While sequencing using high throughput (ex NovaSeq) sequencers is eventually more cost effective, a certain number of samples need to be sequenced at the same time, thus bottlenecking the diagnostic process. ONT sequencing and the ITSFASTR pipeline offer a solution to this bottleneck.

My comments:

1- Sample labels are uninformative. Cancer type and stage should be readily readable for each subplot.

We have changed the sample labels using the following template TYPE_STAGE_ID (e.g. LUAD_IV_001). This has been modified in the text, figures and tables.

2- Data for cancer-free controls is not used in any analysis, and should be used to at least compare cell-free DNA features (Tumor fraction, NDR, fragment lengths, and all other features looked at).

We have included an additional 3 plasma and 2 urine healthy controls (both with ONT and Illumina sequencing) in our analysis, and their cfDNA features are compared for each condition.

3- What portion of the Illumina workflow is more technically challenging, compared to ONT? The Illumina MiniSeq machine involves library preparation, dilution of libraries, and pipetting that diluted library in the Illumina cartridge. Is the ONT machine significantly simpler? Why would the Illumina MiniSeq machine require "assuming immediate availability of all required machines and staff to operate them". How would an ONT MinON be used?

Irrespective of the machine used (Illumina or ONT), both WGS protocols are using somewhat similar steps in their wetlab preparation. The ONT has technological options allowing an automatization of the preparation procedures (e.g. using a Voltrax) but they were not employed in our study. Practically the ONT MinION has 2 clear advantages versus a MiniSeq or NovaSeq: 1. The machine is much smaller and only requires a laptop for analysis. This could ultimately lead to much more flexible use at the patient's bed than the other machines more adapted for large genomic facilites. 2. The entry cost of the machine is magnitude lower than the MiniSeq and much lower than NovaSeq (around 1k euro for MinION, 150k euro for MiniSeq and >1M euro for NovaSeq). This could enable adoption in laboratories currently unable to afford such high entry cost.

4- Since the authors did not use ITSFASTR in an actual point-of-care setting this should not be mentioned.

We have removed mention to the point-of-care setting in the manuscript.

5- Mean and standard deviation (per sample) should be included in all sequencing metrics.

Based on other Reviewers comments we added the median readcount and the average coverage per sequencing type. The modified sentence reads as follow: “The ONT platform yielded a median of 852,588.5 passed mapping reads with an average coverage of 0.108X, while NovaSeq sequencing a median of 69,998,356 mapping read-pairs with an average coverage of 4.562X (Table S1).”

6- "Similar small and large genomic events" is qualitative. What is short? What is large?

This sentence has been changed and now reads as follow: “Similar genomic events could be observed in both short and long-read data as illustrated for one patient with lung cancer (Figure 1A), and for the whole dataset (Fig EV1).”.

7- The normalized coverage plots are interesting but do not provide a quantitative comparison between Short-read tech and nanopore. I would recommend some correlation metrics between the two sequencing modalities.

We computed the mean coverage in a ±1,000bp window around the TSS or NRR sites for short-read and long-read samples. We showed the correlation of the means in the revised manuscript as suggested (Figure 2G). The corresponding sentence reads as follow: “The mean coverage in the ±1,000bp vicinity of the TSS sites of interest show a moderate correlation between short- and long-read samples (Spearman, R=0.73, p=0.0047).”

8- Can the authors expand on the relevance of sequencing longer cfDNA fragments via ONT if the tumor fractions do not change depending on the length (F2B).

We identified and confirmed that a large fraction of tumor signal seems to be exhibited by cfDNA fragment longer than 300bp, even in urine samples. Even if their fraction is not enriched, it seems that these long fragments could be of importance for applications linked to very rare signal (when every tumor-derived DNA molecule matter, like for MRD). The complete extend of cfDNA tumor signal in this size range is still unknown and would require more characterization (e.g. with DNA isolation methods focused on recovering long fragments, comparison with PacBio sequencer). We could assume that these long fragments could also be of interest for application linked to structural variants, or altered topologies (e.g. extrachromosomal DNA). This is further discussed in the revised manuscript.

Referee #4 (Comments on Novelty/Model System for Author):

In this study, the authors perform and interesting comparison between important technologies in an adequate manner. However, it is already known that the ONT technologies are capable of obtaining information from long-size DNA. Nonetheless, is interesting to see its application in DNA from plasma and urine. The immediate clinical impact is not high since no mutations are being identify from liquid biopsy and the clinical impact of fragmentomics data is still unknown.

Referee #4 (Remarks for Author):

In this study, the authors include a series of plasma and urine samples from cancer patients to demonstrate that the ONT platforms are able to provide similar genomic profiles than conventional high-throughput platforms. The data shown herein is interesting for the liquid biopsy field applied to cancer and demonstrate that these easy-to-use and cheap platforms can provide important information from the tumor in a cost-efficient way.
I miss line numbering in the document. Please, see below my comments:

Minor comments:

- Abstract: The authors refer to lung patients in singular.

This has been corrected in the revised manuscript.

Major comments:

1. The authors should include a table with the patients´ clinicopatological characteristics. If they were included, the Supplementary Tables are not available to download.

We apologize if this was unclear but this table with the patient's clinicopathological characteristics was available in the extended dataset. We have now included this as a Table EV1 in the main document.

2. Overall, the authors should include information and graphs from adequate controls:

- Germline DNA: The authors should sequence the germline DNA from the patients, at least with the ONT platform. It is important to visualize patterns from potential contaminant germline DNA into plasma and urine samples.

We thank the Reviewer for this suggestion but viewing the focus on copy number aberration and fragmentomics in this study, the use of germline DNA would not be helpful. As single nucleotide mutations cannot be analyzed at this low coverage, filtering out SNP and other contaminants is not technically needed. Also, as germline DNA is genomic, there is no fragmentomic information to recover.

- Non-cancer controls: I also miss representations from the analysis of the sequencing data from non-cancer controls. They should be in supplementary materials. On top of that, the authors should sequence, at least with the ONT instrument, a number of plasma samples from unrelated healthy individuals.

We have included additional non-cancer healthy controls (n=3 plasma and 2 urine) in the revised manuscript. None of the controls have a detectable tumor fraction using ichorCNA (in comparison to 17/20 cancer cases).

We understand this is limited numbers but are constrain by the limited timeframe to revise the manuscript. This is in addition to the cancer cases previously included with low to undetectable levels of tumor signal using cfDNA. The data from these samples are better highlighted (see Figure EV2).

3. It would be interesting to sequence the corresponding tumors and compare the genomic profiles with the ones from liquid biopsy samples. Is it possible to identify tumor structural variations, single nucleotide variants or insertions and deletions using liquid biopsy?

Viewing the nature of the sequencing method used (low coverage WGS) and the ONT machines used (MinION) it is not possible to identify SNV, insertions or structural variants in either liquid biopsy or tissue DNA as the sequencing coverage will be too low (this is also the case for other sequencer machines). Recovering such genomic alterations would require a much higher depth which could be achieved with other ONT machines, at the cost of flexibility and speed (as it was previously demonstrated using tissue DNA with ONT). At our knowledge only one study has analyzed SNVs using a tumor-informed approach and ONT sequencing (PMID: 34887408, already cited in our work).

29th Jun 2023

Dear Dr. Mouliere,

Thank you for the submission of your revised manuscript to EMBO Molecular Medicine. We have now received the enclosed reports from the three initial referees. As you will see below, while referee #3 is satisfied with the revision, referees #2 and #4 still raise several major concerns on the work, including the small sample size, the lack of adequate controls, the unclear utility of >300 bp tumor-derived fragments detection, etc.
I went back to the referees to discuss whether their remaining concerns could be addressed in a straightforward manner and in a limited time.
The three referees agreed that further revisions should be invited, and that all their remaining concerns should be addressed. They concurred that incorporating more controls in the study should not pose a significant challenge, considering the rapid turnaround time and the importance of ensuring the robustness of the findings.
Regarding the utility of >300 bp, referee #3 mentioned that this could be addressed by either removing the analysis or by discussion.

We agree with the referees that these are essential points and would therefore like to invite you to revise the manuscript further to address all the referees' remaining concerns. As EMBO Press usually encourages one single round of revisions, please be aware that this will be the last chance for you to address these issues.

Moreover, please address the following editorial requests:
- Please provide up to 5 keywords.
- Please note that all corresponding authors should have an ORCID identifier.
- Please add a table of content (with page numbers) to your Appendix.

As part of the EMBO Publications transparent editorial process initiative (see our Editorial at http://embomolmed.embopress.org/content/2/9/329), EMBO Molecular Medicine will publish online a Review Process File (RPF) to accompany accepted manuscripts.
In the event of acceptance, this file will be published in conjunction with your paper and will include the anonymous referee reports, your point-by-point response and all pertinent correspondence relating to the manuscript. Let us know whether you agree with the publication of the RPF and as here, if you want to remove or not any figures from it prior to publication.
Please note that the Authors checklist will be published at the end of the RPF.

I look forward to receiving your revised manuscript.

Yours sincerely,

Lise Roth

Lise Roth, PhD
Senior Editor
EMBO Molecular Medicine

***** Reviewer's comments *****

Referee #2 (Remarks for Author):

In this revised manuscript, the authors now show convincing evidence that longer (>300 bp) cfDNA fragments contain fragments derived from tumors. 3 healthy plasma control samples were also added.
Many of my comments were addressed. Two more fundamental concerns remain. They may not be realistic to address as part of a revision - the decision about publication should be made with these limitations, but also the presented findings in mind. :

- Limited power when comparing tumor detection performance between ONT and Illumina, due to the small cohort size and very low number of control samples.
- The authors show that >300 bp tumor-derived fragments can be detected, but don't demonstrate the utility of detecting such

fragments.
A few other points below:
1. Novelty: In my opinion, a version of the author's reply to my original comment in the first review should be added to the manuscript's discussion section. Currently, the discussion section still may not make clear to reader which parts of the study are novel and where it extends on prior literature.
2. ITSFASTR: The source code is now available, which is helpful. However, in the manuscript, the method is still not described at all (at least I could not find it) and before looking on github it was very unclear to me which functionality this method has. A description should be added to the methods section. Please make sure to explain which parts of the analyses presented in the study is part of ITSFASTR.
3. Reviewer 3 wrote: "However, the authors here only present theoretical turnaround times of the ITSFASTR assay, and do not process patient samples as they are collected." Is this true? Please clarify here and in the manuscript. If turnaround times are theoretical, that would be an important limitation of the study.
4. Sample names: The sample names are still not uniform everywhere. See e.g. Figure EV2 vs S6. This should be corrected.
5. Fragment size plot with linear y-scale: This gives a useful overview at the first glance - I think it should be included in the supplement.

Referee #3 (Remarks for Author):

The authors have adequately addressed my prior comments.

Referee #4 (Comments on Novelty/Model System for Author):

Overall, the comparisons presented in this study are intriguing and relevant to the field. However, the medical impact is considerably limited due to the small sample size utilized. Moreover, the proper inclusion of adequate controls has not been adequately addressed, which raises concerns about the validity and reliability of the findings. To strengthen the significance and reliability of the study, it is crucial to address these limitations and ensure appropriate control measures are implemented in future investigations.

Referee #4 (Remarks for Author):

The study raises serious concerns regarding the adequacy of controls, which is essential for ensuring the reliability of the results. The inclusion of sequencing data from both tumor and germline DNA of the corresponding patients is crucial to strengthen the validity of the findings. Additionally, despite the authors' response, it is entirely possible to identify significant structural variations even with low coverage. Exploring this aspect would have added considerable value to the study, and it is disappointing that it was not adequately addressed, even partially, following my previous suggestion.

Dear Dr. Roth,

We would like to thank you and the Reviewers for the constructive feedback on our revised manuscript. The majority of the points raised in this round of revision can be addressed. We are however unsure on how to address some of the other concerns, or if they need to be addressed at all. These refer to the size of the cohort, the utility of fragments >300 bp, the use of appropriate controls, and the analysis of structural variations. In particular:

1. The size of the cohort. We understand that the modest size of our cohort (n=25 patients in total) is a potential limitation to support clinical claims. Our primary objectives were to evaluate if genomic tumor signal can be identified in plasma and urine using a fast turnaround time nanopore sequencing method, whether this signal can be compared to the signal retrieved using state of art Illumina sequencing and if additional layer of information can be retrieved from the data (fragmentomic features, long DNA fragments) using a reproducible code. Our current cohort is strong enough to support our claims on these points, especially after the addition of our xenograft models. For additional questions suggested by the reviewers, like the potential for cancer detection, we agree a much larger cohort of cases and controls will be needed. It is unclear from our perspective if adding ~10 additional samples would suffice to statistically support conclusions on this clinical point. Even if the nanopore is indeed fast, the same samples will have to be sequenced by Illumina as well (to match the rest of the manuscript), which is a much longer turnaround time and will double the costs, and further delay diffusion of the new data from our study. We would value feedback from the Editor and/or Reviewer on whether such additional samples are indeed necessary to support the main conclusions of our work, and if yes how many additional samples would be acceptable.

2. The utility of fragments >300bp. Beyond highlighting a utility, we think it is good to first report this discovery. At our knowledge no previous reports confirmed firmly that such fragments existed in circulation, mostly stating that ctDNA is "short". This observation goes against the general canon in the field. We understand that a potential clinical utility of such long fragments is not demonstrated in this work, and this is out of the scope of the current manuscript. We can however discuss further how the additional presence of such fragments could have an interest for 1. Recovering more tumor-derived molecules when ctDNA signal is minimal (e.g. MRD), 2. Phasing variants on a much long molecule than previously reported by the Diehn group, 3. Recovering in plasma topological structures known to be longer (e.g. eccDNA).

3. The use of appropriate controls. Reviewer #4 request the use of germline DNA from buffy coat stating as control. This is unfortunately irrelevant in the current context as such DNA need to be sheared for sequencing, thus losing any fragmentomic signal. Use of such controls would be more relevant in the context of mutation testing, which is not a feature of our method. We will not include germline DNA control in our revision. Regarding the inclusion of tissue DNA, unfortunately we do not have access for tissue DNA for the samples previously sequenced and included in our document and new samples would have to be included. More importantly the

comparison tissue DNA and plasma has previously been investigated in hundreds of publications and we do not feel it will add to our results. We would like to highlight that we included both positive controls and negative cfDNA controls with maximal level of specificity using animal models of cancer (which are better suited controls for this specific study), in addition to human plasma and urine samples from healthy individuals.

4. The analysis of structural variations. Reviewer #4 stated that structural variations can be retrieved from shallow WGS (ONT or Illumina). Even if very large events could indeed be retrieved, we question the potential robustness of such results. We do not think focal structural variations can be retrieved from shallow WGS. Maybe we do not understand well what the Reviewer suggested here? It would be good if this Reviewer could clarify what they mean by structural variations, and we will evaluate whether this can or cannot be addressed in a revision.

We feel that in overall addressing these additional concerns will not fundamentally change our results or better support our conclusions. As stated in our above reply, we would be happy to reply to some of these concerns, if this is needed, but we think other don’t require a reply.

We will be more than happy to discuss these points if you have any additional questions or suggestions.

Yours sincerely,
Florent Mouliere

Dear Florent,

Please accept my apologies for the delay in getting back to you in this busy time of the year: I was waiting to receive feedback from the 3 referees and to discuss again the different points brought up in your rebuttal with my colleagues here.
After careful consideration of yours and the referees' comments, we would like to propose the following:

1. Size of the cohort:

We strongly encourage you to include additional samples (as per your suggestion, ~10 would be acceptable), which should be analyzed by ONT only, not Illumina. This should take less time and further give you an opportunity to process samples in real-time (which would also allow to address the criticism on the fact that only theoretical turnaround times of the ITSFASTR method were described.)

2. Utility of fragments > 300 bp

We agree this part should remain in the manuscript, pending adequate discussion both in the manuscript and the point-by-point rebuttal letter.

3. Use of appropriate controls

Referees #2 and #3 agree that additional controls will not be necessary in a revised version of the manuscript.

However, the addition of new samples might also help addressing this point by including tissue controls.

4. Analysis of structural variations:

As suggested by referee #4, analysis of structural variations or large deletions through bioinformatic analyses should be feasible and would enhance the understanding of the genetic tumour landscape.

Additionally, please clearly discuss your results in light of previous publications and highlight the novelty of your work in this context.

I hope this revision plan seems reasonable to you. Please let me know if you would like to discuss further.

Looking forward to receiving your revised manuscript,

With kind regards,

Lise

Reply to editor:

1. Size of the cohort:

We strongly encourage you to include additional samples (as per your suggestion, ~10 would be acceptable), which should be analyzed by ONT only, not Illumina. This should take less time and further give you an opportunity to process samples in real-time (which would also allow to address the criticism on the fact that only theoretical turnaround times of the ITSFASTR method were described.)

We included 10 additional stage III lung cancer samples (in addition to the previously included stage IV cases). These were processed by Nanopore and Illumina sequencing to maintain coherence with the previous structure of the manuscript. As expected, our results confirm the significant correlation between the tumor fraction detected by Nanopore and Illumina sequencing and support our conclusions.

As a clarification the turnaround time of ITSFASTR that were previously reported are not theoretical. Our fastest run from plasma sampling to output of data was 12 h, even if the majority of the previous run had an average turnaround time of 24h.

2. Utility of fragments > 300 bp

We agree this part should remain in the manuscript, pending adequate discussion both in the manuscript and the point-by-point rebuttal letter.

At our knowledge no previous reports confirmed firmly that fragments >300 are abundant in circulation, mostly stating that ctDNA is “short”. This observation goes against the general canon in the field and it represents biological novelty . We understand that a potential clinical utility of such long fragments is not demonstrated in this work, and this is out of the scope of the current manuscript.

We can anticipate that the additional presence of such fragments could have an interest for 1. Recovering more tumor-derived molecules when ctDNA signal is minimal (e.g. MRD), 2. Phasing variants on much longer molecules than previously reported by the Diehn group, 3. Recovering in plasma topological structures known to be longer (e.g. eccDNA). This is discussed in the page 6 of the revised manuscript.

3. Use of appropriate controls

Referees #2 and #3 agree that additional controls will not be necessary in a revised version of the manuscript. However, the addition of new samples might also help addressing this point by including tissue controls.

We have included 10 additional lung cancer samples in our revised manuscript from an earlier stage of the disease. We would have to change our study protocol to get access to tumor material and thus are not able, viewing the short time frame of the revision, to include tissue controls. We however pointed out that the concordance of copy number aberrations between cfDNA and tissue DNA has previously been investigated in great depth in the literature, including using Nanopore data. We don’t consider further validation of this concordance to be novel or to improve the quality of the current manuscript.

4. Analysis of structural variations:

As suggested by referee #4, analysis of structural variations or large deletions through bioinformatic analyses should be feasible and would enhance the understanding of the genetic tumour landscape.

In our reply to referee #4 we discuss why such analysis is not feasible viewing the sequencing coverage used in our study.

Additionally, please clearly discuss your results in light of previous publications and highlight the novelty of your work in this context.
We further highlighted the previous work using Nanopore sequencing in liquid biopsy in our manuscript.

Reply to Reviewers:

Referee #2 (Remarks for Author):

In this revised manuscript, the authors now show convincing evidence that longer (>300 bp) cfDNA fragments contain fragments derived from tumors. 3 healthy plasma control samples were also added.
Many of my comments were addressed.
We thank the Reviewer for their constructive comments.

Two more fundamental concerns remain. They may not be realistic to address as part of a revision - the decision about publication should be made with these limitations, but also the presented findings in mind. :
- Limited power when comparing tumor detection performance between ONT and Illumina, due to the small cohort size and very low number of control samples.
We included 10 additional lung cancer samples (stage III) that further confirm our prior observations. Our objective being the correlation of Illumina and Nanopore data, our results are now supported by 35 samples. We understand and agree, that this is not enough for confirming a potential in terms of detection, but feel that such objective is beyond the scope of this manuscript.

- The authors show that >300 bp tumor-derived fragments can be detected, but don't demonstrate the utility of detecting such fragments.
At our knowledge no previous reports confirmed firmly that such fragments are abundant in circulation, mostly stating that ctDNA is "short". This observation goes against the general canon in the field, and we feel it is an important biological novelty. We agree that a potential clinical utility of such long fragments is not demonstrated in this work. We have however discussed further on page 6 how the presence of such fragments could have an interest for: 1. Recovering more tumor-derived molecules when ctDNA signal is minimal (e.g. MRD), 2. Phasing variants on a much longer molecules than previously reported by the Diehn group, 3. Recovering in plasma topological structures known to be longer (e.g. eccDNA).

A few other points below:

1. Novelty: In my opinion, a version of the author's reply to my original comment in the first review should be added to the manuscript's discussion section. Currently, the discussion section still may not make clear to reader which parts of the study are novel and where it extends on prior literature.
   This point was covered in the introduction of the previously revised manuscript, where we mentionned the previous publications on cfDNA and Nanopore. We revisited the elements of novelty of our work in comparison to the prior references in the discussion of our revised manuscript.

2. ITSFASTR: The source code is now available, which is helpful. However, in the manuscript, the method is still not described at all (at least I could not find it) and before looking on github it was very unclear to me which functionality this method has. A description should

be added to the methods section. Please make sure to explain which parts of the analyses presented in the study is part of ITSFASTR.
We added the following line to the Methods section: "*The ITSFASTR tool*
ITSFASTR (InTegrated Sequence and Fragmentome AnalysiS Time Reduction) was developed by our group (https://github.com/mouliere-lab/ITSFASTR) and used in this study for integrating in a single fast-turnaround time pipeline the read pre-processing, alignment, copy number analysis and tumor fraction estimation, fragmentomic and nucleosome positioning analysis. ITSFASTR is compatible with short and long-read sequencing technologies."

3. Reviewer 3 wrote: "However, the authors here only present theoretical turnaround times of the ITSFASTR assay, and do not process patient samples as they are collected." Is this true? Please clarify here and in the manuscript. If turnaround times are theoretical, that would be an important limitation of the study.
We confirm the turnaround time for the Nanopore and Novaseq Illumina are not theoretical. We included the calculated turnaround time for the Miniseq to provide a fair comparison to an Illumina machine of equivalent output to the Nanopore MinION but we could not experimentally confirm the time for MiniSeq – this is described in the corresponding part of the results section. Our average turnaround time from plasma to result is 24h using Nanopore MinIon (12h being our fastest run).

4. Sample names: The sample names are still not uniform everywhere. See e.g. Figure EV2 vs S6. This should be corrected.
This has been corrected in the revised document.

5. Fragment size plot with linear y-scale: This gives a useful overview at the first glance - I think it should be included in the supplement.
We prefer to keep a log scale for the y axis on this summary figure, as we consider this being more intuitive. However, we provided the data source to the plot for replotting.

Referee #3 (Remarks for Author):

The authors have adequately addressed my prior comments.
We thank the Reviewer for their constructive comments.

Referee #4 (Comments on Novelty/Model System for Author):

Overall, the comparisons presented in this study are intriguing and relevant to the field. However, the medical impact is considerably limited due to the small sample size utilized. Moreover, the proper inclusion of adequate controls has not been adequately addressed, which raises concerns about the validity and reliability of the findings. To strengthen the significance and reliability of the study, it is crucial to address these limitations and ensure appropriate control measures are implemented in future investigations.
We thank the Reviewer for their constructive comments.

Following the editor's recommendation, we included 10 additional lung cancer samples (stage III) in our revised manuscript but we have not included additional controls. The central objective of the manuscript was the comparison of Illumina and Nanopore data in terms of tumor fraction estimation

and fragmentomic analysis. Our new results confirm our initial observation now on samples from 35 individuals.
Inclusion of a large cohort of controls and cancer cases will be indeed important for confirming a medical impact of a Nanopore-based sequencing approach, but is far beyond the scope of this manuscript more focused on feasibility and biology exploration. We highlighted the modest cohort size as a limitation in terms of clinical conclusion in the discussion of the manuscript.

Referee #4 (Remarks for Author):

The study raises serious concerns regarding the adequacy of controls, which is essential for ensuring the reliability of the results. The inclusion of sequencing data from both tumor and germline DNA of the corresponding patients is crucial to strengthen the validity of the findings.
As previously indicated, germline DNA controls are irrelevant in the context of fragmentomic analysis, and would be more adapted for the analysis of SNV (which is not possible viewing the low depth of the Nanopore or Illumina sequencing in this study). Germline DNA originates from intact cells, while fragmentomic information is retrieved from DNA cleaved during cell death. This cleavage is non-random and carries information about the cell-of-origin.
Additionally, matched tissue samples were not available for the samples included in the study. Comparison of tissue DNA and plasma cfDNA for SNV and CNV detection has previously been investigated in hundreds of publications, showing a high degree of correlation, repeating this would not add to our results. We would like to highlight that we included positive cfDNA controls with maximal level of specificity using animal models grafted with human cancer (which are better suited controls for this specific study), and human plasma and urine samples from healthy individuals, as negative controls. Based on these factors and following the editor's recommendation, we have not included germline DNA analysis in our revised manuscript.

Additionally, despite the authors' response, it is entirely possible to identify significant structural variations even with low coverage. Exploring this aspect would have added considerable value to the study, and it is disappointing that it was not adequately addressed, even partially, following my previous suggestion.
We performed shallow whole genome sequencing on our samples (average genome coverage 0.1X) which is adapted for copy number and fragmentomics analysis. The accurate detection of structural variations (SV) requires a much higher sequencing coverage. For example, a prior work analyzing chromothripsis found an optimum for SV detection of ~14X coverage for long Nanopore reads (PMID: 29109544, specifically SupFig14.). In silico simulations from another study confirmed that coverage above >10x is needed to have minimum confidence in SV analysis (PMID: 32813322). Moreover, in liquid biopsy, the coverage needed for detecting tumor-specific SVs increases as the tumor fraction of the plasma and urine samples decreases, and may require ultra-deep or targeted sequencing. In addition, the sequencing library preparation kit used in our study represents a source of false positives. The kit uses ligation to attach sequencing adapters to the end of the cfDNA fragments. This ligation step is capable of producing artefactual structural variations by ligating two positionally distant fragments. These would show up as breakpoints or deletions in the genomic alignment. Lacking the coverage to exclude these stochastic events we consider the somatic variation detection unreliable that would reduce the quality of our manuscript.
Thus, taken these different elements together, we respectfully disagree with the Reviewer's opinion that SV analysis is robust at such low coverages.

10th Oct 2023

Dear Dr. Mouliere,

Thank you for the submission of your manuscript to EMBO Molecular Medicine. We have now received the feedback from referees #2 and #4 on your revised manuscript. As you will see below, they support publication of your work pending minor revisions. We will therefore be able to accept your manuscript once the following will be addressed:

1/ Referees' comments:
- Please address the remaining referees' concerns experimentally or by an adequate discussion in the text.

2/ Manuscript text:
- Please address the queries from our data editors that you will find in the Data edited file (attached) and keep in track changes mode any new modification. Please note that the data editors worked on the V2 version of your manuscript, thus please format any new legend/text following the same standards.
- Please suggest up to 5 keywords.
- The manuscript sections should be in the following order: Title page - Abstract & Keywords - Introduction - Results - Discussion - Materials & Methods - Data Availability - Acknowledgments - Disclosure Statement & Competing Interests - References - Figure Legends - Expanded View Figure Legends.
- Material and methods:
o Study design: please include the full statement that the experiments conformed to the principles set out in the WMA Declaration of Helsinki and the Department of Health and Human Services Belmont Report
o Xenograft model: please indicate the origin of the mice, and provide age, gender, housing and husbandry conditions.
o Please add a statistics section including statements on sample size, blinding, randomization, inclusion/exclusion criteria (it should reflect the checklist).
- Data availability: Please remove the Code Availability heading and include the entire section under Data Availability. Please note that we need an active link to the EGA dataset before publication of the manuscript.
- Author contributions: CRediT has replaced the traditional author contributions section because it offers a systematic machine-readable author contributions format that allows for more effective research assessment. Please remove the Authors Contributions from the manuscript and use the free text boxes beneath each contributing author's name in our system to add specific details on the author's contribution. More information is available in our guide to authors.
- Rename "Conflict of Interest" to "Disclosure statement and competing interests": We updated our journal's competing interests policy in January 2022 and request authors to consider both actual and perceived competing interests. Please review the policy https://www.embopress.org/competing-interests and update your competing interests if necessary.
- Please remove the Table EV legend from the manuscript file.

3/ Source Data:
Source Data should be uploaded as one file per figure (files for each figure should be zipped together). It appears that Source Data for Figure 1B is missing.

4/ Checklist:
- please fill in the housing and husbandry conditions
- please fill in the section "sample definition and in-laboratory replication"

5/ Synopsis:
In introduced minor modifications in your text, please let me know if you agree with the following or amend as you see fit:

Cell-free DNA (cfDNA), a rising biomarker in oncology, can be used as a readout from a liquid biopsy. Current analytical methods employ short-read DNA sequencing technologies. We designed a long-read Nanopore sequencing technique to analyze liquid biopsy samples.

- Tumor-derived cfDNA could be detected in the plasma and urine of cancer patients using Nanopore sequencing, demonstrating sensitivity equivalent to short-read sequencing.
- The turn-around time from sample collection to obtaining results was <24 hours when utilizing the deployable MinION Nanopore platform.
- Nanopore sequencing successfully captured fragmentomics signal, including long cfDNA fragments (>300 bp), in both plasma and urine samples.
- The long cfDNA fragments (ranging from >300 bp to 8055 bp) contained tumor-derived molecules, as confirmed in human and xenograft samples.

6/ As part of the EMBO Publications transparent editorial process initiative (see our Editorial at

http://embomolmed.embopress.org/content/2/9/329), EMBO Molecular Medicine will publish online a Review Process File (RPF) to accompany accepted manuscripts.
This file will be published in conjunction with your paper and will include the anonymous referee reports, your point-by-point response and all pertinent correspondence relating to the manuscript. Let us know whether you agree with the publication of the RPF and as here, if you want to remove or not any figures from it prior to publication.
Please note that the Authors checklist will be published at the end of the RPF.

I look forward to receiving your revised manuscript.

Yours sincerely,

Lise Roth

Lise Roth, PhD
Senior Editor
EMBO Molecular Medicine

***** Reviewer's comments *****

Referee #2 (Remarks for Author):

New samples have been added, which is good, but it looks like some figures have not been updated accordingly. See e.g. Fig 1 B, EV1, 2G. Also 2A?

The tumor fraction of sizes 150-300 has changed substantially since the last version (now all <0.1, before all ~ >0.125). The longer fragments now show significantly larger tumor content than the 150-300 fragments (in the previous version, they had significantly lower tumor content). Is this due to using a new set of 12 samples to generate these data? In either case, some form of additional robustness analysis should be performed.
Also, it is not clear from the figure legend that these are simulated data, which is very confusing.

The legend for Figure 2G is missing.

Referee #4 (Remarks for Author):

In response to the authors' comments, I would like to provide the following feedback:

Comments:

1. The authors have introduced new samples, which enhance the value of the study. However, the inclusion of samples from healthy individuals remains limited. Incorporating additional control samples could have bolstered the results of this study.

2. I recommend that the authors refer to the publication with PMID: 34006333. In this study, the authors successfully detected structural variants (SVs) using significantly lower coverage than what is mentioned in their response. It would be intriguing to explore whether there are any indications of somatic SVs in biofluid samples with a high ctDNA content.

Minor changes:

3. Additionally, I noticed that the authors stated that there is no prior evidence of long DNA fragments in circulation, and this finding is novel to their study. I would like to point out some examples where long cfDNA fragments have already been identified in biofluids:

PMID: 35587130
PMID: 34873045
PMID: 34516908

They should discuss that long fragment presence in cfDNA was previously observed.

Editor comments:

1/ Referees' comments:
- Please address the remaining referees' concerns experimentally or by an adequate discussion in the text.
This is addressed in the revised document.

2/ Manuscript text:
- Please address the queries from our data editors that you will find in the Data edited file (attached) and keep in track changes mode any new modification. Please note that the data editors worked on the V2 version of your manuscript, thus please format any new legend/text following the same standards.
We have addressed the queries from the data editors in the revised document.

- Please suggest up to 5 keywords.
Keywords have been included.

- The manuscript sections should be in the following order: Title page - Abstract & Keywords - Introduction - Results - Discussion - Materials & Methods - Data Availability - Acknowledgments - Disclosure Statement & Competing Interests - References - Figure Legends - Expanded View Figure Legends.
We have now reordered the section as indicated.

- Material and methods:
o Study design: please include the full statement that the experiments conformed to the principles set out in the WMA Declaration of Helsinki and the Department of Health and Human Services Belmont Report
This is included.
o Xenograft model: please indicate the origin of the mice, and provide age, gender, housing and husbandry conditions.
This is now described.
o Please add a statistics section including statements on sample size, blinding, randomization, inclusion/exclusion criteria (it should reflect the checklist).
This section has been included in the revised document.

- Data availability: Please remove the Code Availability heading and include the entire section under Data Availability. Please note that we need an active link to the EGA dataset before publication of the manuscript.
This is corrected. The link to the EGA dataset is active.

- Author contributions: CRediT has replaced the traditional author contributions section because it offers a systematic machine-readable author contributions format that allows for more effective research assessment. Please remove the Authors Contributions from the manuscript and use the free text boxes beneath each contributing author's name in

our system to add specific details on the author's contribution. More information is available in our guide to authors.
This is corrected.

- Rename "Conflict of Interest" to "Disclosure statement and competing interests": We updated our journal's competing interests policy in January 2022 and request authors to consider both actual and perceived competing interests. Please review the policy https://www.embopress.org/competing-interests and update your competing interests if necessary.
This is corrected.

- Please remove the Table EV legend from the manuscript file.
This is corrected.

3/ Source Data:
Source Data should be uploaded as one file per figure (files for each figure should be zipped together). It appears that Source Data for Figure 1B is missing.
This is not possible as the data source for Fig2A and 3A are grouped in the same excel file. I have uploaded each file independently. Source data for Figure 1B has been included in the revised submission.

4/ Checklist:
- please fill in the housing and husbandry conditions
- please fill in the section "sample definition and in-laboratory replication"
This is now updated.

5/ Synopsis:
In introduced minor modifications in your text, please let me know if you agree with the following or amend as you see fit:

Cell-free DNA (cfDNA), a rising biomarker in oncology, can be used as a readout from a liquid biopsy. Current analytical methods employ short-read DNA sequencing technologies. We designed a long-read Nanopore sequencing technique to analyze liquid biopsy samples.

- Tumor-derived cfDNA could be detected in the plasma and urine of cancer patients using Nanopore sequencing, demonstrating sensitivity equivalent to short-read sequencing.
- The turn-around time from sample collection to obtaining results was <24 hours when utilizing the deployable MinION Nanopore platform.
- Nanopore sequencing successfully captured fragmentomics signal, including long cfDNA fragments (>300 bp), in both plasma and urine samples.
- The long cfDNA fragments (ranging from >300 bp to 8055 bp) contained tumor-derived molecules, as confirmed in human and xenograft samples.

I am happy with this version of the synopsis.

6/ As part of the EMBO Publications transparent editorial process initiative (see our Editorial at http://embomolmed.embopress.org/content/2/9/329), EMBO Molecular Medicine will publish online a Review Process File (RPF) to accompany accepted manuscripts.
This file will be published in conjunction with your paper and will include the anonymous referee reports, your point-by-point response and all pertinent correspondence relating to the manuscript. Let us know whether you agree with the publication of the RPF and as here, if you want to remove or not any figures from it prior to publication.
Please note that the Authors checklist will be published at the end of the RPF.
I am happy with the publication of the RPF.

Reply to reviewers:

Referee #2 (Remarks for Author):

New samples have been added, which is good, but it looks like some figures have not been updated accordingly. See e.g. Fig 1 B, EV1, 2G. Also 2A?
Fig 1B is an example from one patient and is correct. Fig 2A and 2G are correct. The plasma from Figure EV1 were indeed only showing the stage IV lung cancer cases and legend has been updated to reflect this.

The tumor fraction of sizes 150-300 has changed substantially since the last version (now all <0.1, before all ~ >0.125). The longer fragments now show significantly larger tumor content than the 150-300 fragments (in the previous version, they had significantly lower tumor content). Is this due to using a new set of 12 samples to generate these data? In either case, some form of additional robustness analysis should be performed. Also, it is not clear from the figure legend that these are simulated data, which is very confusing.
We thank the reviewer for this observation. Indeed, the downwards shift in the overall tumor fraction of the simulated samples is due to the relatively low tumor fraction of the newly added samples from earlier stage lung cancer (mean: 0.0576 SD: 0.059). This could be due to nature of the newly added samples (stage III cancer, instead of stage IV in the previous version). In the revised manuscript we amended the figure legend with the requested information.

The legend for Figure 2G is missing.
We thank the Reviewer to point to this. This has been included in the revised version.

Referee #4 (Remarks for Author):
In response to the authors' comments, I would like to provide the following feedback:

1. The authors have introduced new samples, which enhance the value of the study. However, the inclusion of samples from healthy individuals remains limited. Incorporating additional control samples could have bolstered the results of this study. We agree including more healthy controls and cancer cases would have increase the impact of our work, we unfortunately are not in capacity to do this on a short-term notice. The current cohort of samples and controls however is sufficient to support our claims.

2. I recommend that the authors refer to the publication with PMID: 34006333. In this study, the authors successfully detected structural variants (SVs) using significantly lower coverage than what is mentioned in their response. It would be intriguing to explore whether there are any indications of somatic SVs in biofluid samples with a high ctDNA content.
Here the authors are using tumor and normal tissue DNA and subsequent filtering to identify tumor-specific SVs using Nanopore sequencing. These confirmed SVs are then tested on cfDNA from ascites, plasma and germline DNA using breakpoint PCR or qPCR. A conservative filtering of the tumor tissue sequencing data removed ~97% of all detected SVs. After filtering they could validate on the average 10 somatic SVs per sample using breakpoint PCR. This work clearly confirms the possibility of recovering somatic SVs from low coverage tumor tissue, but not liquid biopsies. As indicated in our previous responses, lacking tumor-specific and healthy tissue (germline) data, shallow WGS is unreliable to recover somatic SV from plasma or urine samples.

Minor changes:
3. Additionally, I noticed that the authors stated that there is no prior evidence of long DNA fragments in circulation, and this finding is novel to their study. I would like to point out some examples where long cfDNA fragments have already been identified in biofluids:
PMID: 35587130
PMID: 34873045
PMID: 34516908

They should discuss that long fragment presence in cfDNA was previously observed.
The presence of long cfDNA in plasma is known since the works of Jahr et al (2001). The cited PMID by the Reviewer are confirming this prior work using a range of sequencing techniques. Our work demonstrates that a large fraction of these DNA is effectively tumor-derived using samples from animal models, and not solely coming from non-cancer cells as it is commonly thought in the liquid biopsy field (and shown by the PMID cited by the Reviewer). Thus, we confirm our claim of novelty on the observation that tumor-derived cfDNA can be very long in plasma (which the prior reports were not able to confirm).
We have already discussed the prior report PMID: 34873045 that identified using sequencing long cfDNA fragments (cf discussion page 7, reference Yu et al, 2021). We included reference PMID: 35587130 which complement this prior reference using cancer samples, but not provide specific characterization of tumor-derived ctDNA. PMID: 34516908 recover fragments up to 500bp which is not novel (cf prior works from Mouliere et al, 2018, STM).

17th Oct 2023

Dear Dr. Mouliere,

Thank you for sending the revised files. I am pleased to inform you that your manuscript is now accepted for publication in EMBO Molecular Medicine!

Before we can send your manuscript to our publisher, please note that information on gender and age of the mice at the time of experiment is missing. Please add this information in the materials and methods; you may then send me the manuscript file via email and I'll upload it in the submission system.

Congratulations on your work!

With kind regards,

Lise Roth

Lise Roth, Ph.D
Senior Editor
EMBO Molecular Medicine

Follow us on Twitter @EmboMolMed
Sign up for eTOCs at embopress.org/alertsfeeds

*** *** *** IMPORTANT INFORMATION *** *** ***

SPEED OF PUBLICATION
The journal aims for rapid publication of papers, using using the advance online publication "Early View" to expedite the process: A properly copy-edited and formatted version will be published as "Early View" after the proofs have been corrected. Please help the Editors and publisher avoid delays by providing e-mail address(es), telephone and fax numbers at which author(s) can be contacted.

Should you be planning a Press Release on your article, please get in contact with embomolmed@wiley.com as early as possible, in order to coordinate publication and release dates.

LICENSE AND PAYMENT:

All articles published in EMBO Molecular Medicine are fully open access: immediately and freely available to read, download and share.

EMBO Molecular Medicine charges an article processing charge (APC) to cover the publication costs. You, as the corresponding author for this manuscript, should have already received a quote with the article processing fee separately. Please let us know in case this quote has not been received.

Once your article is at Wiley for editorial production you will receive an email from Wiley's Author Services system, which will ask you to log in and will present you with the publication license form for completion. Within the same system the publication fee can be paid by credit card, an invoice, pro forma invoice or purchase order can be requested.

Payment of the publication charge and the signed Open Access Agreement form must be received before the article can be published online.

PROOFS

You will receive the proofs by e-mail approximately 2 weeks after all relevant files have been sent o our Production Office. Please return them within 48 hours and if there should be any problems, please contact the production office at

embopressproduction@wiley.com.

Please inform us if there is likely to be any difficulty in reaching you at the above address at that time. Failure to meet our deadlines may result in a delay of publication.

All further communications concerning your paper proofs should quote reference number EMM-2022-17282-V4 and be directed to the production office at embopressproduction@wiley.com.

EMBO Press Author Checklist

Corresponding Author Name: Florent Mouliere
Journal Submitted to: EMBO Molecular Medicine
Manuscript Number: EMM-2022-17282

USEFUL LINKS FOR COMPLETING THIS FORM
The EMBO Journal - Author Guidelines
EMBO Reports - Author Guidelines
Molecular Systems Biology - Author Guidelines
EMBO Molecular Medicine - Author Guidelines

Reporting Checklist for Life Science Articles (updated January
This checklist is adapted from Materials Design Analysis Reporting (MDAR) Checklist for Authors. MDAR establishes a minimum set of requirements in transparent reporting in the life sciences (see Statement of Task: 10.31222/osf.io/9sm4x). Please follow the journal's guidelines in preparing your
Please note that a copy of this checklist will be published alongside your article.

Abridged guidelines for figures
1. Data
The data shown in figures should satisfy the following conditions:
- the data were obtained and processed according to the field's best practice and are presented to reflect the results of the experiments in an accurate and unbiased manner.
- ideally, figure panels should include only measurements that are directly comparable to each other and obtained with the same assay.
- plots include clearly labeled error bars for independent experiments and sample sizes. Unless justified, error bars should not be shown for technical
- if n<5, the individual data points from each experiment should be plotted. Any statistical test employed should be justified.
- Source Data should be included to report the data underlying figures according to the guidelines set out in the authorship guidelines on Data

2. Captions
Each figure caption should contain the following information, for each panel where they are relevant:
- a specification of the experimental system investigated (eg cell line, species name).
- the assay(s) and method(s) used to carry out the reported observations and measurements.
- an explicit mention of the biological and chemical entity(ies) that are being measured.
- an explicit mention of the biological and chemical entity(ies) that are altered/varied/perturbed in a controlled manner.
- the exact sample size (n) for each experimental group/condition, given as a number, not a range;
- a description of the sample collection allowing the reader to understand whether the samples represent technical or biological replicates (including how many animals, litters, cultures, etc.).
- a statement of how many times the experiment shown was independently replicated in the laboratory.
- definitions of statistical methods and measures:
  - common tests, such as t-test (please specify whether paired vs. unpaired), simple χ2 tests, Wilcoxon and Mann-Whitney tests, can be unambiguously identified by name only, but more complex techniques should be described in the methods section;
  - are tests one-sided or two-sided?
  - are there adjustments for multiple comparisons?
  - exact statistical test results, e.g., P values = x but not P values < x;
  - definition of 'center values' as median or average;
  - definition of error bars as s.d. or s.e.m.

Please complete ALL of the questions below.
Select "Not Applicable" only when the requested information is not relevant for your study.

Materials

Newly Created Materials	Information included in the manuscript?	In which section is the information available? (Reagents and Tools Table, Materials and Methods, Figures, Data Availability Section)
New materials and reagents need to be available; do any restrictions apply?	Not Applicable	

Antibodies	Information included in the manuscript?	In which section is the information available? (Reagents and Tools Table, Materials and Methods, Figures, Data Availability Section)
For **antibodies** provide the following information:<br>- Commercial antibodies: RRID (if possible) or supplier name, catalogue number and or/clone number<br>- Non-commercial: RRID or citation	Not Applicable	

DNA and RNA sequences	Information included in the manuscript?	In which section is the information available? (Reagents and Tools Table, Materials and Methods, Figures, Data Availability Section)
**Short novel DNA or RNA including primers, probes**: provide the sequences.	Not Applicable	

Cell materials	Information included in the manuscript?	In which section is the information available? (Reagents and Tools Table, Materials and Methods, Figures, Data Availability Section)
**Cell lines:** Provide species information, strain. Provide accession number in repository **OR** supplier name, catalog number, clone number, and/**OR** RRID.	Yes	Materials and Methods
**Primary cultures:** Provide species, strain, sex of origin, genetic modification status.	Not Applicable	
Report if the cell lines were recently **authenticated** (e.g., by STR profiling) and tested for mycoplasma contamination.	Yes	Materials and Methods

Experimental animals	Information included in the manuscript?	In which section is the information available? (Reagents and Tools Table, Materials and Methods, Figures, Data Availability Section)
**Laboratory animals or Model organisms:** Provide species, strain, sex, age, genetic modification status. Provide accession number in repository **OR** supplier name, catalog number, clone number, **OR** RRID.	Yes	Materials and Methods
**Animal observed in or captured from the field:** Provide species, sex, and age where possible.	Not Applicable	
Please detail **housing and husbandry conditions**.	Yes	Materials and Methods

Plants and microbes	Information included in the manuscript?	In which section is the information available? (Reagents and Tools Table, Materials and Methods, Figures, Data Availability Section)
**Plants:** provide species and strain, ecotype and cultivar where relevant, unique accession number if available, and source (including location for collected wild specimens).	Not Applicable	
**Microbes:** provide species and strain, unique accession number if available, and source.	Not Applicable	

Human research participants	Information included in the manuscript?	In which section is the information available? (Reagents and Tools Table, Materials and Methods, Figures, Data Availability Section)
If collected and within the bounds of privacy constraints report on age, sex and gender or ethnicity for all study participants.	Yes	Material and Methods

Core facilities	Information included in the manuscript?	In which section is the information available? (Reagents and Tools Table, Materials and Methods, Figures, Data Availability Section)
If your work benefited from core facilities, was their service mentioned in the acknowledgments section?	Yes	ackowledgements

Design

Study protocol	Information included in the manuscript?	In which section is the information available? (Reagents and Tools Table, Materials and Methods, Figures, Data Availability Section)

If study protocol has been **pre-registered, provide DOI in the manuscript**. For clinical trials, provide the trial registration number **OR** cite DOI.	Not Applicable	
Report the **clinical trial registration number** (at ClinicalTrials.gov or equivalent), where applicable.	Not Applicable	

Laboratory protocol	Information included in the manuscript?	In which section is the information available? (Reagents and Tools Table, Materials and Methods, Figures, Data Availability Section)
Provide DOI OR other citation details if **external detailed step-by-step protocols** are available.	Yes	Material and Methods

Experimental study design and statistics	Information included in the manuscript?	In which section is the information available? (Reagents and Tools Table, Materials and Methods, Figures, Data Availability Section)
Include a statement about **sample size** estimate even if no statistical methods were used.	Yes	Material and Methods
Were any steps taken to minimize the effects of subjective bias when allocating animals/samples to treatment (e.g. **randomization procedure**)? If yes, have they been described?	Not Applicable	
Include a statement about **blinding** even if no blinding was done.	Yes	Material and Methods
Describe **inclusion/exclusion criteria** if samples or animals were excluded from the analysis. Were the criteria pre-established?<br><br>If sample or data points were omitted from analysis, report if this was due to attrition or intentional exclusion and provide justification.	Yes	Material and Methods
For every figure, are **statistical tests** justified as appropriate? Do the data meet the assumptions of the tests (e.g., normal distribution)? Describe any methods used to assess it. Is there an estimate of variation within each group of data? Is the variance similar between the groups that are being statistically compared?	Yes	Figure legends, Materials and Methods

Sample definition and in-laboratory replication	Information included in the manuscript?	In which section is the information available? (Reagents and Tools Table, Materials and Methods, Figures, Data Availability Section)
In the figure legends: state number of times the experiment was **replicated** in laboratory.	Yes	Figure legends
In the figure legends: define whether data describe **technical or biological replicates**.	Yes	Figure legends

Ethics

Ethics	Information included in the manuscript?	In which section is the information available? (Reagents and Tools Table, Materials and Methods, Figures, Data Availability Section)
Studies involving **human participants**: State details of **authority granting ethics approval** (IRB or equivalent committee(s), provide reference number for approval.	Yes	Materials and Methods
Studies involving **human participants**: Include a statement confirming that **informed consent** was obtained from all subjects and that the experiments conformed to the principles set out in the WMA Declaration of Helsinki and the Department of Health and Human Services Belmont Report.	Yes	Materials and Methods
Studies involving **human participants:** For publication of **patient photos**, include a statement confirming that consent to publish was obtained.	Not Applicable	
Studies involving experimental **animals**: State details of **authority granting ethics approval** (IRB or equivalent committee(s), provide reference number for approval. Include a statement of compliance with ethical regulations.	Yes	Materials and Methods
Studies involving **specimen and field samples:** State if relevant **permits** obtained, provide details of authority approving study; if none were required, explain why.	Not Applicable	

Dual Use Research of Concern (DURC)	Information included in the manuscript?	In which section is the information available? (Reagents and Tools Table, Materials and Methods, Figures, Data Availability Section)
Could your study fall under dual use research restrictions? Please check biosecurity documents and list of **select agents and toxins** (CDC): https://www.selectagents.gov/sat/list.htm	Not Applicable	
If you used a select agent, is the security level of the lab appropriate and reported in the manuscript?	Not Applicable	
If a study is subject to dual use research of concern regulations, is the name of the **authority granting approval and reference number** for the regulatory approval provided in the manuscript?	Not Applicable	

Reporting

The MDAR framework recommends adoption of discipline-specific guidelines, established and endorsed through community initiatives. Journals have their own policy about requiring specific guidelines and recommendations to complement MDAR.

Adherence to community standards	Information included in the manuscript?	In which section is the information available? (Reagents and Tools Table, Materials and Methods, Figures, Data Availability Section)
State if relevant guidelines or checklists (e.g., **ICMJE, MIBBI, ARRIVE, PRISMA**) have been followed or provided.	Not Applicable	
For **tumor marker prognostic studies,** we recommend that you follow the **REMARK** reporting guidelines (see link list at top right). See author guidelines, under 'Reporting Guidelines'. Please confirm you have followed these guidelines.	Not Applicable	
For **phase II and III randomized controlled trials**, please refer to the **CONSORT** flow diagram (see link list at top right) and submit the CONSORT checklist (see link list at top right) with your submission. See author guidelines, under 'Reporting Guidelines'. Please confirm you have submitted this list.	Not Applicable	

Data Availability

Data availability	Information included in the manuscript?	In which section is the information available? (Reagents and Tools Table, Materials and Methods, Figures, Data Availability Section)
Have **primary datasets** been deposited according to the journal's guidelines (see 'Data Deposition' section) and the respective accession numbers provided in the Data Availability Section?	Yes	Data availability section
Were **human clinical and genomic datasets** deposited in a public access-controlled repository in accordance to ethical obligations to the patients and to the applicable consent agreement?	Yes	Data availability section
Are **computational models** that are central and integral to a study available without restrictions in a machine-readable form? Were the relevant accession numbers or links provided?	Yes	Data availability section
If publicly available data were reused, provide the respective **data citations in the reference list**.	Not Applicable	
